# Evaluation of Sampling Algorithms Used for Bayesian Uncertainty Quantification of Molecular Dynamics Force Fields

**DOI:** 10.1021/acs.jctc.4c00130

**Published:** 2024-06-26

**Authors:** Abhishek T. Sose, Troy Gustke, Fangxi Wang, Gaurav Anand, Sanjana Pasupuleti, Aditya Savara, Sanket A. Deshmukh

**Affiliations:** † Department of Chemical Engineering, Virginia Tech, Blacksburg, Virginia 24060, United States; ‡ Oak Ridge National Laboratory, Oak Ridge, Tennessee 37830, United States

## Abstract

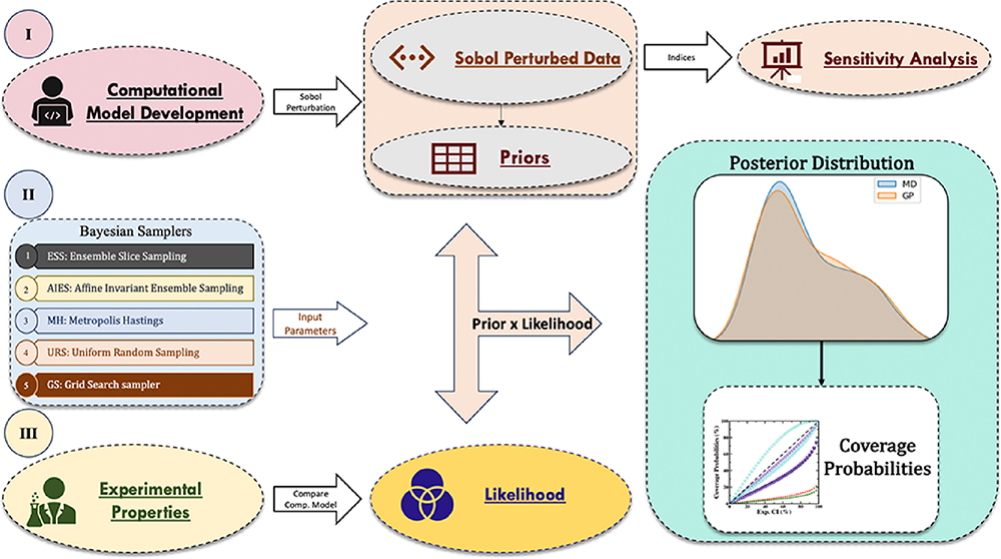

New Bayesian parameter estimation methods have the capability to enable more physically realistic and reliable molecular dynamics (MD) simulations by providing accurate estimates of uncertainties of force-field (FF) parameters and associated properties. However, the choice of which Bayesian parameter estimation algorithm to use has not been widely investigated, despite its impact on the effective exploration of parameter space. Here, using a case example of the Embedded Atom Method (EAM) FF parameters, we investigated the ramifications of several of the algorithm choices. We found that Ensemble Slice Sampling (ESS) and Affine-Invariant Ensemble Sampling (AIES) demonstrate a new level of superior performance, culminating in more accurate parameter and property estimations with tighter uncertainty bounds, compared to traditional methods such as Metropolis-Hastings (MH), Gradient Search (GS), and Uniform Random Sampler (URS). We demonstrate that Bayesian Uncertainty Quantification with ESS and AIES leads to significantly more accurate and reliable predictions of the FF parameters and properties. The results suggest that ESS and AIES should be used to obtain more accurate parameter and uncertainty estimations while providing deeper physical insights.

## 1 Introduction

The advancements in experimental and computational methods in materials science and engineering have significantly improved the generation of accurate and reliable data sets. While frequentist interpretations have historically dominated data set analysis and parameter estimation, there has been a notable shift towards Bayesian methods.^1−3^ This transition can be attributed to rises in computing power, advancements in theoretical understanding, and Bayesian methods’ unique ability to quantify uncertainty during data-driven solution estimation. Bayesian uncertainty quantification (BUQ) of integrated model parameters can provide deeper insights into the reliability and robustness of both experimental and computational methods.^4−6^

In BUQ-based parameter estimation, the solution values comprise a probability distribution, termed the posterior distribution.^7^ The maximum of the distribution represents the most probable set of parameters, and in this work, it corresponds to seeking the most physically correct set of parameters when all uncertainties are accounted for.^8^ Determining this distribution of parameter values requires exploration of the available parameter space. This exploration is most commonly performed using Markov Chain Monte Carlo (MCMC) sampling algorithms,^9,10^ which puts greater emphasis on exploring more probable regions of the parameter space.^11^ There are multiple MCMC sampling algorithms that are available. Metropolis-Hastings (MH) is prevalent due to its simplicity, historical usage, straight-forward implementation, and low computational cost.^12−15^ Ensemble MCMC samplers like Affine-Invariant Ensemble Sampling (AIES)^16,17^ and Ensemble Slice Sampling (ESS)^18,19^ are better suited to explore high-dimensional spaces. A direct and systematic comparison of their performance in scientific problems is critical for selecting an algorithm for complex parameter spaces, offering not only improved solutions but also unraveling important physical insights through a well-explored and well-characterized posterior. Here, we test the hypothesis that AIES and ESS will be superior for obtaining physically meaningful parameter estimates along with narrow uncertainties for molecular dynamics (MD) simulation force-field (FF) parameters and properties, as they can efficiently explore high-dimensional spaces.

Recognizing the limitations of conventional model parameter optimization^20−24^ to account for uncertainties, use of BUQ has been increasing in MD simulations research to develop FF parameters, and these studies are crucial for understanding atomic- and molecular-level interactions in materials.^25−36^ An ideal set of FF parameters would result in precise MD simulations. There is the potential to achieve these FF parameters by integrating BUQ with appropriate sampling algorithms. Most importantly, BUQ can enable UQ of both parameters and predicted properties, ensuring more reliable and robust models with accurate predictions. To classify the performance of FF after physically meaningful parameter estimation, we define the FF produced as “True” if it accurately predicts the target properties. Additionally, simulations with the FF also have uncertainties when produced by BUQ, and these uncertainties can be *tightened*, *unchanged*, or *loosened* uncertainties with respect to the original uncertainties of any target physical property. A model is thus True-Tightened with respect to a particular property if the final property estimate (the posterior distribution) is consistent with the present-day beliefs of the ground truth and also achieves a narrower uncertainty distribution for that property’s output (e.g., at least >20% tighter than the initial uncertainty for that property). A model is thus True-Unchanged if the final property estimate (the posterior distribution) is consistent with the present-day beliefs of the ground truth, and there is little change in the uncertainty distribution for that property (e.g., between 20% narrower and 20% broader). A model is thus True-Loosened if the final property estimate (the posterior distribution) is consistent with the present-day beliefs of the ground truth and moves to a broader uncertainty distribution for that property (e.g., >20% broader). In the context of MD simulations, obtaining a model that is a True-Tightened FF is an ideal outcome: such an FF not only consistently matches physical predictions but even enhances our precision relative to what was known from only experiments.

In this work, we employ different samplers for BUQ to estimate the uncertainty of the input parameters for the MD model and the simulated properties. Specifically, our test case model is the MD’s Embedded Atom Method (EAM)^37,38^ potentials to model all-atom gold systems by accounting for their pairwise interactions and the electron density from neighboring atoms in which the atom is embedded. We highlight that our primary objective was to compare the performance of different BUQ sampling algorithms to explore the space of the 14 FF parameters with the secondary goal of obtaining True-Tightened FF models (Section S1. I of the Supporting Information). One of the major advantages of employing the Bayesian approach is that it not only provides uncertainties for the properties but also for the model parameter values. We examined the three-mentioned MCMC algorithms: MH, AIES, and ESS; as well as two non-MCMC sampling algorithms: grid search (GS) and uniform random sampler (URS). Each sampler was tasked with exploring the posterior distribution, with the solutions judged on the structural, mechanical, and thermophysical properties produced by MD simulations. We found that ESS and AIES were able to develop accurate and robust True-Tightened FF models.

## 2 Methodology

### 2.1 Model Development

**Figure 1:**
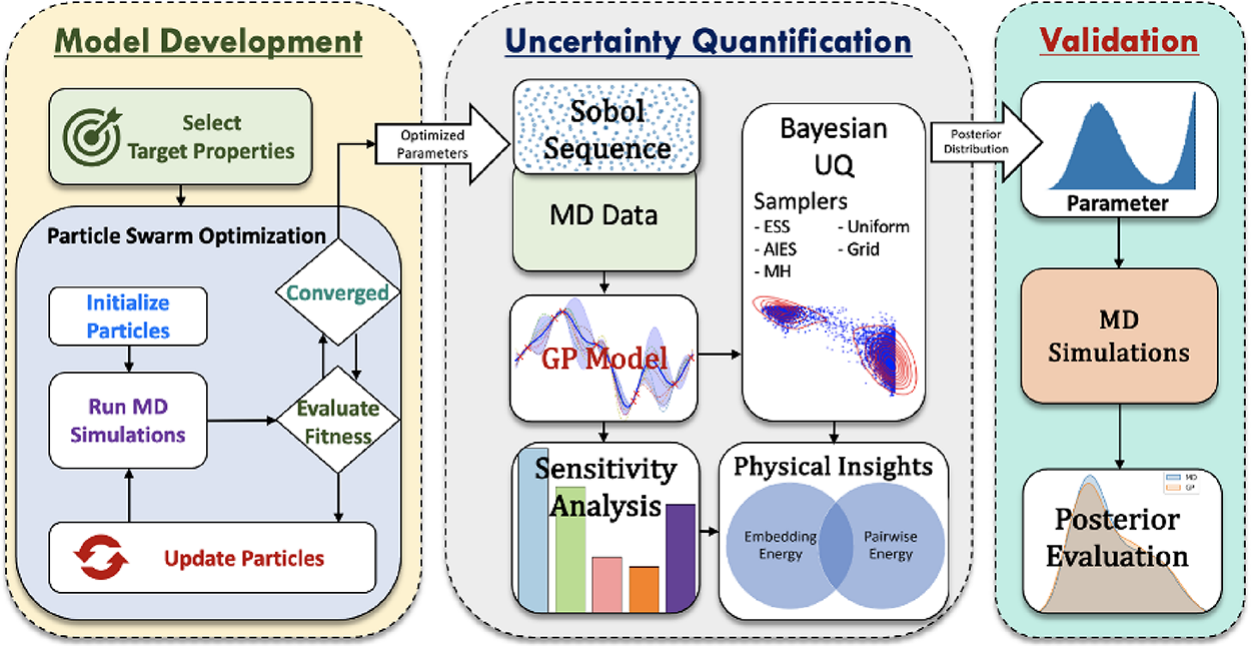
Flowchart of the Bayesian Uncertainty Quantification (BUQ) framework. This shows the MD model development, data generation for Gaussian Process Regression (GPR) model training using Sobol sequencing, integration of GPR models with sampling algorithms to perform BUQ of the EAM potential, and last, the validation of their posterior distribution.

We first performed a conventional parameter optimization with particle swarm optimization (PSO)^21,39−41^ and the LAMMPS MD simulation package to develop 14 parameters of EAM FF for an all-atom gold model (Figure 1).^21,42,43^ The model was optimized toward the structural, mechanical, and thermophysical properties. Particularly, we evaluated the 10 properties for FCC gold: (i) Cohesive energy refers to the amount of energy required to completely separate the atoms in a solid metal, indicating the strength of the atomic bonds. (ii) Density is the mass of the metal per unit volume, often expressed in grams per cubic centimeter. Elastic constants like (iii) C11, (iv) C12, and (v) C44 are measures of the stiffness or rigidity of the metal in all three directions. (vi) The bulk modulus represents the resistance of a material to compression, indicating its ability to withstand changes in volume under pressure. (vii) Poisson’s ratio is a measure of the ratio of lateral contraction to longitudinal extension when a material is stretched. Surface tensions at the (viii) 100, (ix) 110, and (x) 111 surfaces refer to the energy required to increase the surface area of the metal along their crystallographic directions, indicating the surface stability of the metal. These properties were selected because of the availability of the multiple reports on experimental and Density Functional Theory (DFT) as well as their ease of calculations in MD simulations.^44−50^ Experimental/DFT values for the density and cohesive energy are generally precise and exhibit little variation. This is followed by simpler elastic constants like C11 and C12, as well as the bulk modulus. However, we observed greater variability in experimental DFT values for the Poisson's ratio and C44. Surface tension values, on the other hand, show the most variability, likely due to differing techniques used for calculating the surface tension and their sensitivity to surrounding conditions. These multiple sources further enabled us to calculate their uncertainties, which was used as experimental uncertainty in our BUQ calculations (more details shown in Section S1. I of the Supporting Information).^51−56^

PSO iteratively refined the parameters based on the sum of squared residuals for the 10 target properties. Note, this conventional optimization cannot comprehensively account for the uncertainties of the target properties.^7,57^ Hence, the BUQ facilitates the thorough integration of experimental (inherent) uncertainties into the target values during the model development process. The next section sheds light on the implementation of BUQ in this study.

### 2.2 Bayesian Uncertainty Quantification (BUQ) Framework

Bayesian parameter estimation enables inclusion of uncertainties for finding more physically realistic solutions^8^ and performing BUQ. However, the uncertainty quantification within BUQ requires large quantities of sampling of parameter permutations, and thus, the BUQ process can require significant computational expense. One best practice when the goal is to characterize the best parameters for complex systems with BUQ is to mitigate this computational expense by starting with a preoptimized initial point, as was done here using PSO to obtain the initial point. Alternatively, one could take existing choices of FF parameters of interest (such as from existing literature) and perform BUQ in the region of the posterior region around that initial point. An additional strategy for mitigating the computational expense is to use intermediate surrogate models for the extensive sampling required for BUQ, and such intermediate surrogate models were used here. In this work, we also perform Sobol Sensitivity analysis around the initial point to gain insights and to ensure that the surrogate models faithfully capture the training data distributions. If one includes these additional steps for practical and accurate BUQ in computationally intensive applications, then the BUQ framework encompasses the following three stages:

#### 2.2.1 Sobol Sensitivity

To further investigate the sensitivity of these properties, a Sobol sequence is used to perturb the input parameter set from some perturbation percent to give evenly distributed parameter sets using the following equation:
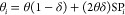
1where SP_*i*_ is the *i*^th^ point in the Sobol sequence, θ is the parameter set, and δ is the perturbation percent. In this work, a perturbation percent of 5% and 5000 samples are used to study a large local space around the optimized parameter set with appropriate point density.

Conducting a sensitivity analysis employing Sobol sampling with data derived from all-atom model simulations and diverse parameter configurations, the PSO-derived set of FF parameters was perturbed, using a Sobol sequence (which is a quasi-random sampling) within 5% bounds, to create 5000 sets of parameters to perform MD simulations.^58,59^ Using these MD results, we determine the sobol sensitivity indices, given by eq 2.
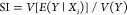
2

Sobol sensitivity indices, or Sobol measures, quantify variance in output properties relative to input parameters. They reveal higher-order interactions’ impact on property variances. Using the above formula, the effect of higher-order interactions can be assessed by comparing first-order and total-order indices. Sobol sensitivity indices provide a variance-based analysis, distinguishing changes in parameters’ effects on properties. First-order indices directly link parameter changes to property variance, while higher-order indices identify variance contributions from parameter interactions. Total variance contributions sum to 1, indicating first-order relations’ dominance. These quantify variance in output properties concerning input parameters, revealing higher-order interactions’ impact on variances. This approach provides insights into our model, attributing variance contributions to specific parameters and their interactions. Therefore, it emphasizes thefirst-order relations for our system, which are critical.

#### 2.2.2 GPR Modeling

We employ a machine learning model, Gaussian Process regression (GPR), that is trained on the Sobol data, which can serve as a surrogate for MD simulations. GPR is a flexible, nonparametric machine learning (ML) model and can provide probabilistic predictions and compatibility with Bayesian inference. This compatibility allows GPR to naturally incorporate prior knowledge and provide a full probabilistic description of the uncertainty in the predictions, making it an ideal candidate for uncertainty quantification in MD simulation models using Bayesian approaches. To ensure efficient training and inference without sacrificing accuracy, the GPR models were trained on a subset of 4000 data points, while the remaining 1000 points were employed as testing data. Finally, the best kernel was chosen and the surrogate model was trained on all 5000 points, to be employed further in Bayesian analysis. (Details in Section S1.II of the Supporting Information).^60,61^

The GPR models were used as surrogates for MD simulations, which enabled us to extensively sample and explore the uncertainties of the property predictions using the BUQ algorithms implemented in the PEUQSE software package (the software package formerly named CheKiPEUQ).^7,57^ The purpose of the GPR surrogate models is to expedite the BUQ in this framework. In problems we have worked on, this approach is scalable up to ∼10 parameters on a conventional computer and ∼15 parameters on a supercomputer. For computational modeling that is less computationally expensive than GPR predictions, this surrogate modeling step may be omitted as it would be unnecessary. However, nearly all scientific computing that is beyond algebraic will be outpaced by GPR surrogate models. Thus, BUQ can likely be accelerated by GPR for the vast majority of scientific computing applications.

#### 2.2.3 Bayesian Uncertainty Quantification Input

To develop our understanding of the underlying model dynamics and intricacies by assessing the dependence of parameters, θ, relative to the experimental/DFT observations, *Y*_obs_, we developed a Bayesian framework.^62^ Specifically, we created posterior distributions for individual parameters through Bayes theorem, which can be written as shown in eq 3.^7,27^
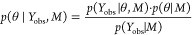
3

In eq 3, *p*(θ|*Y*_obs_, *M*) is the posterior probability distribution of the FF parameters given the observed data and a model, *M*; *p*(*Y*_obs_|θ, *M*) is the likelihood function of observing the experimental data from the given model, *p*(θ|M) is the prior distribution of the FF parameters given a model, and *p*(**Y*_obs_*|*M*) is the evidence, which is assumed to be constant. To mitigate potential biases in the system, a bound uniform prior distribution was chosen with the same bounds established during the Sobol sequence sampling for each parameter. This approach ensured that the prior distribution was consistent with the underlying parameter space and avoided an undue influence on the posterior distribution. We can assume that the observed data and subsequent model predictions follow the prediction error equation:
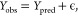
4where *Y*_pred_ is the predicted value from a model, *M*. Here, the error term consists of a summation of possible errors given by ϵ = ϵ_D_ + ϵ_SM_, where ϵ_D_ is the measurement error in experimental data, and ϵ_SM_ is the modeling error of surrogate models (i.e., GPR models), which also accounts for the computational error in MD simulations, ϵ_MD_. The total error of the network is often approximated to be Gaussian in nature (i.e., ϵ ∼ *N*(0, σ_error_^2^)), and we did so here, although this assumption is not necessary for our framework. Here, the likelihood function served as the measure of fit between the experimental observations and MD predictions. The exploration of more probable posterior regions is then dependent, in part, on this likelihood function.

Achieving Bayesian parameter estimation and additional uncertainty quantification (UQ) for the model through the integration of the GPR model and PEUQSE, a Bayesian tool designed for parameter estimation. The BUQ likelihood function was constructed as a multivariate Gaussian distribution. The errors in experiments/DFT along with the GPR model errors were incorporated into the BUQ. For BUQ, we ran appropriate extents of parameter exploration with several algorithms for comparison. MH was run with a single Markov chain (walker) for 2 million samples (Section S1. II of the Supporting Information). AIES and ESS were run with 56 walkers for 8 and 1.6 million samples, respectively. ESS was run with differential moves. URS was run for 15 million samples, and GS was run for ∼4.8 million samples. The differences in sample size and walker counts arise due to each algorithm’s design and also reflect the sampling used for exploration of any additional postreriour modes that were found by the sampler.^12,16−19^ For the purposes of comparing the performance of the samplers, comparable computational resources were used for each sampler's first exploratory search.

## 3 Results and Discussion

### 3.1 Model Development Using Particle Swarm Optimization (PSO)

Initial PSO-derived 14 EAM parameters exhibited good agreement between MD properties and with those obtained from reported experiments and DFT calculations (Section S1. I and Tables S3 and S4 in the Supporting Information).^53−56^ Moreover, the parameters developed in this study outperform the majority of the reported all-atom gold models in almost all structural, mechanical, and thermophysical properties (Table S4 in the Supporting Information).^63−68^ The question then becomes: if we want to perform uncertainty quantification of the force-field parameters using a Bayesian approach, which sampling algorithm would be used to explore such a complex parameter space?

### 3.2 Sobol Sensitivity Indices

**Figure 2:**
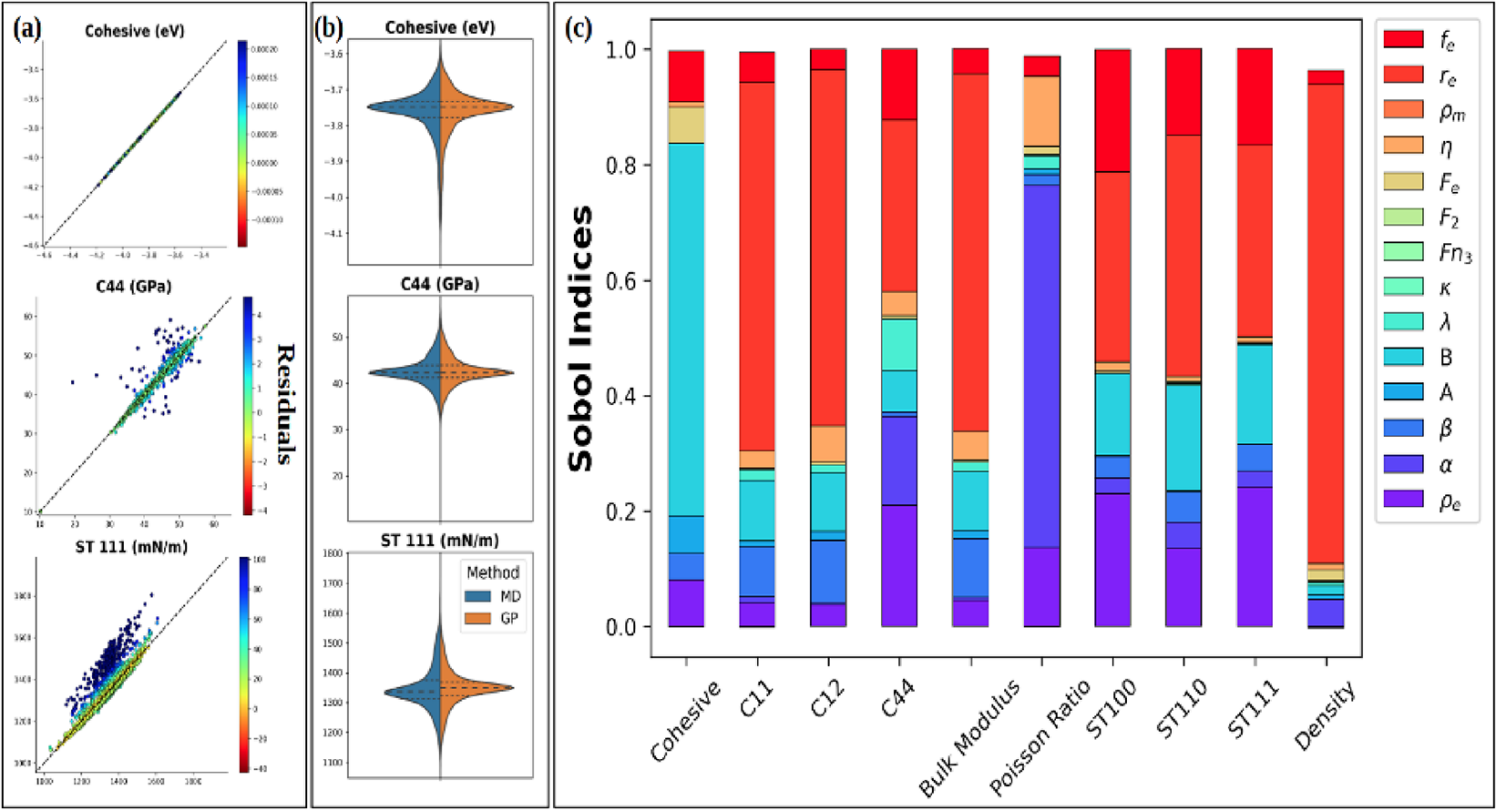
Assessment of GPR model performance by comparing GPR predictions with MD predictions based on Sobol Sequence data. (a) Parity plots demonstrate high accuracy, (b) while violin plots indicate that the property value distributions from MD simulations are effectively captured by the GPR models. (c) First-order Sobol sensitivity indices indicating variation of properties being correlated with changes in FF parameters.

We first investigated the starting model which had been optimized by PSO. Within a 5% perturbation of parameters bounds, the coefficients of variation for each property were less than ∼7.5% (Figure 2 and Section S1. II of the Supporting Information). The lower accuracy of the GPR for the (111) surface reflects the inherent challenges associated with modeling surface properties using EAM.^69−72^ Several studies in the literature have reported that most EAM potential forms for various metals underestimate the surface energies.^37,73,74^ This has been attributed to the surrounding environment of the surface atoms, which are in low-electron-density regions, and EAM potential inability to model these large electron-density gradients at the surfaces. Despite significant efforts to improve the environment of the surrounding atoms by introduction of new parameters in EAM potential, predictions of surface energies remain challenging.^75−77^ In the present study, performing Sobol perturbation within 5% of the original data set further highlights challenges associated with surface energy calculations. Additionally, it reveals that even minor variations in FF parameters can affect surface energy in ways that are unpredictable and lack discernible patterns. This results in lower quality data for training GPR models.

Using the GPR models, variance-based sensitivity analysis was performed, to assess which properties varied as a parameter was changed and plotted with a Sobol indices scale (Figure 2c). This analysis shows that parameter *B* largely influenced *E*_coh_ and parameter *A* was correlated to the Poisson ratio. Moreover, as anticipated, the equilibrium bond distance (*r*_e_), the electron density at which embedding energy is the lowest (ρ_e_),^76^ and the proportional coefficient for electron-density calculation (*f*_e_)^76^ were each correlated to most properties.

### 3.3 Bayesian Uncertainty Quantification Analysis

**Figure 3:**
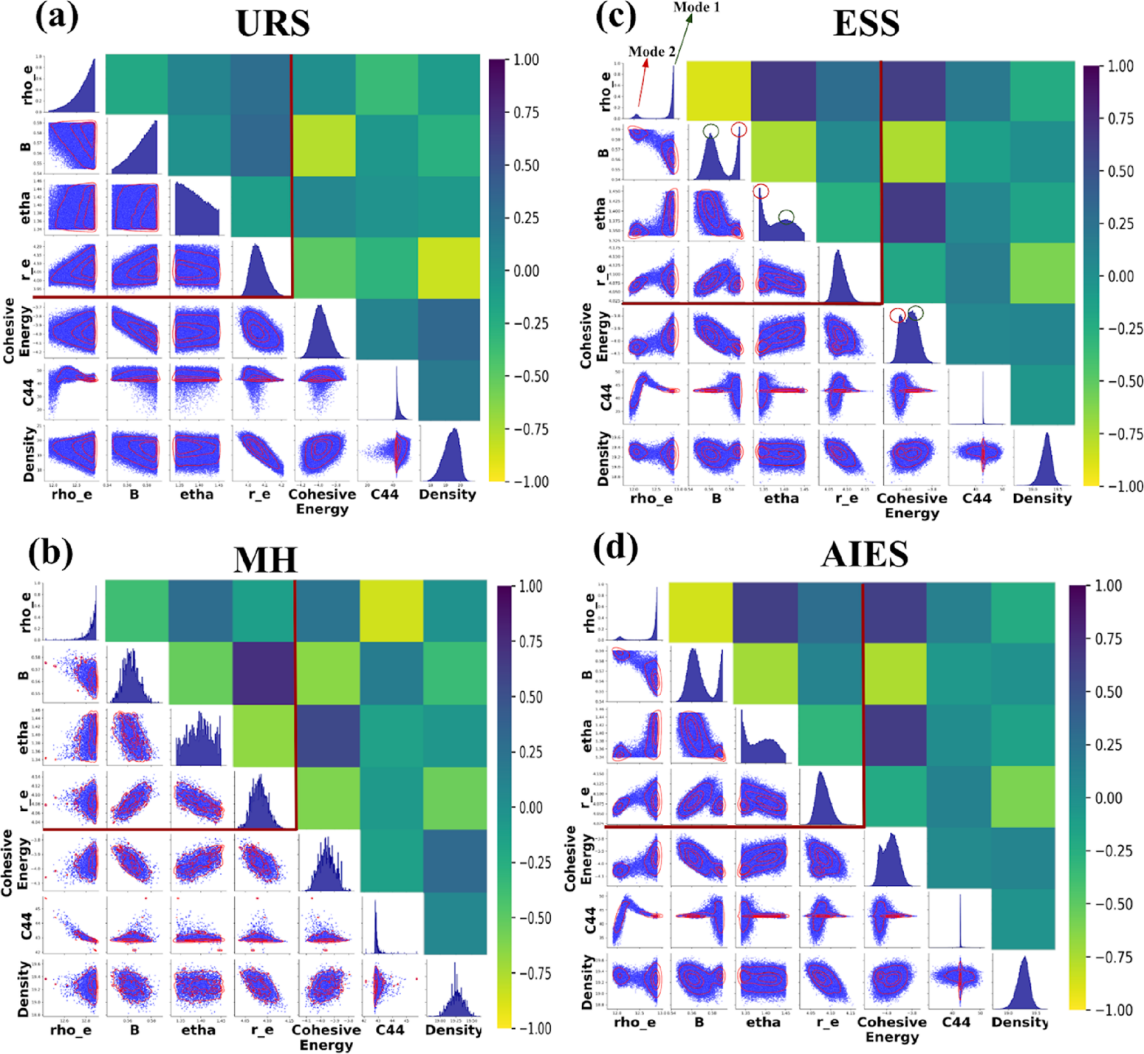
Posterior distributions from (a) URS, (b) MH, (c) ESS, and (d) AIES within corner plots and heatmaps of Pearson correlation coefficients, illustrating the correlation of selected properties and selected input parameters. The depicted parameter space is reduced to show the impactful parameters and multimodality found during BUQ. The red-colored internal border distinguishes parameters from properties, such that the upper-left region of each panel (4 x 4 grid) shows correlations among parameters, whereas the bottom-right region of each panel (3 × 3 grid) shows correlations among properties, and the bottom-left region of each panel (3 x 4 grid) shows property–parameter correlations. The two modes found by ESS during BUQ are designated when clearly visible within the distribution plots at the diagonal of panel c.

Next, BUQ was performed to find more physically realistic solutions and assess the robustness of the final model parameters in determining the properties. Multiple BUQ sampling algorithms, called samplers, were investigated. These BUQ samplers were broken into three classes: non-MCMC (URS, GS), single-walker MCMC (MH), and ensemble MCMC (ESS and AIES). The ACT graphs and Geweke’s indices plotted in Figures S4–S21 of the Supporting Information indicate that all samplers were run for a sufficient number of samples and have converged. Figure 3 shows representative corner plot outputs for selected highly characterized properties and parameters for one sampler of each class: URS, MH, and ESS. Additionally, we include the corner plot for AIES to show that the posterior found by AIES matches ESS very well for thesehighly characterized parameters. The plots for all parameters and properties are shown in Figures S22–S31 in Section 2 of the Supporting Information. Here, circular scatter plots are indicative of low correlation, while diagonal elliptical graphs suggest a high correlation between the two variables. In general, the correlations among the parameters and properties identified by all of the samplers are in agreement, although some differences exist. The main source of these differences is that MCMC samplers will better sample according to the property uncertainties, relative to a non-MCMC sampler because their sampling is dynamically influenced by the posterior.

#### 3.3.1 Posterior Distributions

The posterior distributions represent the solutions for the parameters after BUQ. The goal is to obtain an accurate representation of the highest posterior density region, which represents the high probability solutions. All of the samplers are exploring the same posterior distribution for the parameter values, and all of the samplers would return a complete and accurate representation of the posterior distribution with infinite time. However, with finite real-world time, their performance can converge to accurate representations of the highest posterior density region with very different time performances, with advanced samplers using superior algorithms that tend to return accurate representations with orders of time faster performance. The MH, ESS, AIES, and URS algorithms showed distinct calculated posterior distributions for most of the parameters. The GS exploration was not suitable for exploration of this high-dimensional complex parameter space, providing a vastly sparse sampling of the highest posterior density region (Figures S28 and S29 of the Supporting Information). ESS and AIES produced similar distributions that were nearly identical for many properties and parameters (Comparison of Figure 3c,d; Figures S26 and S27 and Figures S30 and S31 of the Supporting Information). The fact that ESS and AIES produced distributions that are nearly identical to the eye for most parameters and properties, despite these samplers using very different algorithms, is one sign that they have explored the posterior well and extensively. Finding such a close solution from two different algorithms is one sign of convergence. The narrow distributions depicted in the ESS and AIES samplers demonstrate a better robustness of the developed FF parameters. Moreover, the parameter space predicted by posterior distributions obtained with ESS and AIES was smooth, whereas those derived from MH were rougher as seen by multiple sharp peaks in the posterior distributions (Figure 3). Interestingly, both ESS and AIES found a posterior distribution with two modes (two solutions) where the second mode (Mode 2) was not detected by MH, URS, or GS. This suggests that the MH sampler is less adept at exploring the high-dimensional parameter space. The first mode (Mode 1) was detected by all samplers. The existence of two modes also means that there are two separate local maximum a posteriori probability (MAP) values, which are highest probability points for the solutions.

#### 3.3.2 Insights into Complex Relationship between EAM Parameters and Properties

The ESS results in Figure 3 show that both Mode 1 and Mode 2 have positive correlations between η and *E*_coh_, and both modes have a negative correlation between η and *B*. For both modes, there is essentially no correlation between ρ_e_ and *E*_coh_. For a higher value of ρ_e_, since the embedding energy contours are larger in size, only a minimal contribution of energy is added by the *F*_e_ term, (4th term in Eq. SE4 of the Supporting Information^76^) in the embedding energy, also denoted by increased by η. Consequently, the tendency of atoms to move toward lower electron-density regions results in atoms moving away from each other, which is supported by the corresponding decrease in the attraction term *B*, resulting in an increased lattice constant and reduced *E*_coh_, potentially explaining the correlation between lower ρ_e_ and higher *B* values associated with lower *E*_coh_.

For both ESS and AIES, Mode 1 had almost no correlation between C44 and ρ_e_, whereas Mode 2 had a strong correlation between C44 and ρ_e_. This observation indicates that the two modes are not simply different local optima that are qualitatively similar: rather, that the two modes have distinctly different physical behavior, which explains their ability to each better reproduce different target properties. Mechanical properties, including elastic constants, were accurately predicted by Mode 1 (errors ∼5 to 15%), while Mode 2 showed only slight deviations from the density and surface tension target values (errors from 0.3 to 4.8%). This ultimately underscores the significance of sampling algorithms in obtaining explorations that are reflective of the true posterior space and highlights that BUQ in MD simulations should use samplers capable of exploring complex posteriors that may comprise complexity greater than that of a single continuous mode. A description of the physical relationship between parameters and properties is provided in Section S3 of the Supporting Information.

#### 3.3.3 Validation of GPR Predictions

Overall, the ESS and AIES samplers had the most robust posterior distributions, followed by the MH, with URS and GS samplers being the least robust. The ESS and AIES posterior distributions were characterized by well-resolved peaks, lower variance, higher relative magnitude, and good alignment with experimental values. To validate GPR prediction values obtained for the parameters from posterior distributions, we further performed MD simulations for randomly selected 100 FF parameter sets from the high posterior density region (Figures S32–S41 of the Supporting Information). A comparison between the normal distribution of observed targets (experimental/DFT) with the MD results and the 95% credible interval around the mean of all samples (μ_AP_) is reported in Figure S53 of the Supporting Information. For all properties (except C44 and Poisson ratio), the posterior distribution is overlaid on the experimental target distribution for ESS, demonstrating its capability of finding good solutions in this high-dimensional space (Figures S42–S52 of the Supporting Information). In general, the tradeoff between embedding energy and the pairwise term of EAM benefited either the modeling of mechanical properties or physical properties like density and surface tension. We have tabulated a comparison of values that define the 95% credible intervals (mean and 2 standard deviations) estimated by all samplers of BUQ in Tables S6–S12 of the Supporting Information. Finally, to provide an estimate of the computational resources utilized in this study, we have detailed in Table S13 the number of samples evaluated using PSO with MD simulations and BUQ with this GPR model, expressed in terms of core-hours.

#### 3.3.4 Posterior Coverage Probabilities

**Figure 4:**
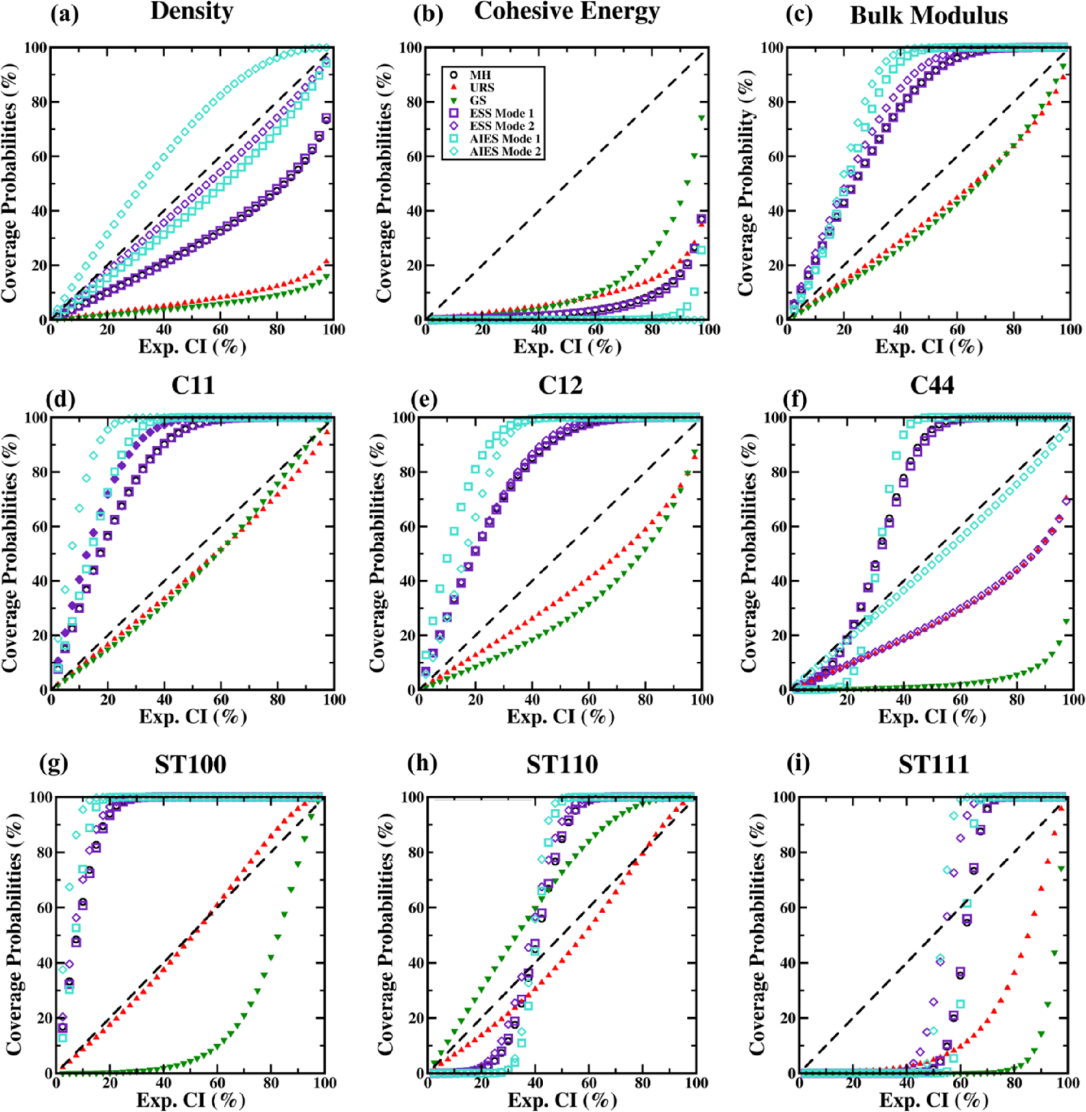
Coverage probability based on posterior presence within likelihood confidence intervals for (a) density, (b) *E*_coh_, (c) bulk modulus, (d) C11, (e) C12, (f) C44, (g) ST100, (h) ST110, and (i) ST111 properties.

The coverage probability is the probability of the true value being within a specific confidence interval. Calculating the coverage probability relative to the likelihood’s confidence intervals is possible using the posterior distributions, since the posterior distribution reflects a probability distribution for the true value.This calculation provides a check of how well the final models match the measured observable. For the FF parameters, a True-Tightened model would be the best case, followed by True-Unchanged, followed by True-Loosened. Figure 4 shows confidence interval coverage probability plots that assess the extent to which the highest density region of the posterior is aligned with (and contained within) the likelihood. In these plots, the coverage probability was calculated with the posteriors approximated as Gaussian distributions centered around their posterior mean values. These plots illustrate the (approximated) percent of a property’s posterior distribution within a given confidence interval from the likelihood. Thus, values that are above the unity line indicate that the percentage of the posterior distribution within that confidence interval of the likelihood is greater than what the confidence value was prior to the analysis. That is, points above the unity line are when that amount of posterior distribution is completely within the estimated uncertainties of the experimental/theoretical expectations. When points are greater than or equal to the unity line in the early portion of the graph (left portion), this indicates that the means of the posterior distribution outputs and the likelihood are in agreement. When the plotted points asymptotically approach 100%, that means that the posterior distribution outputs are fully contained within the likelihood distribution (within rounding). This results when the posterior distribution of the property of interest has a reduction in the standard deviation, and the posterior mean is close to the target property value. In layperson’s terms, if the points reach 100%, then the final models have outputs within experimental/theoretical error. In most cases shown in Figure 4, the posterior distribution is narrower than the likelihood and it is thus possible for the posterior distribution to be fully contained in the likelihood distribution—this situation is not unusual in Bayesian parameter estimation, indicating an addition of information about the appropriate parameters given the experimental data. Accordingly, we do see that many of the coverage probabilities rise to 100% rapidly, which is effectively an indication that the posterior distribution is in agreement with the expected value from the likelihood and also indicates a post-analysis narrowing of the credible interval to be smaller than the pre-analysis likelihood. Thus, our understanding of what the true value is becomes more accurate as a result of considering the joint probability of the MD simulations and the experimental/theoretical values. This can be seen, for example, by comparing Figure 4c and Figure S46 in the Supporting Information. In Figure 4c, the better samplers of AIES and ESS rapidly rise toward 100%, indicating a good match with the likelihood and also a narrower posterior distribution.

Within Figure 4, for any given property panel: if a series rises rapidly to 100%, then that solution corresponds to a True-Tightened FF for that property (such as the AIES and ESS solutions for ST100). If a series falls near the dashed unity line, it is a True-Unchanged FF for that property (such as all of the AIES and ESS solutions for density). If a series does not reach 100% within the graph, then it is a True-Loosened FF (or even False-Loosened FF, which would indicate no match between the posterior and experimental mean, as well as broader uncertainty) for that property (such as the URS and GS solutions for density). In general, the results from the ESS and AIES samplers were more likely to rise rapidly and to approach 100% coverage probability (for the specific mode that better predicts that property). This indicated a narrowing of the posterior relative to the original confidence interval while staying within it, suggesting an improved precision in the estimate of the true value. Furthermore, this rise to 100% shows that the models are robust, as the parameters can be adjusted within the credible intervals of the posterior distribution without exiting the confidence intervals obtained from the target experimental/theoretical values and their uncertainties for those properties. AIES and ESS outperformed the other samplers in finding True-Tightened FF for more properties. MH was the median sampler: it tended to be between the best samplers and the worst samplers. With all BUQ samplers, there is a balance in trading to improve the performance of predicting some properties at the expense of other properties. The AIES and ESS samplers were the best samplers in this study as they provided more True-Tightening of properties and were the only samplers to find the two modes of solutions due to their efficient exploration of the high-dimensional FF parameter space. As further depicted in Figure S46, the posterior distributions from AIES and ESS are in agreement with the observed value while also providing a narrower credible interval. AIES and ESS provide qualitatively similar posterior distributions considering both modes separately.

As mentioned previously, there is a tradeoff between the two modes by the accuracy of density predictions with the elastic properties, with mode 1 emphasizing elastic property accuracy and mode 2 emphasizing density property accuracy. For mode 1, AIES shows a quicker convergence to full coverage probability over ESS as exemplified in all elastic constants while maintaining a coverage probability close to unity for density as ESS shows a slow increase in coverage probability for density. In mode 2, AIES maintains coverage probabilities above the unity line for density and at the unity line for C44, while ESS has coverage probabilities at the unity line for density and significantly below the unity line for C44. The AIES sampling here performed better for these properties. It is important to recognize that this narrower credible interval is based on the Bayesian parameter estimation considering the joint probabilities of the MD outputs of *all* properties. Thus, the physical basis of MD, and the knowledge that “a completely physically correct simulation would correctly produce all properties”, enables the Bayesian parameter estimation to create a posterior distribution with narrower credible intervals relative to the experimental observations’ uncertainties. In addition to providing an improved estimate of the true value, such posterior distributions also enable creation of more robust MD models: because they indicate that varying the parameters within the range of the posterior mode will not exit the confidence intervals obtained from experiment/theoretical values.

In contrast to the advanced samplers, the URS sampler tends to more often follow or be below the unity line due to less refinement of the model than the more advanced samplers, indicating no additional information about the parameters was added by Bayesian parameter estimation. In some cases, such as with the density outputs from URS, the coverage probability never reaches 100%, as the posterior distribution is not fully contained in the likelihood distribution. This behavior arises when the posterior distribution of the property remains broader than the experimental uncertainty and/or the posterior mean deviates from the target property value. Another interesting case in Figure 4 occurs where the coverage probability shows an “S-shaped” behavior, which results from a narrowing of the posterior distribution with a posterior mean that deviates from the target property value. The coverage probability remains low and then rapidly increases to full coverage after the experimental confidence interval includes the posterior mean value. In total, the various coverage probability graphs show that the advanced samplers create final outputs that are in better agreement with the experimental observations. These graphs thus further reinforce the importance of using advanced samplers. The better performance of the advanced samplers is due to their ability to find and hone in on the highest posterior density regions and thus result in posterior distributions that are more likely to be contained within the likelihood.

Both AIES and ESS are very effective algorithms for exploring high-dimensional posteriors. Neither sampler can be said to be superior for all possible posteriors, particularly given that both algorithms involve hyperparameters. In the general case, because AIES involves jumps and ESS involves slices, we would anticipate that AIES would perform better than ESS for some cases where the posterior has much more roughness at the fine scale or has discontinuities. In contrast, we would anticipate that ESS would perform better than AIES for cases where there are only a small number of modes and there is a continuous posterior distribution. One advantage of ESS is that it is rejection free, which gives it a speed advantage in exploring smooth posteriors.^78^ To overcome the difficulty that ESS is known to have for traversing low or no probability regions, there is an ESS variation in which global moves (a type of jump) are introduced into ESS. The global moves option is available in the ESS package used during this study,^78^ but was not used in this study as ESS using global moves is not a good general choice for an unexplored posterior (based on our own experience and also the documentation of the software package). In this study, the performances of AIES and ESS were similar, with ESS being slightly faster and resulting in a slightly more converged posterior. This is consistent with the expected behavior, as the posterior distributions in this study are relatively continuous and smooth, such that the performance gap was not solution-determining between these two algorithms for this study. It is important to note that, when there are multiple modes, the shape of the posterior and the separation between the modes may play a factor in determining which sampler would find all modes sooner. Thus, we recommend considering using both samplers if possible when investigating complex high-dimensional problems, such as in this study. The solutions provided by each can then be examined.

## 4 Conclusions

In conclusion, the newly developed MCMC algorithms ESS and AIES are advantageous in achieving True-Tightened FFs for more robust UQ, when compared to the MH, URS, and GS algorithms, for high-dimensional and complex parameter spaces. Notably, for the FF parameter UQ estimation problem examined here, both ESS and AIES were capable of detecting two separate posterior modes of solutions, a nuance that all other algorithms failed to capture. ESS and AIES thereby not only found improved solutions that were missed by the traditionally used samplers, but also sensitivity analyses of these modes provided additional understanding of the relationships between parameters and properties. Thus, applying these newly developed MCMC samplers to complex model development, with comparison to experimental and theoretical values, enhances physically realistic models while also deepening the physical understanding of MD FF parameters. These results are likely applicable across various experimental and computational subfields of materials science. Therefore, our research highlights the critical need and urgency to evaluate and compare MCMC sampling algorithms in diverse areas of materials science and engineering, especially considering the increasing interest in BUQ methods.

The BUQ samplers employed in this study serve as tools for executing the UQ of any given system. This framework is not limited to MD and can be adapted to any computational model that takes inputs from a parameter space to produce numerical outputs. The use of GPR surrogates during BUQ was for acceleration and is not a crucial step in the framework. Other surrogate types with uncertainty propagation could be used instead, and computational models that are sufficiently computationally cheap may be used directly without a surrogate. The framework used here is general enough to apply to FFs for all-atom and coarse-grained systems, including organic molecules, glycomaterials, biomolecules, polymers, and systems with solvation. When used in concert with experimental researchers, this framework can enable the scientific community to make more informed models and also inform experimental design.

## Data Availability

The data sets generated and/or analyzed during the current study are available on our GitHub:: https://github.com/Deshmukh-Group/BUQ_all-atom_gold.git.

## Supporting Information Available

Detailed methodology of Particle Swarm Optimization (PSO) and Embedded Atom Method (EAM) potentials for modeling metals; results: convergence of Bayesian samplers and posterior distribution corner plots for all parameters and properties; and physical insights of bimodal distribution (PDF)

## Notes

Sample calculations, analysis codes, and detailed explanations of the code used in this paper are available on GitHub: https://github.com/Deshmukh-Group/BUQ_all-atom_gold.git. PEUQSE code is available at https://github.com/AdityaSavara/PEUQSE.
