# Supplementary material for: Evaluation of Sampling Algorithms Used for Bayesian Uncertainty Quantification of Molecular Dynamics Force Fields
Source: J Chem Theory Comput. 2024 Jun 26;20(13):5732–42. doi: 10.1021/acs.jctc.4c00130 (PMC11238537; doi:10.1021/acs.jctc.4c00130)
Supplement: Supplementary file 1 — ct4c00130_si_001.pdf [file ct4c00130_si_001.pdf]

Supporting Information

Evaluation of Sampling Algorithms used for Bayesian Uncertainty Quantification of Molecular Dynamics Force Fields

Abhishek T. Sose[1#], Troy Gustke[1#], Fangxi Wang[1], Gaurav Anand[1], Sanjana Pasupuleti[1], Aditya Savara[2*], and Sanket A. Deshmukh[1*]

[1]Department of Chemical Engineering, Virginia Tech, Blacksburg, VA, 24060, USA

[2]Oak Ridge National Laboratory, Oak Ridge, Tennessee 37830, United States

[#]Both authors contributed equally

***Corresponding Authors:** Aditya Savara (savaraa@ornl.gov), Sanket A. Deshmukh (sanketad@vt.edu)

Table of Contents:

Section S1. Methodology and Models……………………………………………...**2–18**

I) EAM FF Parameter Development………………………………2

II) Bayesian Uncertainty Quantification……………………………10

Section S2. Results and Discussion……………...………...……….……………**19-72**

I) Convergence Metrics of Bayesian Samplers………………………19

II) Corner Plots…………………………………………………...39

III) Credible Intervals……………………………………………..49

IV) Bayesian UQ Posterior Analysis…………………………………55

Section S3. Physical Insights……………………………………………….....**73-74**

Section S4. References…………………….………………………………....**75-76**

Section S1) Methodology and Models

I) EAM FF parameter Development:

(a) Embedded Atom Method (EAM) Potentials:

In MD simulations, the embedded atom method (EAM) potential accounts due to its high accuracy resulting from quantum mechanics modeling and simplistic mathematical formalism, has been employed in large-scale computer modeling. In EAM, the total energy of a system is determined

$$E_{total} = \sum_{i=1}^{N} F_i(\rho_i) + \sum_{i=1}^{N} \sum_{j>i} \phi_{ij} R_{ij} \quad \textbf{…….. Eq. (SE1)}$$

F_i is the embedding energies, which is the energy required to embed an atom i in the host electronic density (ρ_i) of the surrounding atoms, and the pairwise interaction energies (ϕ_{ij}) at interatomic distance R_{ij} between atoms i and j.[1]

$$\rho_i = \sum_{i \neq j}^{N} f_j (R_{ij}) \quad \textbf{…….. Eq. (SE2)}$$

Here, f_j denotes the density of atom j, which is situated at a distance R_{ij} from atom i. The second term in **Equation (SE1)** accounts for the pairwise interaction energies Φ_{ij} existing between atoms i and j.

$$\Phi_{ij} = \frac{A\, exp(-\alpha[(R_{ij}/r_e)-1])}{1+[(R_{ij}/r_e)-\kappa]20} - \frac{B\, exp(-\beta[(R_{ij}/r_e)-1])}{1+[(R_{ij}/r_e)-\lambda]20} \quad \textbf{…….. Eq. (SE3)}$$

Here, r_e represents the equilibrium spacing between nearest neighbors. The parameters A, B, α, and β are adjustable coefficients, while κ and λ are cut-off parameters used to adjust the distance at which the pairwise interaction between two atoms becomes negligible. Moreover, to comprehensively model the embedding energy across a wide density range, Johnson et al. proposed an approach involving the consideration of distinct density regions, each characterized by its functional. Specifically, for the density intervals [0, $0.85\rho_e$] and [$0.85\rho_e$, $1.15\rho_e$], they employed cubic splines with varying coefficients, where ρ_e represents the equilibrium electron density *i.e.* the point at which the embedding energy reaches its minimum value. In the asymptotic region with densities $\rho_i > 1.15\rho_e$, an asymptotic power-logarithm functional form was chosen to generate a repulsive embedding energy to handle high-density scenarios. While this functional form of the EAM (Embedded Atom Method) potential has proven accurate for describing surfaces and bulk systems, it may not provide optimal precision for low-density regions, such as atoms situated at low-coordinated sites on the edges and corners of nanoparticles. To address such

situations, Shan et al. proposed an alternative approach involving the use of another cubic spline function to model the low-density region, represented by the interval [0, ρ_l], where ρ_l serves as an adjustable parameter. In summary, the employed functional form of the embedding energy in the current study as proposed in Marchal *et al.* is:

$$F_i(\rho_i) = \begin{cases} \sum_{k=0}^{3} F_{mk}\left(\frac{\rho_i}{\rho_l}-1\right)^k & \text{for } 0 \le \rho_i < \rho_l;\ \rho_l = \gamma\rho_e \\ \sum_{k=0}^{3} F_{nk}\left(\frac{\rho_i}{\rho_n}-1\right)^k & \text{for } \rho_l \le \rho_i < \rho_n;\ \rho_n = 0.85\rho_e \\ \sum_{k=0}^{3} F_{k}\left(\frac{\rho_i}{\rho_e}-1\right)^k & \text{for } \rho_n \le \rho_i < \rho_0;\ \rho_0 = 1.15\rho_e \\ F_e\left[1-\eta\ln\left(\frac{\rho_i}{\rho_e}\right)\right]\left(\frac{\rho_i}{\rho_e}\right)^\eta & \text{for } \rho_i \ge \rho_0 \end{cases}$$

…….. Eq. (SE4)

We utilized the EAM potential formulation proposed by Johnson et al. to run MD simulations to predict physical, structural, mechanical and thermodynamic properties of the FCC metal *viz.* gold (Au) [2–4]. To optimize parameters fitting towards 10 properties of interest involved with gold, i.e. E_{coh}, density , bulk modulus, C11, C12, C44, poisson ratio, and surface tension for (100), (110), and (111) surfaces, we employed particle swarm optimization (PSO) integrated with MD simulations through LAMMPS[5]. Note, the MD simulations for density were run for 50 ps, with timestep of 1 fs, while elastic constants and cohesive energy were calculated by minimizing/relaxing the system with 20,000 maximum iterations and 200,000 maximum evaluations with stopping criteria for both energy and force at $1*10^{25}$. Moreover, the cohesive energy was calculated first and then used in the subsequent calculations wherever required. In the case of surface tension/energy and vacancy formation energies calculations, these numbers were {max_iterations: 10,000, max_evaluations: 100,000, with stopping criteria for both energy and force at $1*10^{15}$}. All the simulations for density calculations were calculated at 298.15 K. All these properties are listed in **Table S1**

Bayesian parameter estimation requires an uncertainty for each property to create an objective function that is fully based on probability. As mentioned in the main text in Eq. 2, the target property is augmented by an error term which is assumed to be a normal distribution. This error term is from the sum of experimental uncertainty and uncertainty in the surrogate model (ie. GPR model predicted uncertainty). The experimental uncertainty is determined by taking the standard deviation of experimental values found through a literature review. The surrogate model uncertainty is predicted along with the property prediction. The experimental uncertainties are shown below in Table S1 along with experimental values used for the calculation.

Table S1. Experimental target values and uncertainties for each property.

**Property [Units]**	**Target**	**Uncertainty**	**CoV (in %)**
Cohesive Energy [eV/atom]	-3.81	0.057	1.496
Bulk Modulus [GPa]	173	5.53	3.197
C11 [GPa]	192	7.877	4.103
C12 [GPa]	163	5.41	3.319
C44 [GPa]	42.3	1.468	3.47
Poisson Ratio	0.42	0.0034	0.81
Surface Tension (100) [mJ/m3]	1540	60.1	3.903
Surface Tension (110) [mJ/m3]	1600	60.1	3.756
Surface Tension (111) [mJ/m3]	1480	60.8	4.108
Density at 298.15 K [g/cm3]	19.3	0.059	0.306
Vacancy Formation Energy [eV/atom]	1.022	0.07	6.849

(b) Particle Swarm Optimization:

Kennedy and Eberhart first proposed the PSO method in the 1970s, and it is derived from the way a flock of birds searches for food. In an iterative process, it attempts to enhance the position of a swarm of candidate solutions (particles) within the search space. There are different types of particles that represent different parameters or variables. Typically, a PSO run uses more particles in order to cover a wider parameter space and identify optimal solutions more quickly. In order to avoid computational burdens, however, a balance must be struck between using too many particles and using too few. In order to optimize the parameters, initially, each particle is assigned random positions (parameters to be optimized) and velocities.

Fitness is determined by the degree to which the simulated properties of each particle align with the desired target properties, based on the molecular dynamics simulations of each particle. There is a personal best for each particle (p_{best}), which represents where it has achieved the best fitness so far. Further, the particle with the best fitness over all completed PSO runs becomes the global best (g_{best}). Using formulae given below, gbest and pbest influence new particle positions (x_{n+1}) and velocity (V_{n+1}).

$$V_{n+1} = w*V_n + c1*rand()*(g_{best} - x_n) + c2*rand()*(p_{best} - x_n)$$ **........ Eq. (SE5)**

$$x_{n+1} = x_n + V_{n+1} * \Delta t$$ **........ Eq. (SE6)**

The global and personal constants c1 and c2 are used to calculate g_{best} and p_{best} contributions to the new velocity. In order to avoid premature convergence, the inertia factor (w) induces inertia from the original velocity. Throughout each iteration, the PSO continues to update particle positions and velocities, and g_{best} and p_{best} values are updated until at least one particle achieves a fitness lower than the tolerance, representing the optimal set of input parameters.

In this study, we have used 128 particles (birds) to explore 14-dimensional space with the bounds for each parameter defined in **Table S1**. To obtain the desired target properties, 100 total number of PSO epochs were run.

Table S2: The table of range of values for all 14 parameters with the final optimized set.

	**Range for optimization**

	Min	Max	Final optimized parameter
**rhoe**	11.537	14.485	**12.40053**
**alpha**	8.03	11.5	**9.319661**
**beta**	3.801	6.0	**4.266695**
**A**	0.15	0.22	**0.22**
**B**	0.306	0.9	**0.567516**
**lamda**	0.603	1.5	**1.01605**
**kappa**	0.34	1.863	**0.562943**
**Fn3**	-3.2	-1.107	**-2.062738**
**F2**	1.405	1.897	**1.693363**
**Fe**	-2.6	-0.804	**-1.419297**
**etha**	1.107	1.7	**1.394784**
**rhom**	0.659	0.9	**0.747859**
**lattice_constant**	4.04	4.1	**4.052219**
**fe**	1.1	1.8	**1.260406**

(c) Property Calculations:

The methodology of calculations and target property values are mentioned in the following section.

***(i)* Cohesive energy**: The cohesive energy is the energy required to separate constituent atoms apart from each other from the state of assembly. In this case, potential energy per atom is calculated as per following commands in lammps:

"compute eng all pe/atom

compute eatoms all reduce sum c_eng"

The total energy of assembly (eatoms) is divided by the total number of atoms (n_atoms) to compute the cohesive energy.

***(ii)* Density:** In this study, the cell parameter i.e. lattice constant was allowed to vary to achieve equilibrium density through NPT molecular dynamics simulation under standard conditions (1 bar and 298.15 K). A Nose-Hoover thermostat with 1.0 ps and barostat with 20.0 ps coupling constants, with a timestep of 1 fs was run to equilibrate the system within a total of 50 ps.

***(iii)* Surface Tension:** The models of {100} and {111} surfaces were constructed with the dimensions l_x, l_y: 8 x L_c, 8 x L_c (L_c: Lattice constant). While in the z-direction, the box length spanned to 16 x L_c, whereas atoms comprised only in length, 12 x L_c. The free space (vacuum slab) generated by removing atoms in the length of 4 x L_c, was sufficient to estimate the surface energy of the two separated surfaces. The total energy of the separated system after minimization was computed as E_s. And the energy of the unified surfaces, E_u, was determined by the cohesive energy times the total number of atoms. The surface tension was calculated using:

$$\gamma \approx \frac{Es - Eu}{2A} \text{ …….. } \textbf{Eq. (SE7)}$$

Where, the denominator comprises of total surface area, *i.e.*, 2 x A = 2 x l_x x l_y. The surface tensions for 100 and 111 surfaces were only considered because of the availability of reliable experimental data to optimize for.

***(iv)* Elastic constants:** The isotropic elastic constants i.e., Poisson ratio, C11, C12, shear modulus (C44), and bulk modulus were computed by following Hooke's law where the bulk system was

displaced in 1 direction (x, y, or z) and the consequent stress and strains in all three directions were determined. Following the calculation of stress-strain tensor, only three stiffness constants i.e. C11, C12, and C44 were computed and using these constants, bulk modulus and poisson ratio were determined as in the following equations Eq. SE8 and SE9:

$$\text{Bulk modulus} = \frac{(C11+C22+C33) + (C12+C21) + (C13+C31) + (C23+C32)}{9} \quad \textbf{........ Eq. (SE8)}$$

$$\text{Poisson Ratio} = \frac{(C12+C21) + (C13+C31) + (C23+C32)}{2*(C11+C22+C33) + (C12+C21) + (C13+C31) + (C23+C32)} \quad \textbf{........ Eq. (SE9)}$$

The parameterized EAM yielded excellent accurate results reproducing the experimental target properties including bulk, surface and elastic properties, and their corresponding errors are shown in **Table 1** of main article and **Table S2**. In general, the primary properties of metals i.e. cohesive energy, lattice constant and density were well within ~15 % of the targets.

Table S3 - Property results from the newly developed AA EAM model compared to Experimental observations.

**Properties**	**Units**	**Gold EAM model developed in this study**	**Experimental/ DFT (Target values)**	**Experimental Uncertainty**	**Absolute Percent error between experiment /DFT data and EAM predictions**
Cohesive Energy (E_{coh})	eV/atom	-3.853	-3.81[6]	0.057	1.13
Bulk modulus	GPa	171.026	173[7]	5.53	1.14
C11	GPa	188.073	192[8]	7.877	2.04
C12	GPa	162.502	163[8]	5.41	0.31
C44	GPa	43.337	42.3[8]	1.468	2.45

Poisson ratio	N/A	0.464	0.42[9]	0.0034	10.47
Surface Tension (100)	mJ/m^2	1377.107	1540[10]	60.1	10.57
Surface Tension (110)	mJ/m^2	1524.321	1600	60.1	4.73
Surface Tension (111)	mJ/m^2	1250.786	1480	60.8	15.49
Density at 298.15 K	g/cc	19.018	19.3[11,12]	0.059	1.46

Table S4: The comparison of elastic, surface and structural properties of gold obtained in this study with the current literature properties.

**Properties**	**Weights for PSO opimization (%)**	**Targets/Experimental Values**	**This work**	**HyBOP**[13]	**Foiles et al.**[14]	**Alvi et al.**[15]	**Ackland et al.**[16]	**Zhou et al.**[17,18]	**ReaxFF**
Cohesive Energy	12.82	-3.81	-3.85	-3.82	-3.93	-3.93	-3.79	-3.93	-3.77
Poisson Ratio	10.26	0.42	0.46	-	-	-	-	-	-
C11	10.26	192	188.48	231	183	179.95	187.54	190.72	201
C12	10.26	163	162.92	170	159	158.64	157.75	157.36	151
C44	10.26	42.3	43.33	75	45	34.842	43.32	44.93	55
Bulk Modulus	10.26	173	171.44	-	-	-	-	-	-
ST 100	6.41	1540	1377.12	-	-	-	-	-	-

ST 110	6.41	1600	1524.09	-	-	-	-	-	-
ST 111	10.26	1480	1451.44	-	-	-	-	-	-
Density	12.82	19.3	19.02	-	-	-	19.29	19.26	-

II) Bayesian Uncertainty Quantification

Uncertainty quantification (UQ) plays a pivotal role in computational modeling, enabling the comprehensive understanding of uncertainties and assessment of model reliability. UQ has been employed in various fields, such as climate modeling,[19] materials design[20], and drug development[21,22]. UQ provides tangible benefits by allowing researchers to quantify credible intervals for their models, to prioritize areas for model refinement, and to make informed decisions based on the known limitations and strengths of their models. Remarkably, While certain studies have delved into the quantification of uncertainties in model outputs using various techniques[23], the task of fully encapsulating the complexities and uncertainties inherent to model parameters remains a largely uncharted territory. The exploration of this parameter uncertainty landscape is crucial for a comprehensive understanding of the reliability and robustness of computational models across various disciplines.

An accurate uncertainty quantification relies on the choice of an appropriate sampling algorithm, which aims to efficiently explore the parameter space and approximate the posterior distribution of interest. Hence, in this study, as our exploration into computational modeling progresses, specifically focusing on molecular dynamics, we gained a greater understanding and recognition of the significance of these advanced samplers.

A) Sobol Sensitivity Data

The calculations for sobol sensitivity are shown in the main manuscript.

Coefficient of variation (CoV): The coefficient of variation (CoV) is a statistical measure that describes the relative variability in a data set. It is calculated as the ratio of the standard deviation (σ) to the mean (μ), expressed as a percentage, providing a normalized measure of the dispersion of properties.

$$CoV(\%) = \frac{\sigma}{\mu} \times 100 \text{ …….. } \textbf{Eq. (SE10)}$$

The CoV for most of the properties were approximately ~5 %, with C44 at ~7 %, while the poisson ratio and cohesive energy (E_{coh}) exhibit the lowest variations of 0.44 % and 1.8 %, respectively. Moreover, a majority of properties exhibited a root mean squared error (RMSE) of < ~0.055, except for C44 (RMSE = ~0.076), demonstrating the effectiveness and reliability of GPR models.

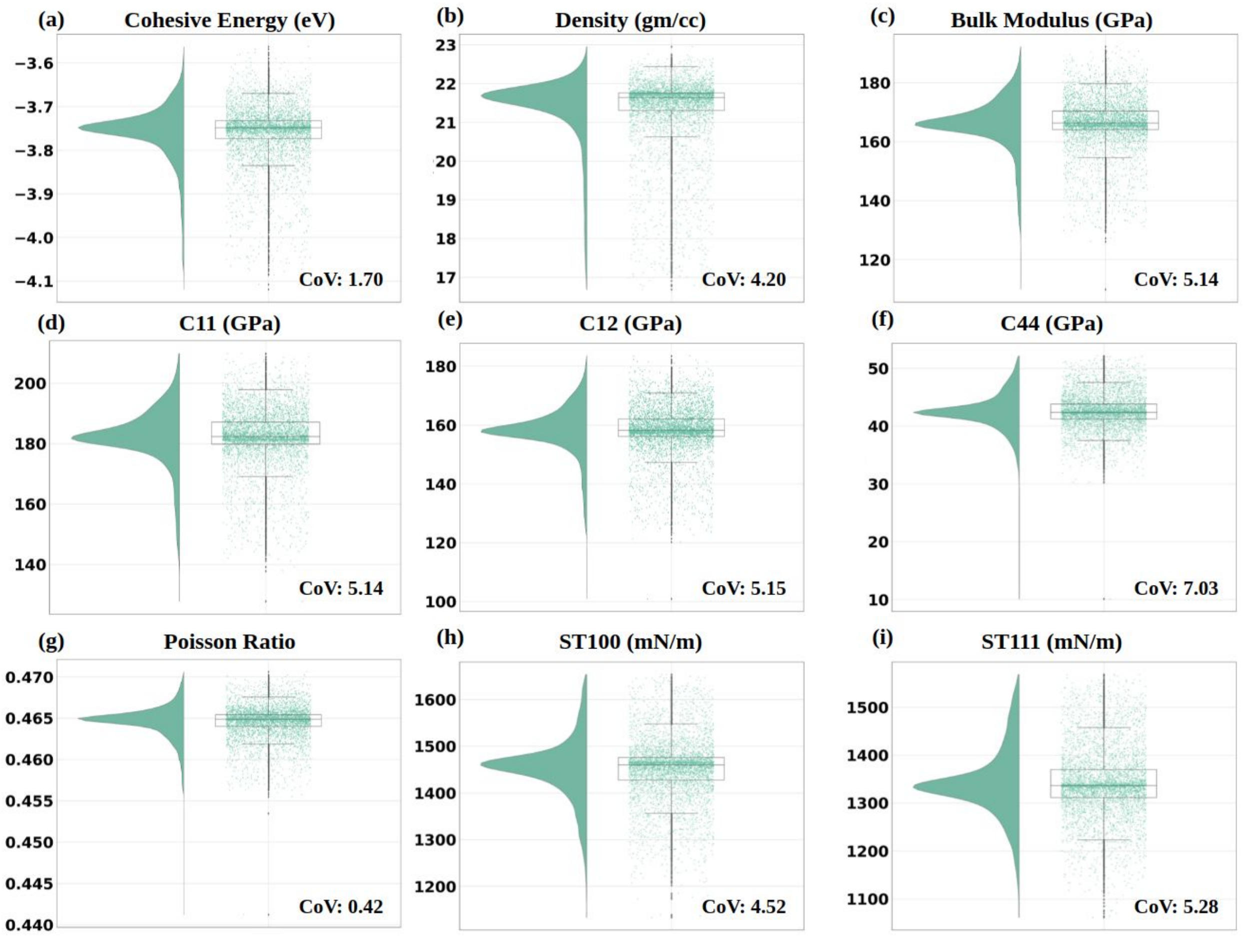

Figure S1: The raincloud plots for each property optimized for in this study.

B) Gaussian Process Regression (GPR):

Due to the computationally expensive nature of MD simulations, surrogate models have been employed to enable faster property predictions. Various surrogate models have been explored in the context of MD, such as artificial neural networks, support vector machines, and kernel ridge

regression, among others. In recent years, Gaussian Process Regression (GPR) has emerged as a promising surrogate model for MD simulations, owing to its flexibility and ability to provide probabilistic predictions. GPR is particularly well-suited for Bayesian uncertainty quantification due to its non-parametric nature, probabilistic predictions, and compatibility with Bayesian inference. This compatibility allows GPR to naturally incorporate prior knowledge and provide a full probabilistic description of the uncertainty in the predictions, making it an ideal candidate for uncertainty quantification in molecular dynamics models using Bayesian approaches.

Since the covariance function (kernel) is important for the prediction behavior of the GPR model, multiple kernels were compared using the k-fold cross validation with 5 folds over 1000 data points. A total of five different kernels were compared, including (1) Radial basis function (RBF), (2) Exponential, (3) Matern 32, (4) Matern 52, and (5) RBF+RBF. These selected kernels are commonly employed because they can effectively model a diverse range of smooth functions, catering to various degrees of smoothness. Some kernels excel at modeling highly smooth functions, while others are more adept at handling rougher solution surfaces, thereby ensuring versatility in accommodating different types of relationships in the data. The kernel with the highest average coefficient of determination was chosen as the covariance function for each property. To ensure efficient training and inference without sacrificing accuracy, the GPR models were trained on a subset of 1000 data points, while the remaining 4000 points were employed as testing data. Finally, the best kernel was chosen and surrogate model was trained on all 5000 points, to be employed further in Bayesian analysis.

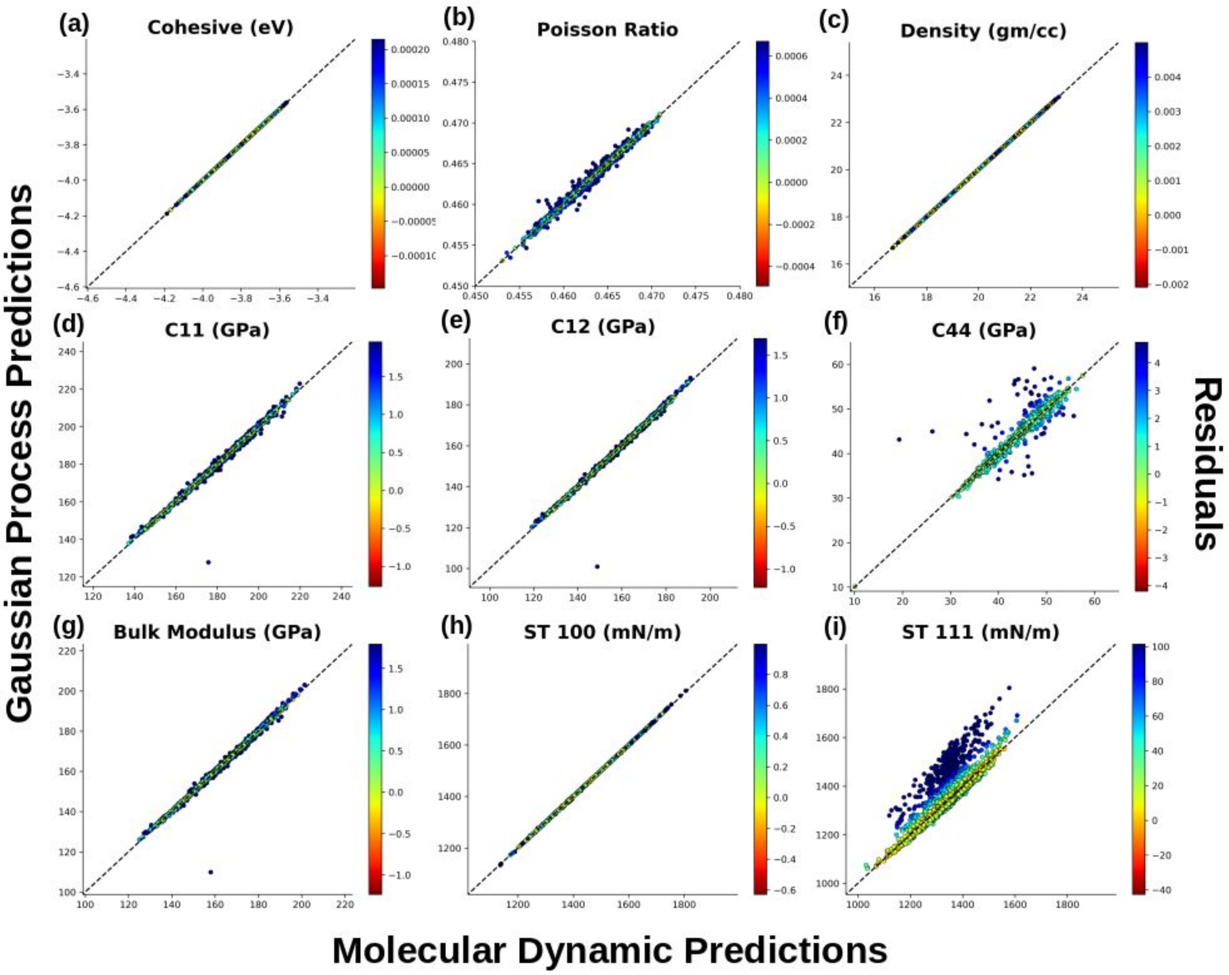

Figure S2: The prediction performance of GPR models for each property.

Table S5. R-Squared, normalized RMSE between GP prediction and MD prediction.

Properties	Covariance Function	R-Squared	STD Normalized RMSE
Cohesive Energy	Matern52	0.999	0.018
Poisson Ratio	RBF+RBF	0.975	0.004
C11	Matern32	0.991	0.053
C12	Matern32	0.991	0.053
C44	Exponential	0.973	0.076
Bulk Modulus	Matern32	0.991	0.053

Surface Tension 100	RBF	0.999	0.048
Surface Tension 110	Matern32	0.925	0.047
Surface Tension 111	RBF+RBF	0.650	0.058
Density	RBF+RBF	0.999	0.042

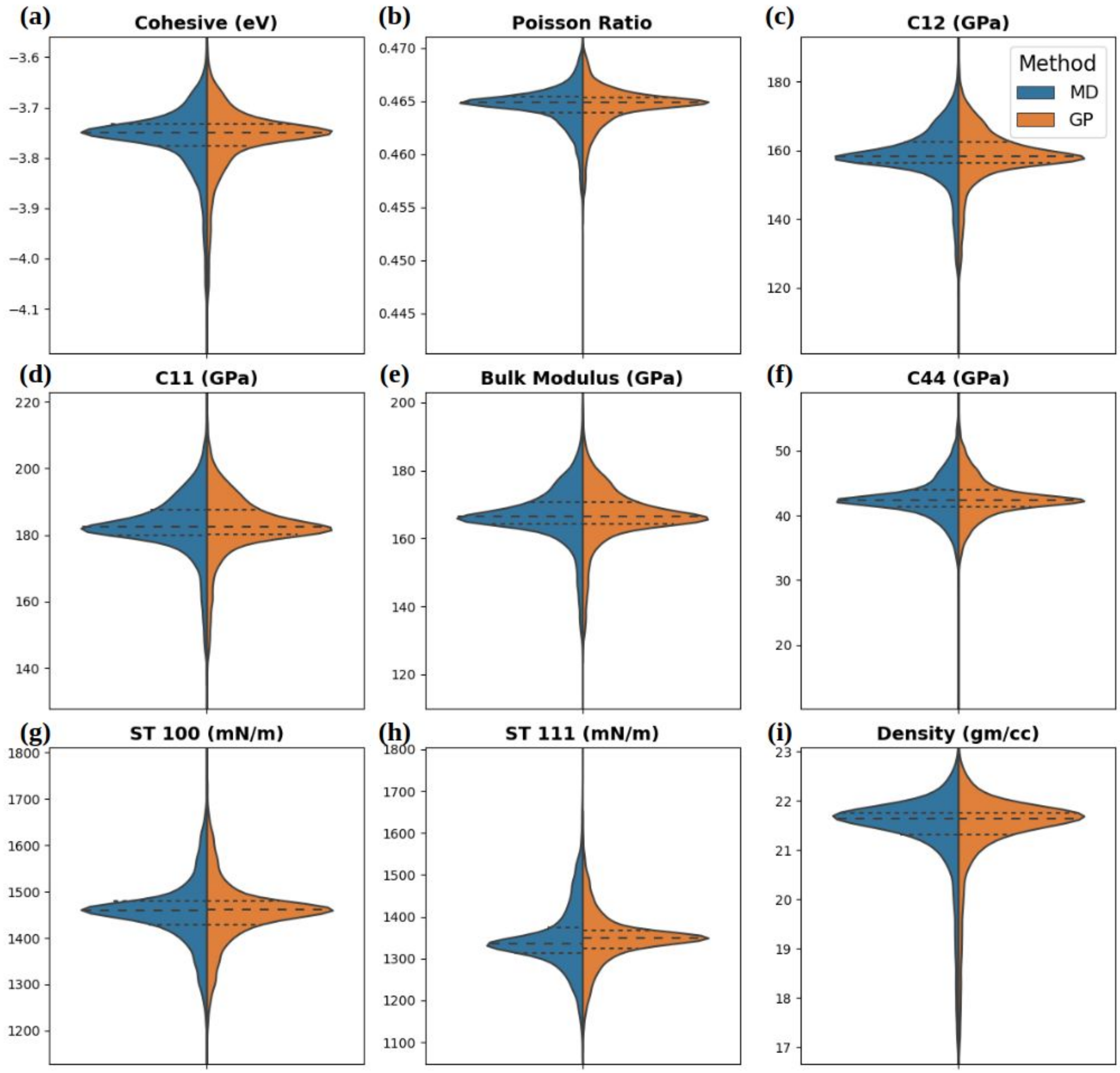

Figure S3. The violin plots represent the distribution of properties in the sobol varied data.

Sobol Sensitivity Indices: Sobol sensitivity indices or sobol measures are the statistical tools to quantify the variance in the output properties with respect to input parameters. The sobol indices can relate higher order interactions to variances in properties as well. To determine the effect of higher-order interactions in the system, a comparison of first order indices and the total order can be performed, where if no changes are observed, then higher order interactions are negligible.

Further, we employed sobol sensitivity indices to investigate the sensitivity of the model . In contrast to the sobol perturbation, sobol sensitivity indices is a variance-based analysis approach that enables us to trace variations in the properties back to alterations in parameters providing a more nuanced understanding of our model. First-order indices relate changes in one parameter to variance in a property directly, while higher-order indices relate variance contributions to interactions of parameters. For example, for a system with two parameters, sobol analysis could show that 80% of the variance contributions is from one parameter and 15% for the second, and the interaction of the two parameters contributes towards 5% of the total variance. The total variance contributions sum to 1, so first-order relations dominate when the sum of the first-order indices is about 1. This is the case for the system of study.

C) Bayesian Analysis

I) Details of sampling algorithms used in this study.

A) MCMC Sampling Algorithms.

1. **Metropolis Hastings (MH):**

   The Metropolis Hastings (MH) algorithm is a Markov Chain Monte Carlo (MCMC) method used to generate samples from complex targeted probability distributions.[24] The MH algorithm overcomes the challenge of seeking high density probability regions and characterizing their distributions, within the posterior distribution. MH does so by constructing a Markov chain that converges to the desired distribution with infinite sampling. This algorithm leverages the concept of a proposal, which represents a possible next sampling point of parameter values to jump to from the current state of the Markov

chain. Based on an acceptance ratio based on the posterior, the proposed sample (next point) is accepted or rejected. The algorithm is stated as follows:

(i) At each step, a set of parameter values is associated with the current location (Let's denote this as θ_i).

(ii) A new parameter is proposed within a certain jump distance. (Let's denote this as θ^*).

(iii) An acceptance ratio (α) is calculated to decide if the proposed value is accepted or rejected, based on the ratio of the posterior probability density at the two points. $\alpha(\theta^*) = P(\theta^*)/P(\theta_0)$.

(iv) A random number generator is used to pick a value u from between 0 and 1. If $u > \alpha$, then no jump occurs. If $u \leq \alpha$, then jump to the proposed point.

2. **Affine-Invariant Ensemble Sampler (AIES)**

The Affine-Invariant Ensemble Sampler is MCMC method that uses an ensemble of Markov chains (walkers) and a stretch move for proposing new points[25,26] to help the sampler effectively explore highly correlated or skewed distributions. In PEUQSE, this feature is referred to as Ensemble Jump Sampling (EJS). The ensemble of walkers allows the sampler to more effectively search the parameter space compared to a single walker. The stretch move for proposing points is an affine transformation (linear transformation followed by a translation), which transforms a set of points while preserving the points' relative positions and angles to each other. This allows the performance of AIES to not depend on the orientation, skewness, or correlation structure of the probability distribution it's sampling.

The algorithm is stated as follows:

(i) Initialize an ensemble of points sampled from the prior distribution.

(ii) Randomly select a walker (walker i) from the ensemble for updating.

(iii) Randomly choose another walker (walker j) from the ensemble such that $i \neq j$.

(iv) Make a proposal for a new position for walker i. The difference between walker j and i is added to the current walker position with a scaling factor, z, as shown in equation X. Z is a random variable drawn from a distribution shown in equation SE16, where a is an adjustable scale parameter.

$$X_i(t)\to X_i(t+1) = X_j + Z[X_i(t) - X_j]$$ **…….. Eq. (SE11)**

$$g(z) \propto \begin{cases} \frac{1}{\sqrt{z}} & \text{if } z \in \left[\frac{1}{a}, a\right] \\ 0 & \text{otherwise} \end{cases}$$ **…….. Eq. (SE12)**

(v) Compute the Metropolis-Hastings acceptance probability for the proposed point. If the acceptance criterion is met, the walker i moves to the new position, but remains at the current position if rejected.

(vi) Iterate steps 2-5 until the ensemble of points represents the posterior distribution.

3. **Ensemble Slice Sampling (ESS)**

Ensemble Slice Sampling is an advanced MCMC method that combines the strengths of ensemble methods[25] and slice sampling[27] to provide an efficient way of sampling from complex, high-dimensional distributions. Ensemble methods use multiple walkers to explore the distribution independently, which allows for a more effective exploration of the parameter space rather than a single walker. Slice sampling is a univariate method that works by picking a random point from the distribution and then "slicing" the distribution at that height. Further points are selected within this slice as an interval is refined to represent the bounds of the univariate distribution. This can be thought of as a rubber band being placed around the distribution and the rubber band tightens as more samples are taken. ESS combines these two ideas to further inform the selection of new points using various "moves". In this work, the differential move is used which draws points along a vector that is made from the current point and a random point in the ensemble.

The algorithm is stated as follows:

(i) Initialize an ensemble of points sampled from the prior distribution.

(ii) Randomly select one point from the ensemble to serve as the current point.

(iii) Choose a direction vector by taking the difference between the current point and another randomly selected point from the ensemble.

(iv) Draw a slice level uniformly between zero and the probability density of the current point in the direction of the chosen vector.

(v) Define an interval along the chosen direction vector which contains points with densities above the slice level. This is done by stepping outwards from the current point until the bracket encompasses the slice.

(vi) Propose a new point uniformly within this bracket.

(vii) If the proposed point is on the slice (i.e., its density is above the slice level), accept it. If not, shrink the bracket to exclude the rejected point and propose a new point within the shrunken bracket.

(viii) Iterate steps 2-7 until the ensemble of points adequately represents the posterior distribution.

B) Non-MCMC Sampling Algorithms

4. **Grid Search**

A grid search sampler divides the parameter space into a grid and samples at the predefined points on the grid. The grid sampler is computationally simple and easy to understand but suffers greatly from the curse of dimensionality. The number of samples is determined by the number of grids raised to the number of dimensions. This ultimately limits the exploration of a high dimensional parameter space and does not accurately represent a posterior distribution without many grids for each parameter. This algorithm helps build trends for the solution surface where results for increasing and decreasing a parameter can be easily shown. In this study, GS was run with 3 grids of 3^{14} samples (which is ~4.8 million samples).

5. **Uniform Random Sampling**

Random sampling from a uniform distribution chooses samples within a lower and upper bound defined for each parameter. Each sample is equally probable which allows for a true random exploration of the parameter space. However, this sampling algorithm suffers in high dimensions, as the number of samples needed to represent the posterior distribution significantly increases with the number of parameters. With infinite sampling, this algorithm creates a general description of the solution surface, exploring both probable and many improbable points.

C) Filtering Mechanism

A posterior filtering of the samples helps create robust distributions that are less distorted from low probability points. For MCMC, samples that are less probable than the mean probability * 0.01 are discarded. Since there are many probable points selected from MCMC sampling, this filtering is more targeted to capturing only probable regions (ie. modes). For non-MCMC sampling, the mean probability is significantly lower than for the MCMC sampling, so a more general filtering is needed. Samples that were 1 std above the mean probability were kept while other lower probability points were discarded. The filtering cutoff does not significantly affect the results (provided the filtering threshold is small). However, the filtering threshold reduces computational bottlenecks by storing fewer datapoints, makes a more continuous final posterior, and also enables plotting to be computationally tractable.

Section S2): Results and Discussion

I) Convergence Metrics of Bayesian samplers

A) Integrated Autocorrelation function (ACT):

As promoted by Goodman & Weare[25], the integrated autocorrelation time (ACT) is a measure of sampling error on your results. Since ensemble samplers have correlated Markov chains, the effective number of independent samples needed to reach convergence must be approximated to understand how Monte Carlo error is impacting the sampling results. In this implementation, we use the emcee package[26] to calculate the ACT and follow their guidelines for determining convergence. As the sampler converges, the effective sample size estimation reduces in noise, and thus plateaus to a steady value. A dotted black line represents a heuristic to determine if the parameter has converged, where the effective sample size () is approximately the number of samples divided by 50. Ultimately, if the ACT calculation plateaus, convergence can be inferred.

The ACT diagnostic showed that each sampler converged. For ESS and AIES, a plateau is observed for every parameter with most parameters intersecting with the 50 heuristic line. In **Fig. S5 (a)** and **(e)**, the parameters ρ_e and B are shown to be in a transition to convergence, where the plateau is just starting and an intersection does not occur. Although this parameter is ambiguous in its convergence, the other parameters clearly infer the convergence of the sampler. The convergence diagnostic was only used for MCMC samplers, as these diagnostics are designed for MCMC sampler convergence.

(i) ESS

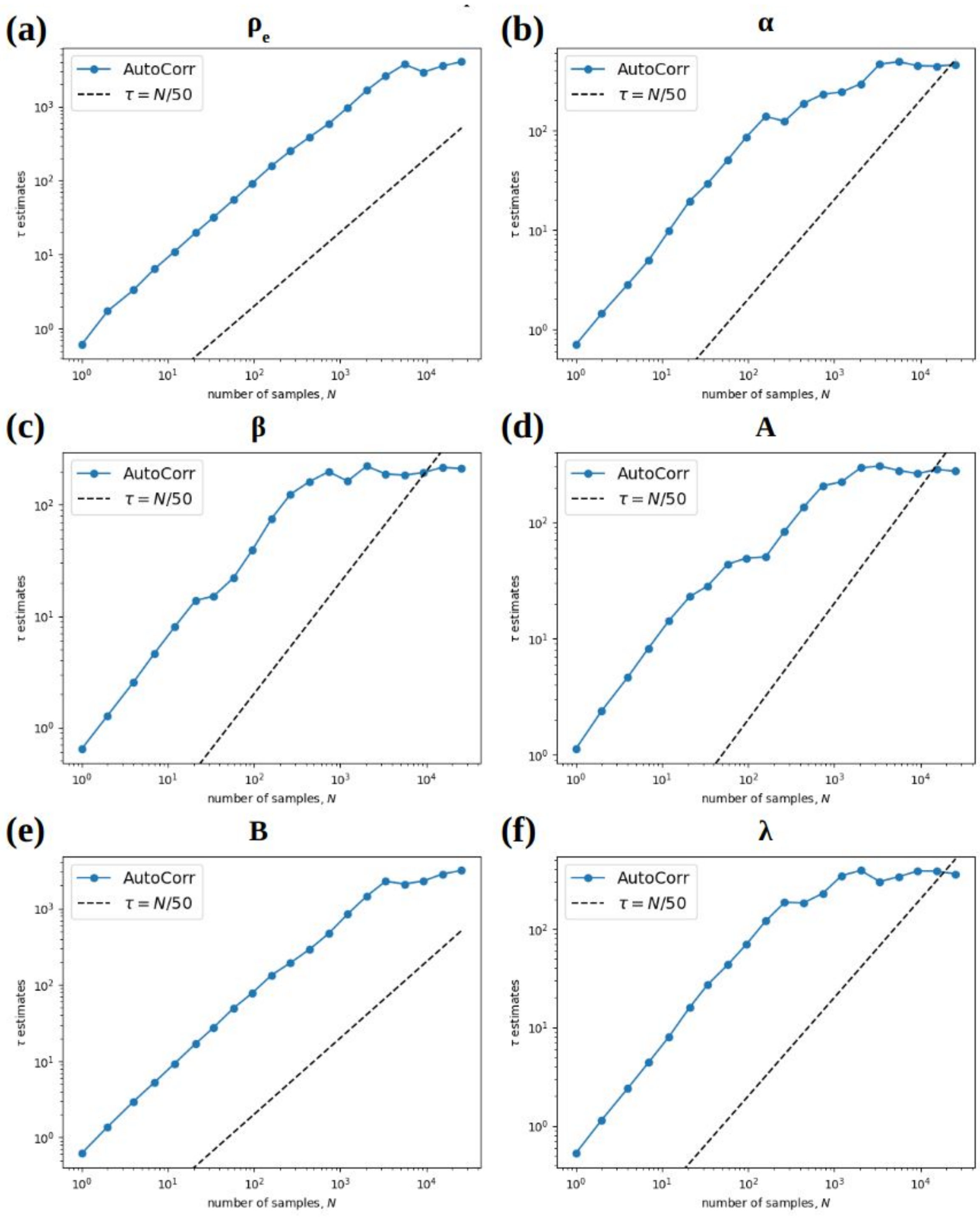

Figure S4: ACT graphs for each parameter obtained from ESS sampler (first 6 parameters)

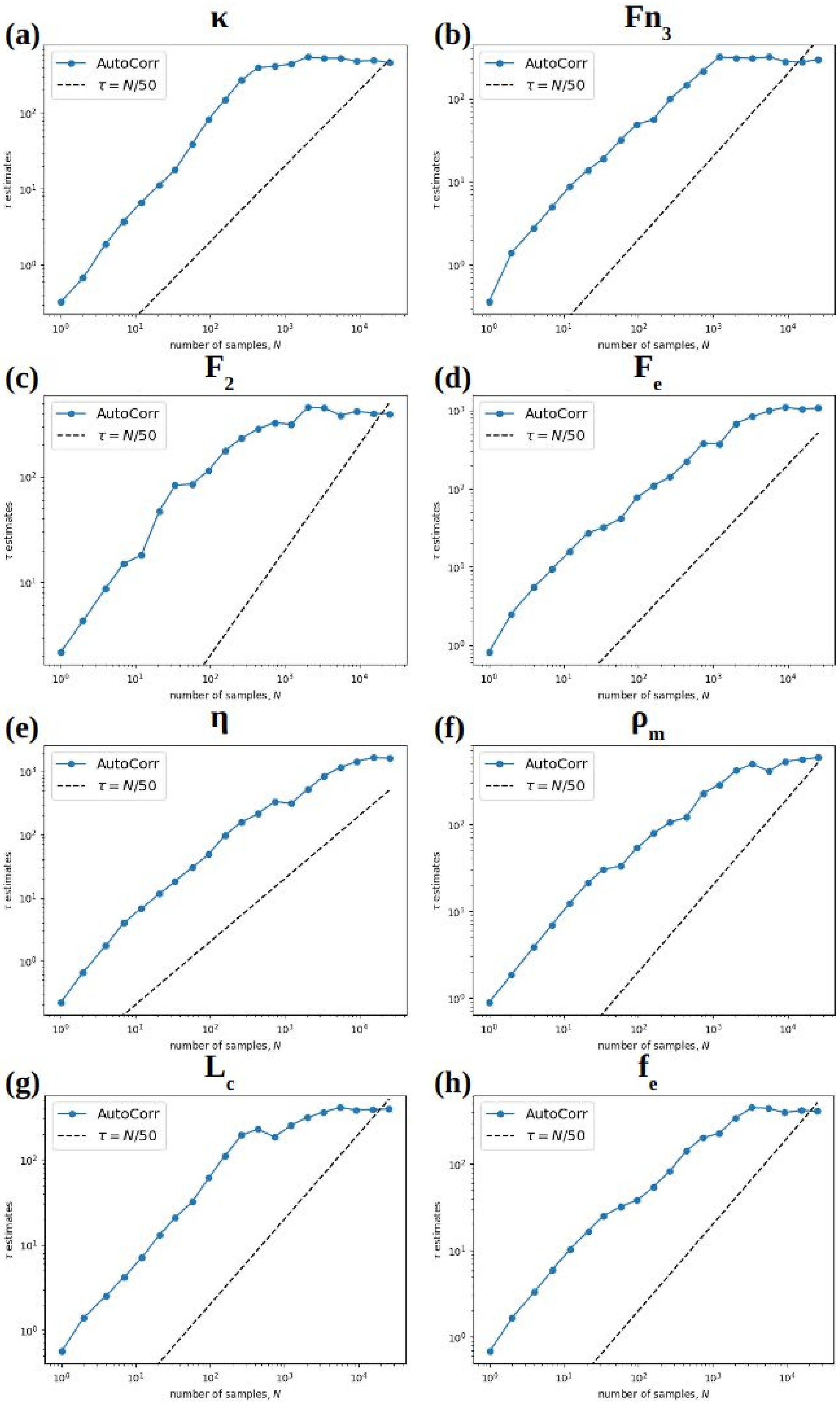

Figure S5: ACT graphs for each parameter obtained from ESS sampler (last 8 parameters)

(ii) AIES

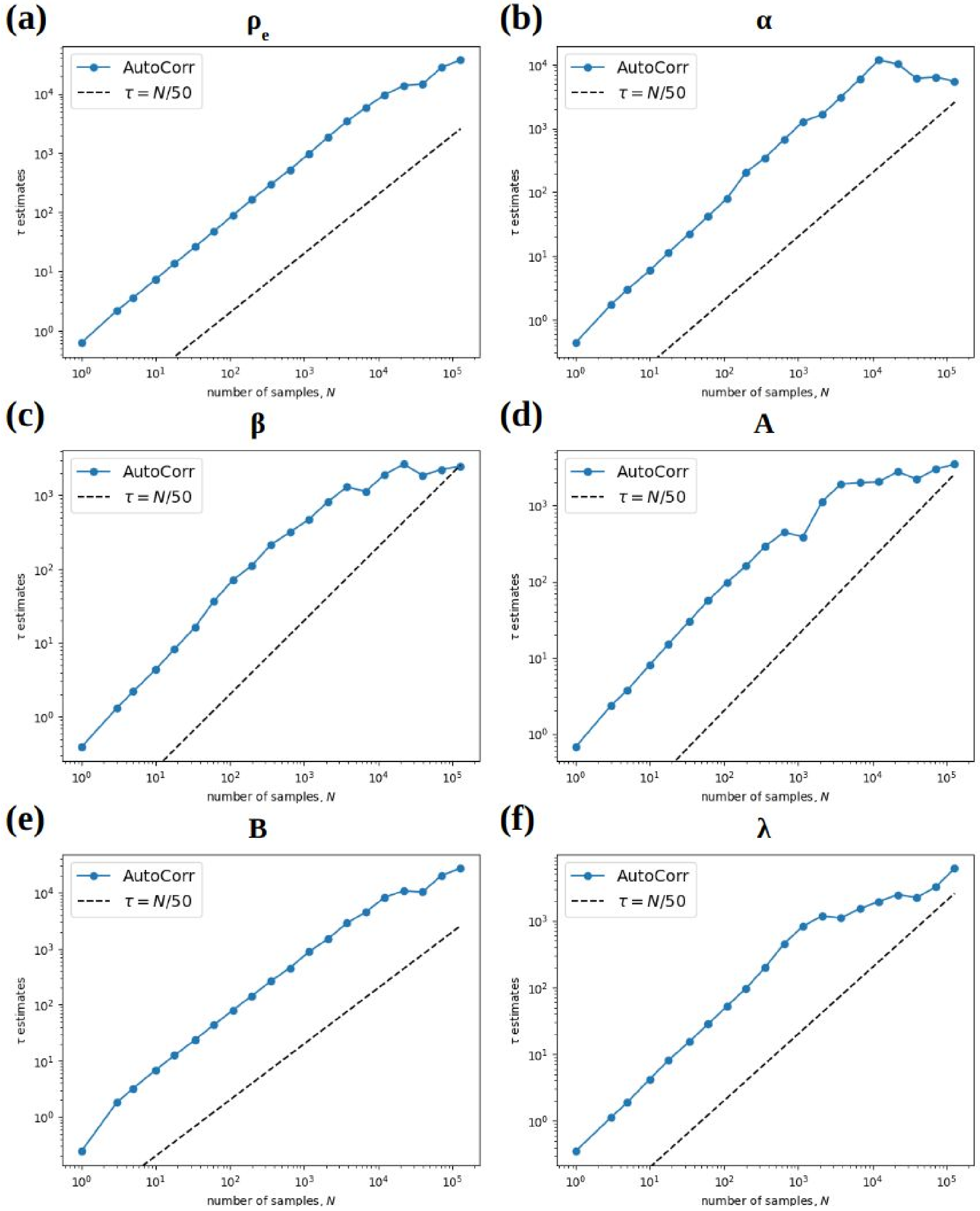

Figure S6: ACT graphs for each parameter obtained from AIES sampler (first 6 parameters)

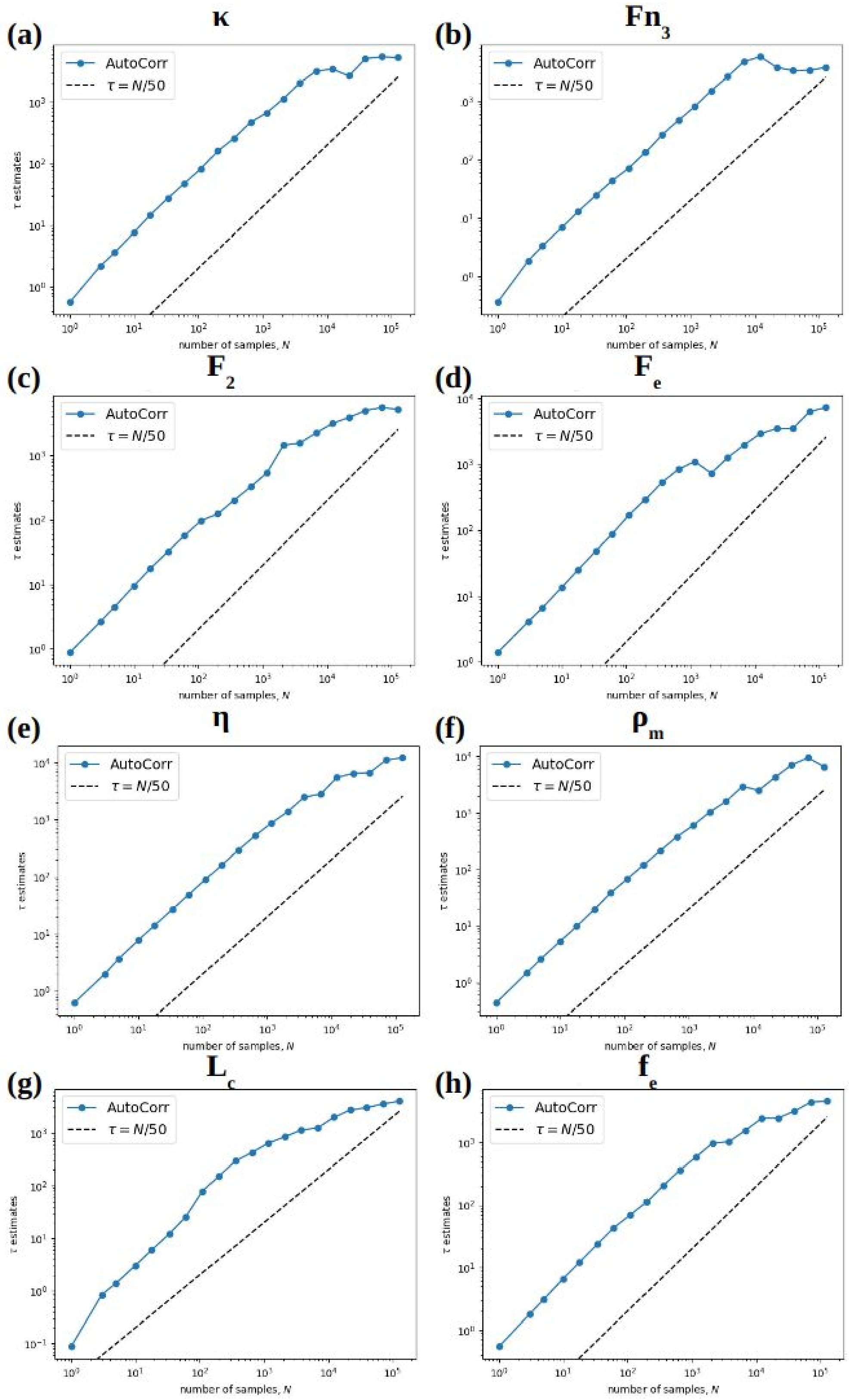

Figure S7: ACT graphs for each parameter obtained from AIES sampler (last 8 parameters)

(iii) MH

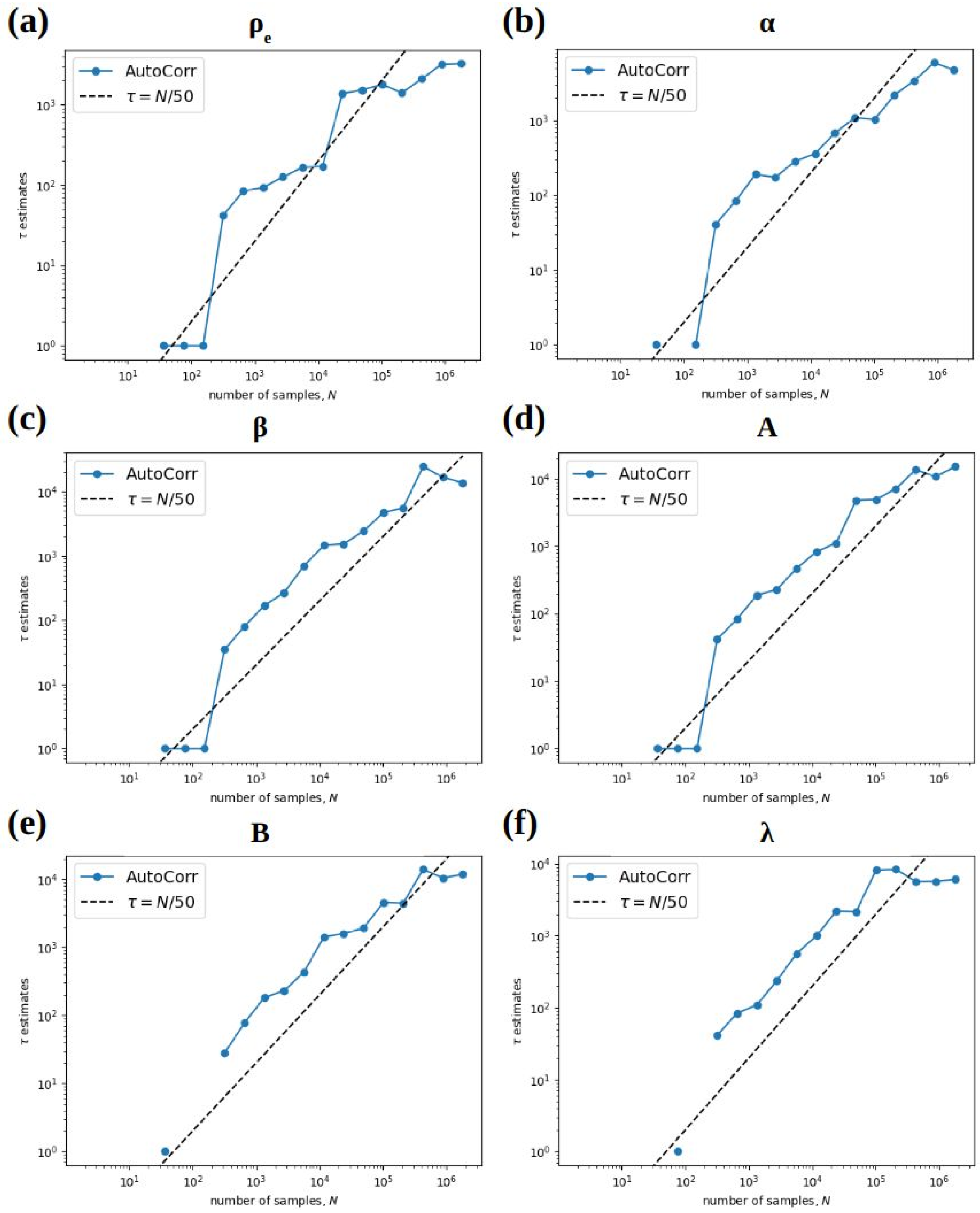

Figure S8: ACT graphs for each parameter obtained from MH sampler (first 6 parameters)

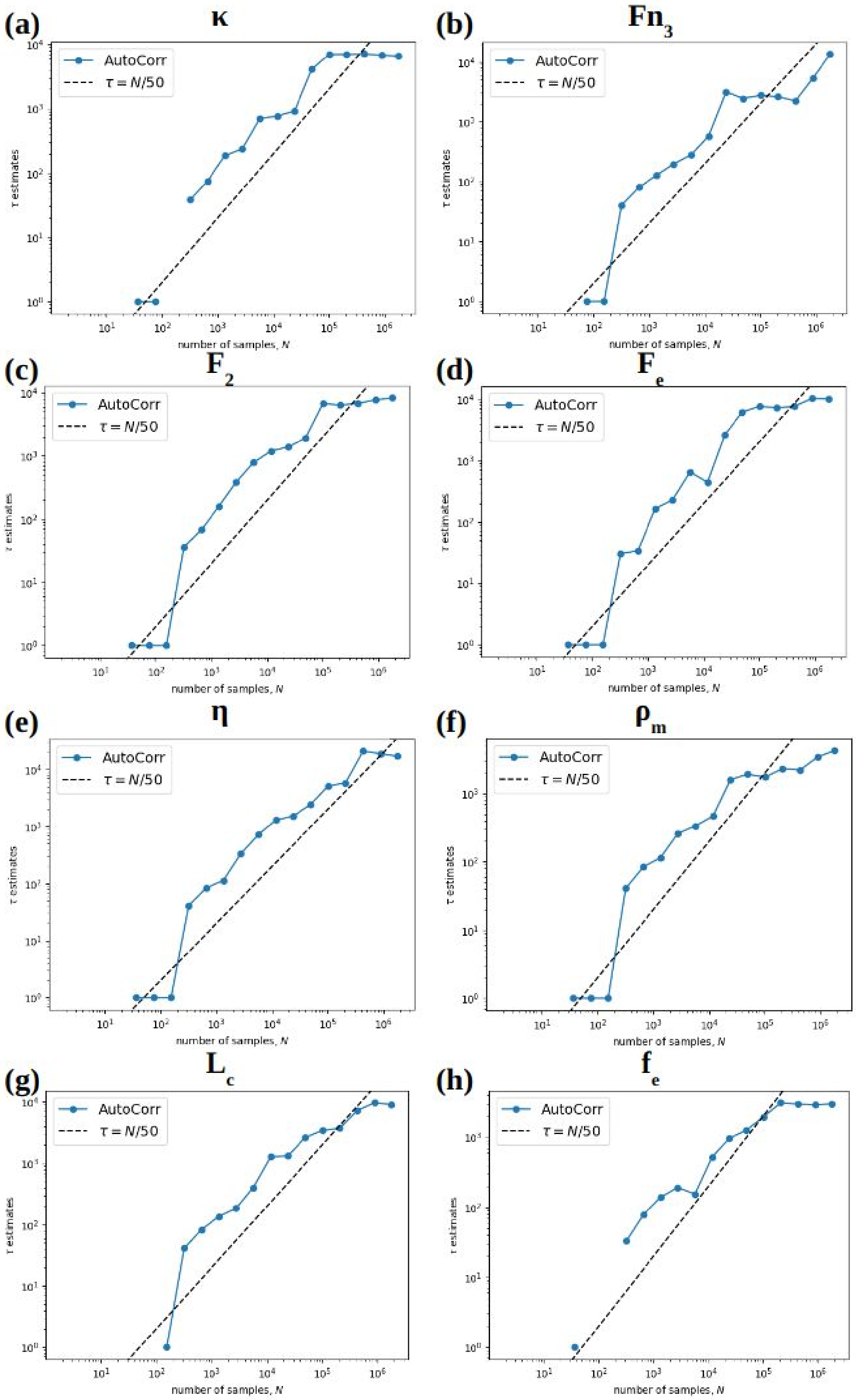

Figure S9: ACT graphs for each parameter obtained from MH sampler (last 8 parameters)

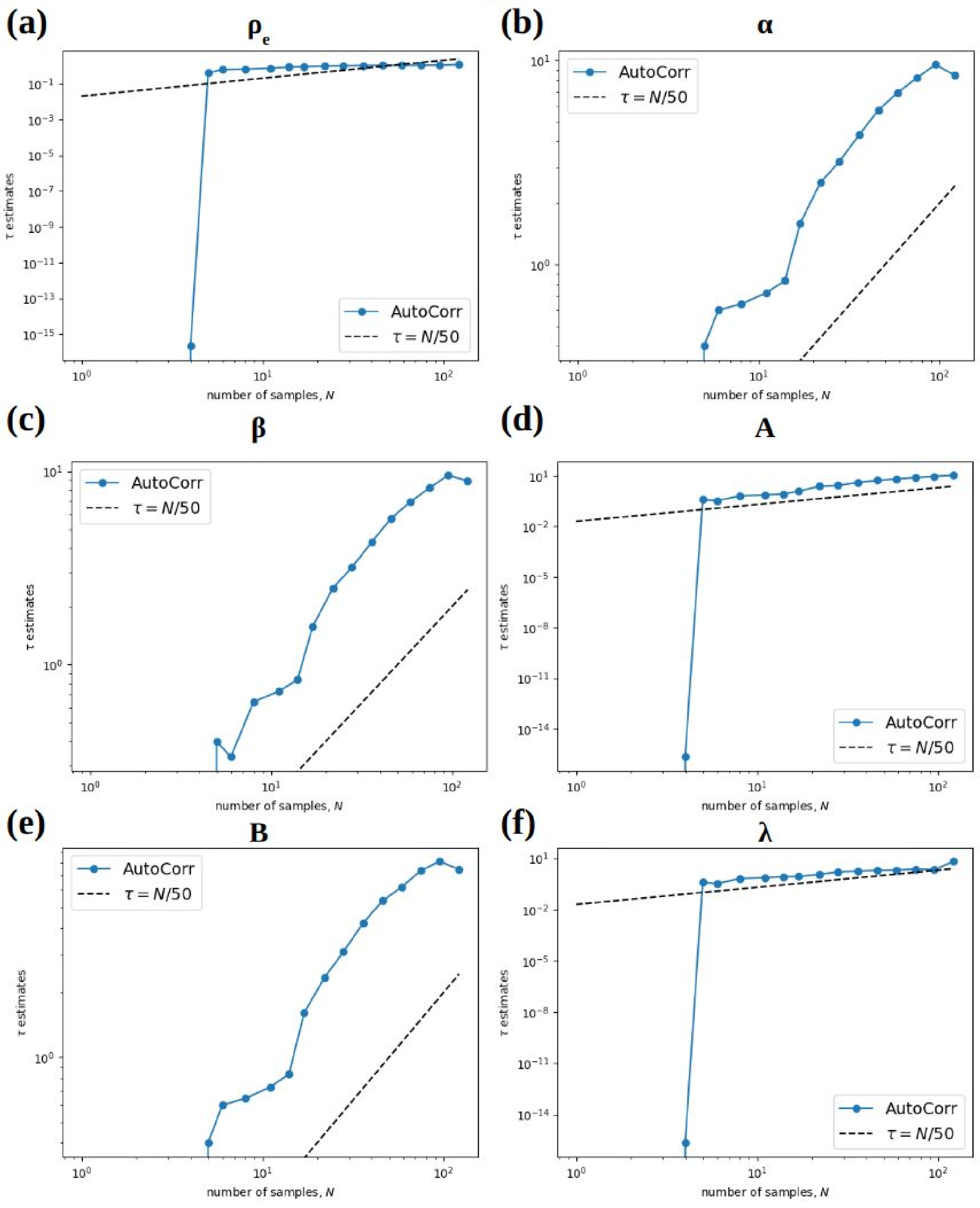

Figure S10: ACT graphs for each parameter obtained from URS sampler (first 6 parameters)

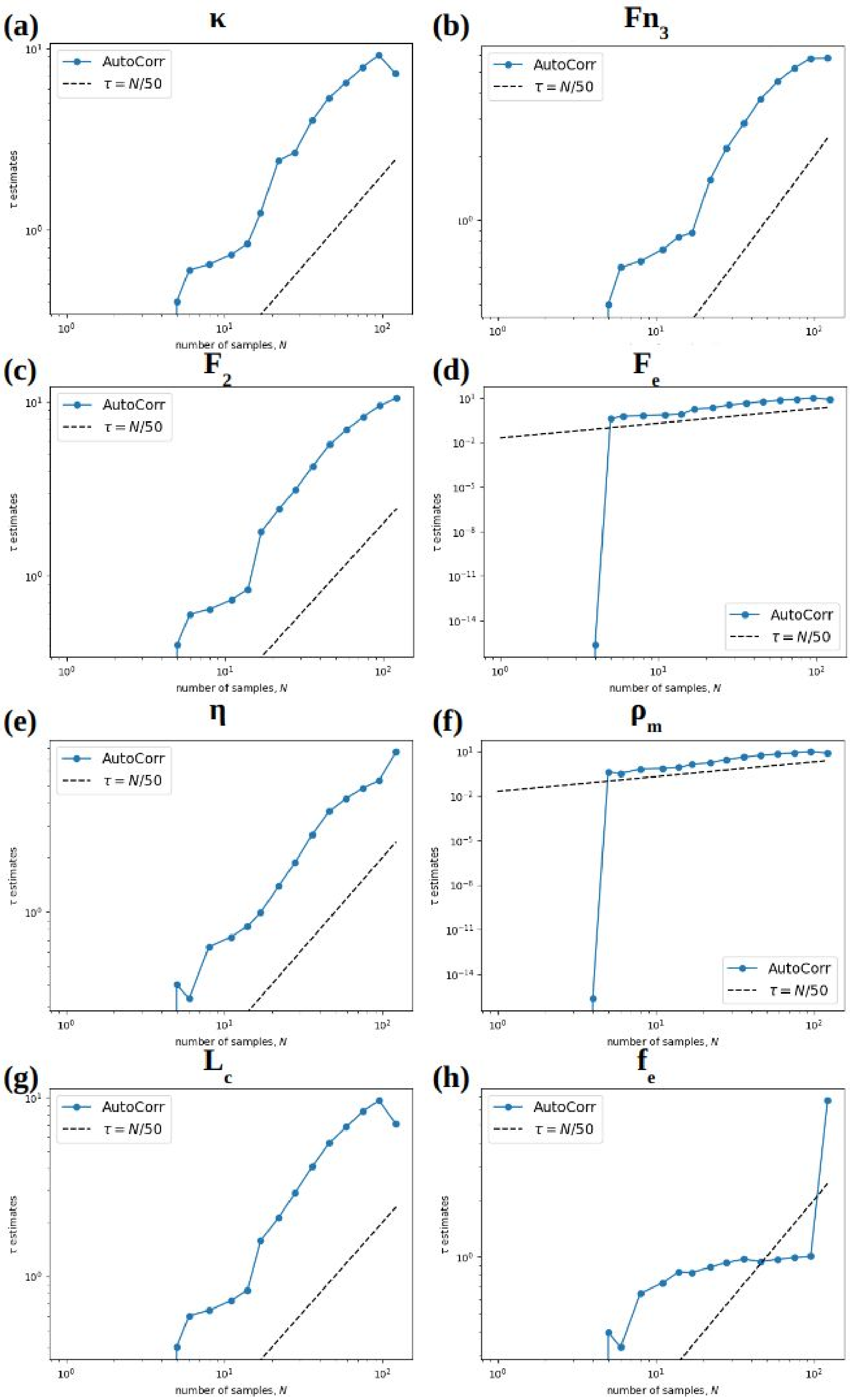

Figure S11: ACT graphs for each parameter obtained from URS sampler (last 8 parameters)

B) Geweke's Indices:

Geweke's indices is a variance based technique to quantify the evolution of ergodicity for the samplers. The assumption is that as the sampling algorithm converges towards the posterior distribution, the samplers explore less area and converge to similar points. The Geweke indices is a time series approach that compares the mean and variance of initial samples to samples at the end of the MCMC run[28]. In this implementation, the first 10% of samples () are compared to the last 50% of samples () using **equation SE5** to compute a z-score. Values that fall outside of ± 1 show that the samples fall outside of 1 standard deviation from the initial samples, indicating lack of convergence.

$$z = \frac{Mean(\theta_a) - Mean(\theta_b)}{\sqrt{Var(\theta_a) + Var(\theta_b)}} \text{ .... } \mathbf{Eq.\ (SE17)}$$

For each parameter, the z score is plotted against the number of samples for the left hand plot and the right hand plot shows the percentage of samples that fall outside 1 std. Every parameter shown falls to 0% values outside 1 std in every sampler. However, it is worth noting that rhoe is the most ergodic parameter as shown in **Fig. S6 (a)** and **Fig. S7 (a)**. This ergodicity is not shown in MH, as the sampler did not expand to find the second mode. This shows that rhoe is an important parameter for varying when trying to find multiple solutions. The Geweke convergence diagnostic was used only for MCMC samplers, as ergodicity does not infer convergence for a random sampling.

(i) ESS

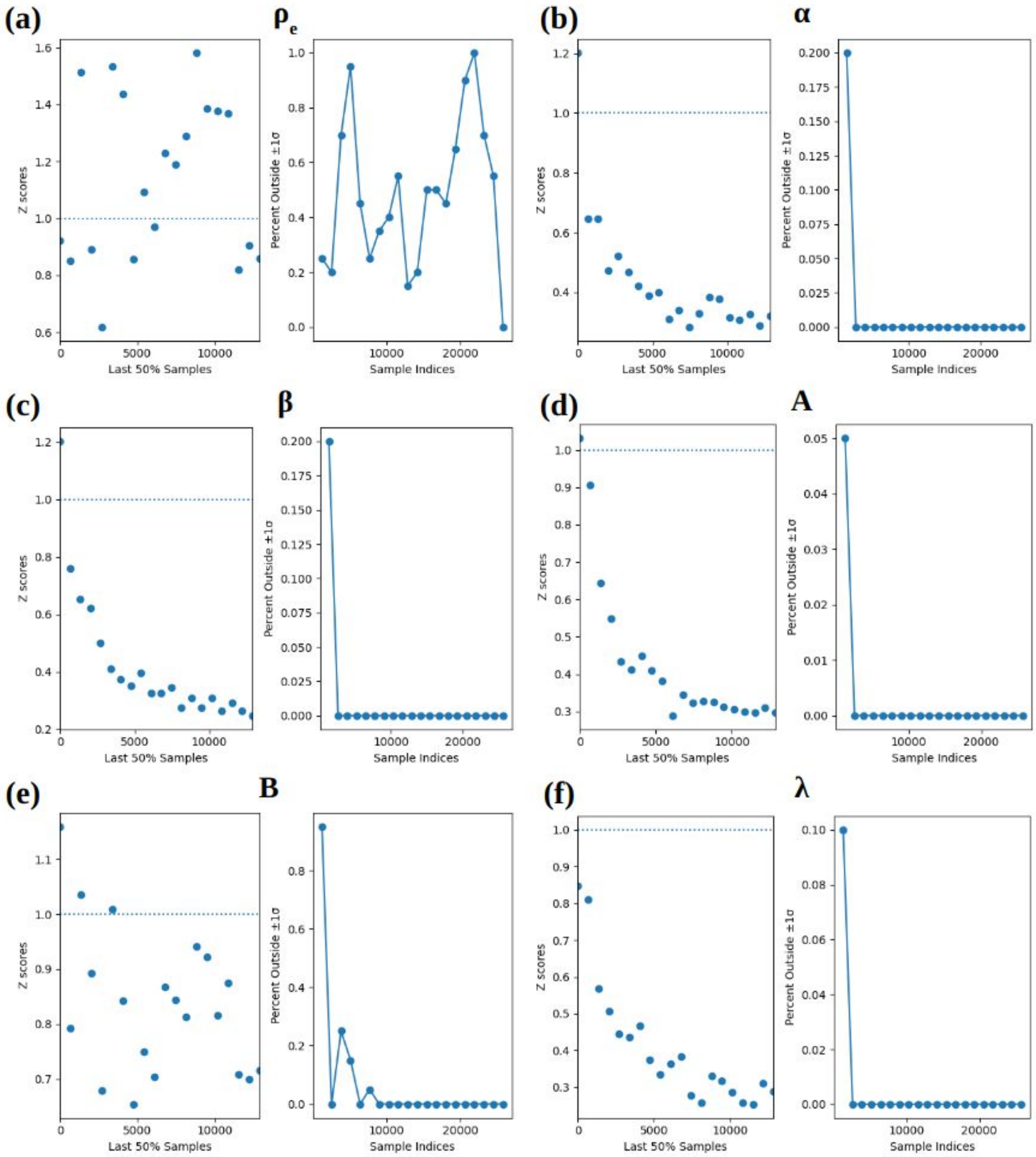

Figure S12: Geweke's indices' graphs for first 6 parameters from ESS sampler.

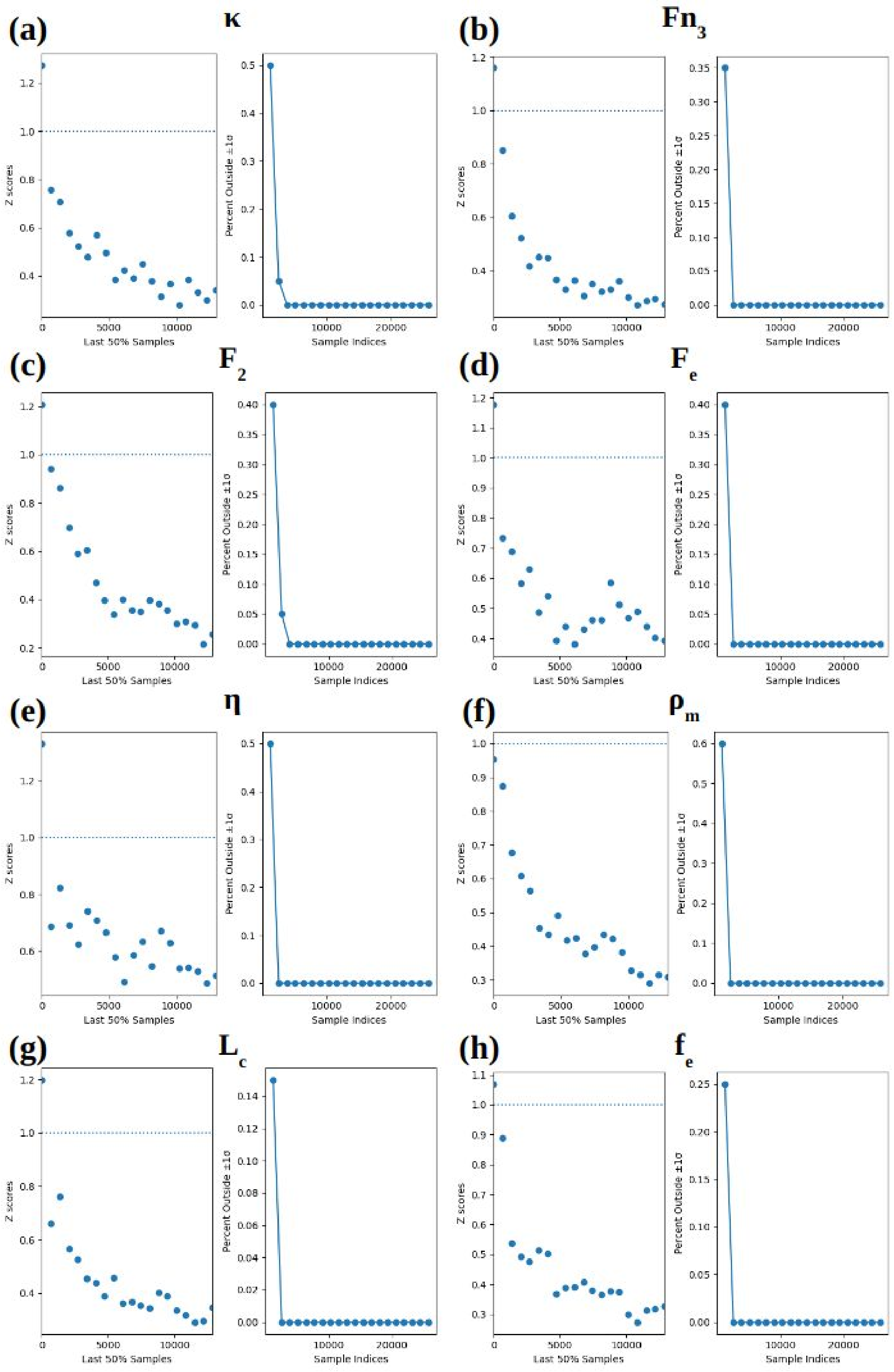

Figure S13: Geweke's indices' graphs for last 8 parameters from ESS sampler.

(ii) AIES

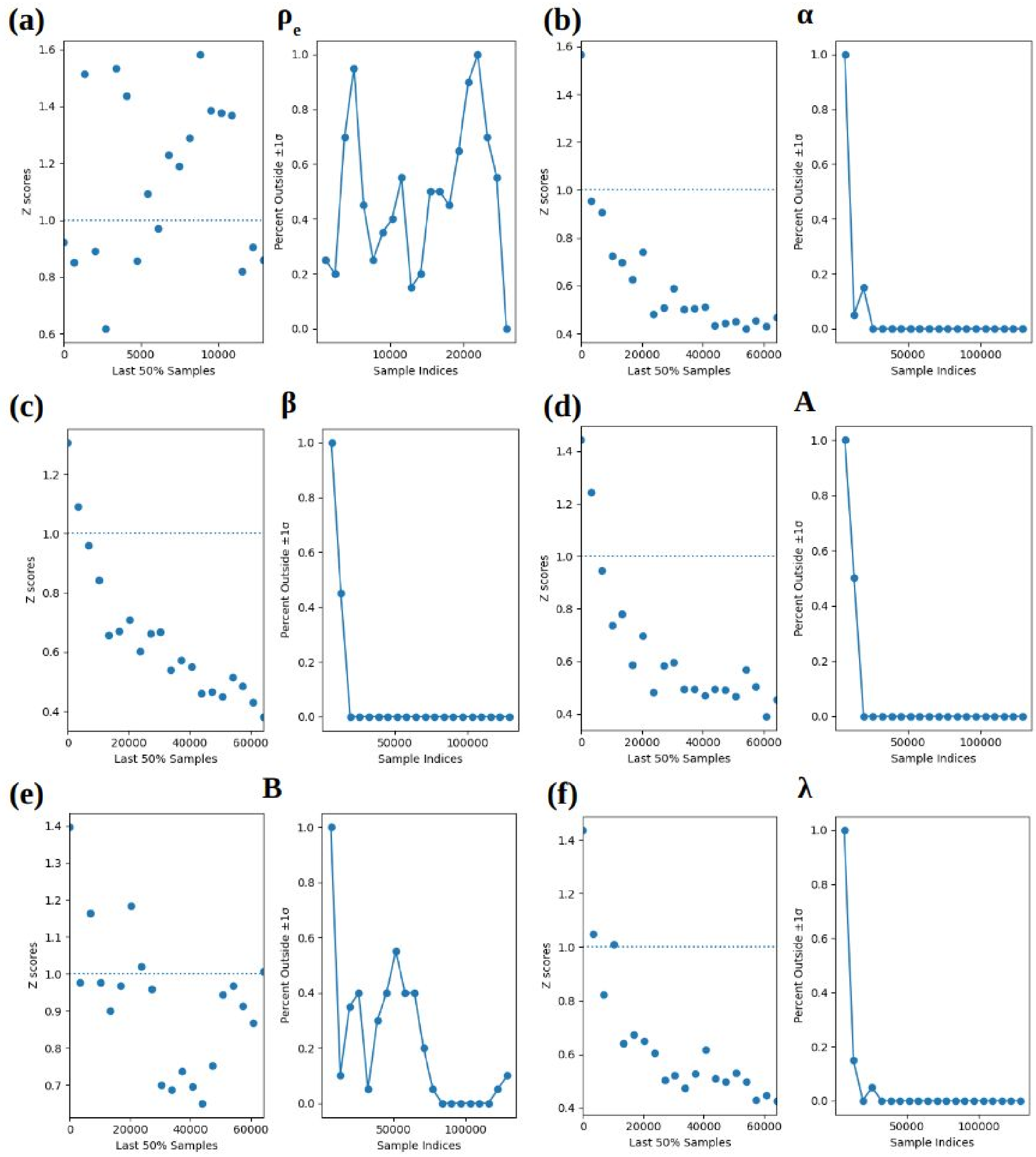

Figure S14: Geweke's indices' graphs for first 6 parameters from ESS sampler.

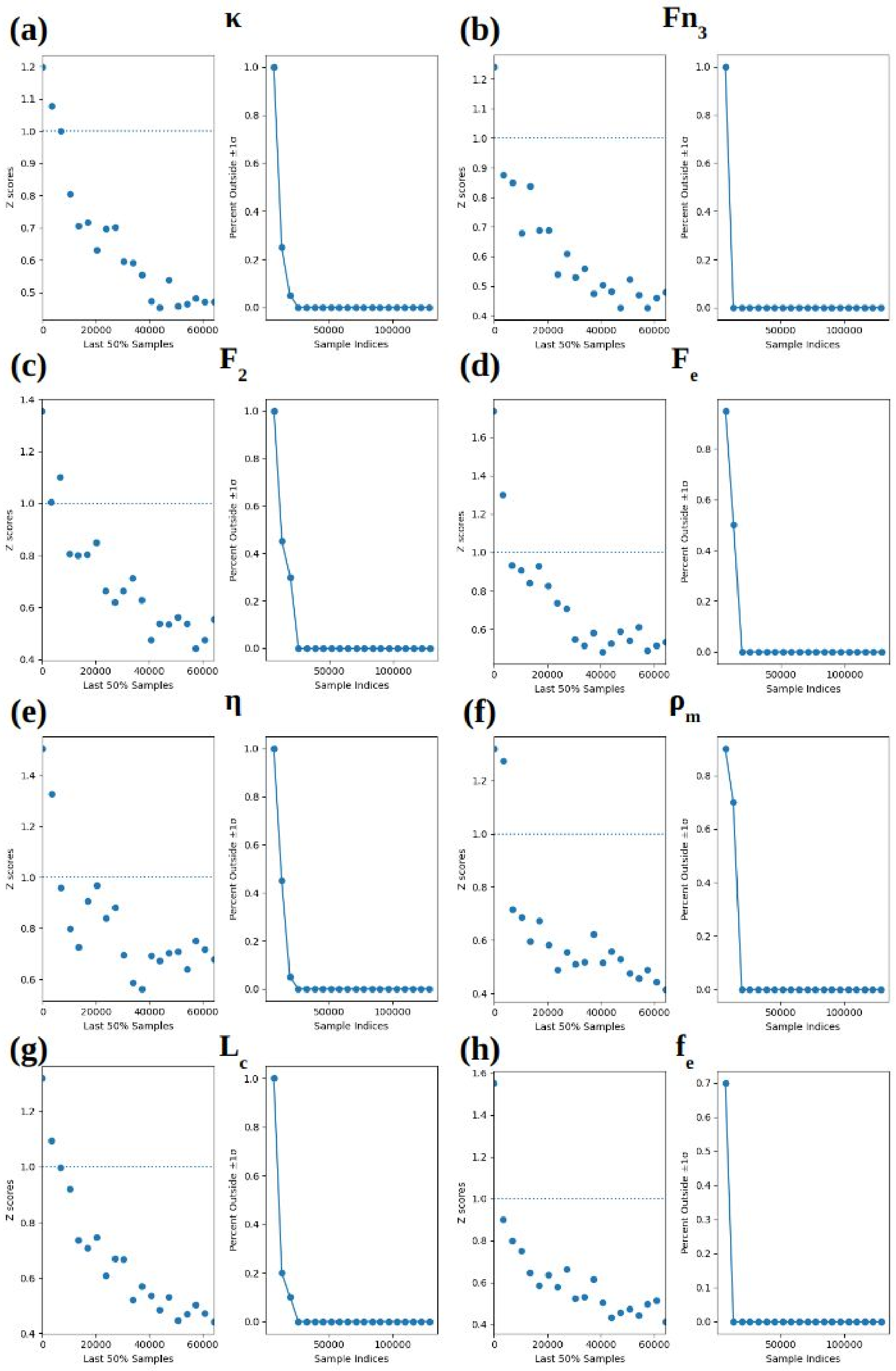

Figure S15: Geweke's indices' graphs for last 8 parameters from ESS sampler.

(iii) MH

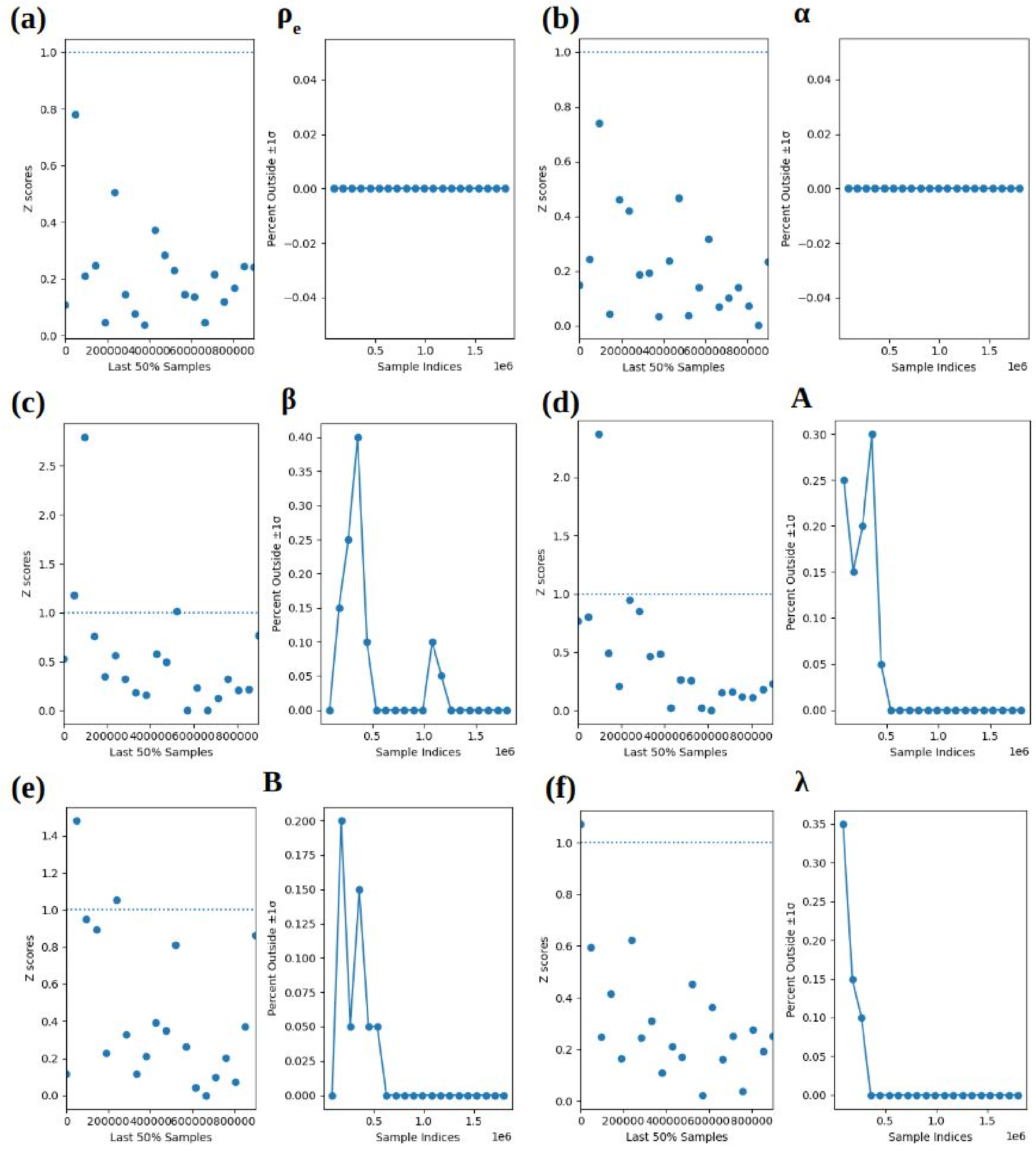

Figure S16: Geweke's indices' graphs for first 6 parameters from MH sampler.

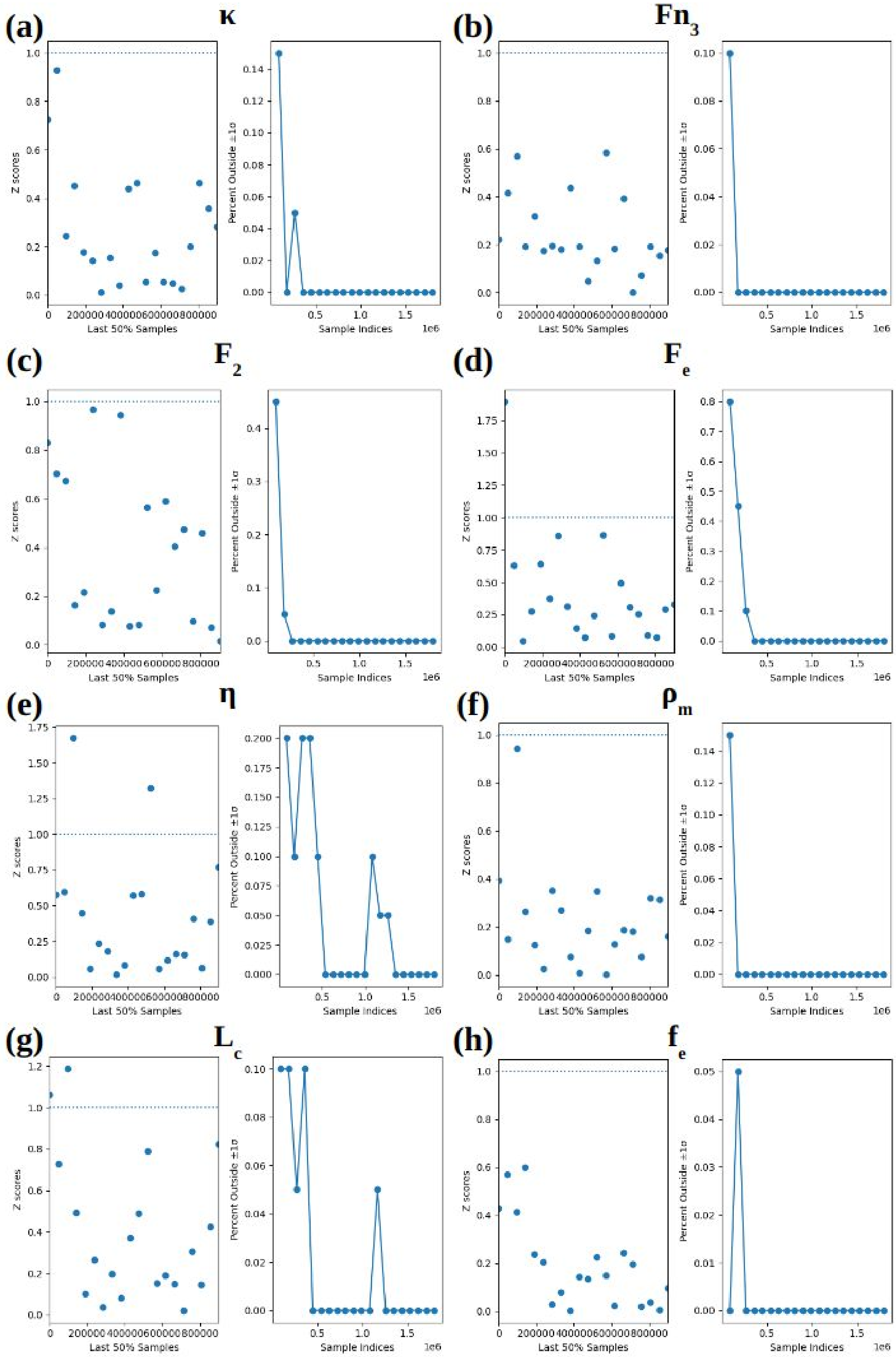

Figure S17: Geweke's indices' graphs for last 8 parameters from MH sampler.

(iv) URS

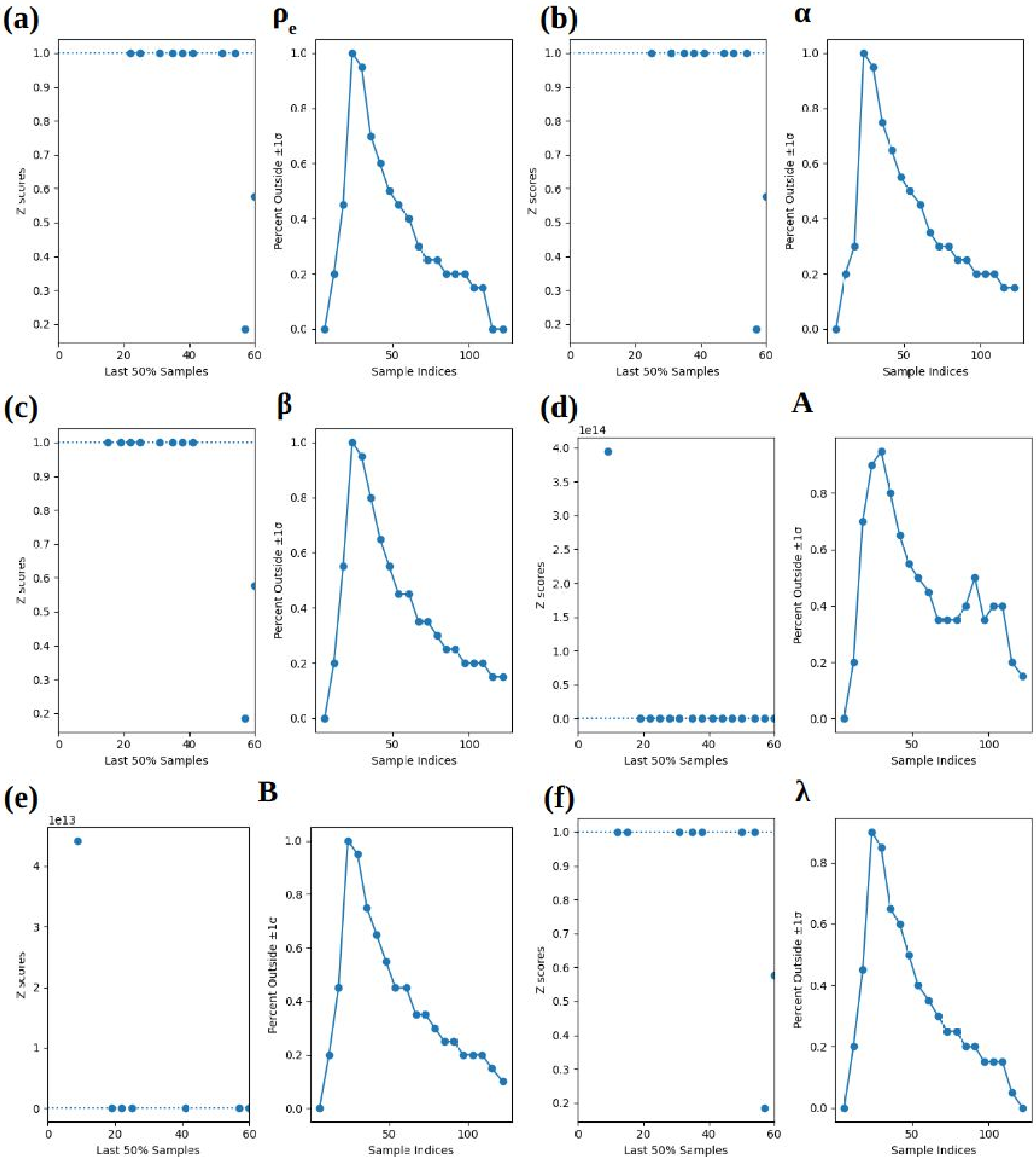

Figure S18: Geweke's indices' graphs for first 6 parameters from URS sampler.

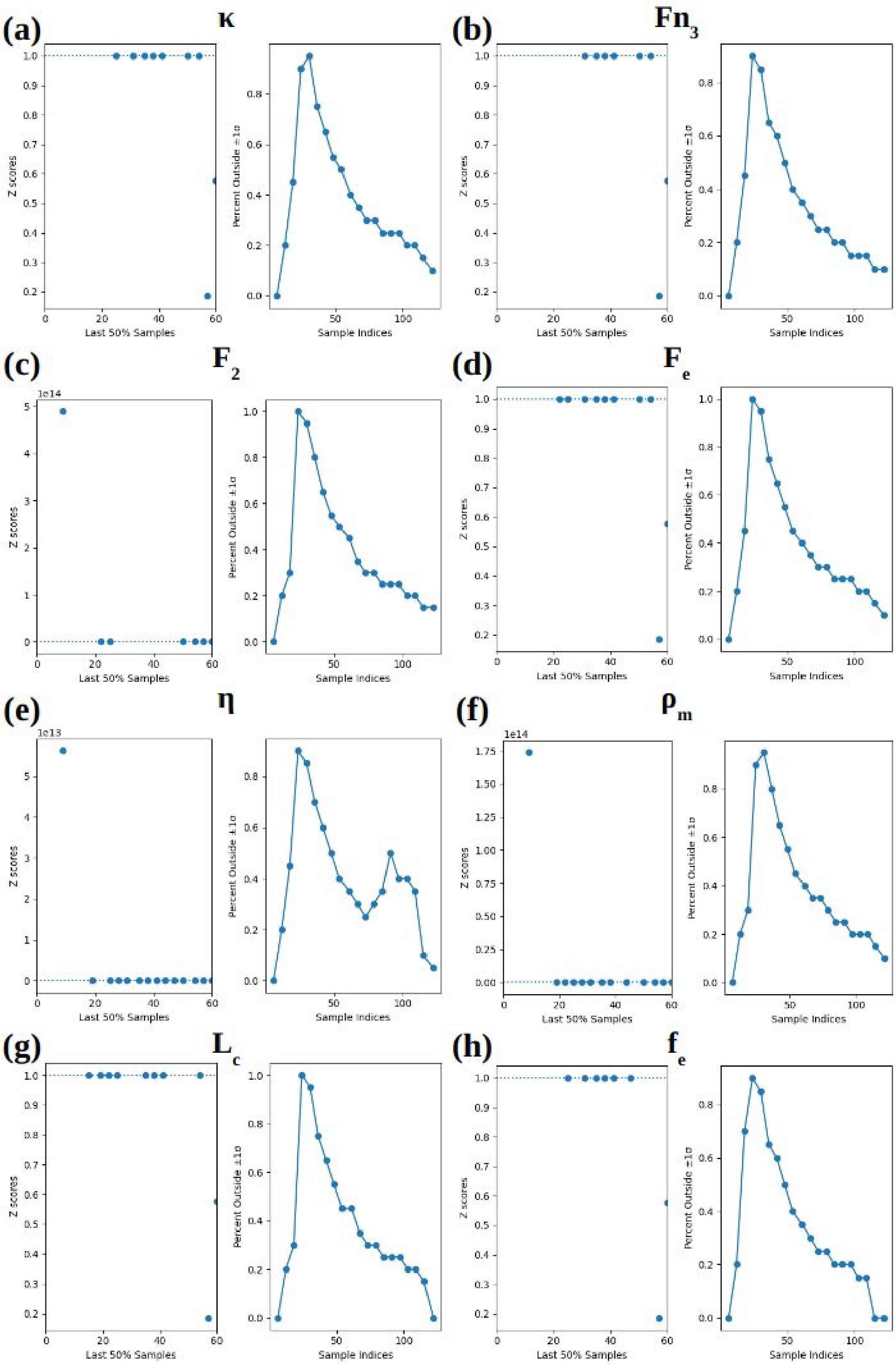

Figure S19: Geweke's indices' graphs for last 8 parameters from URS sampler.

(v) GS

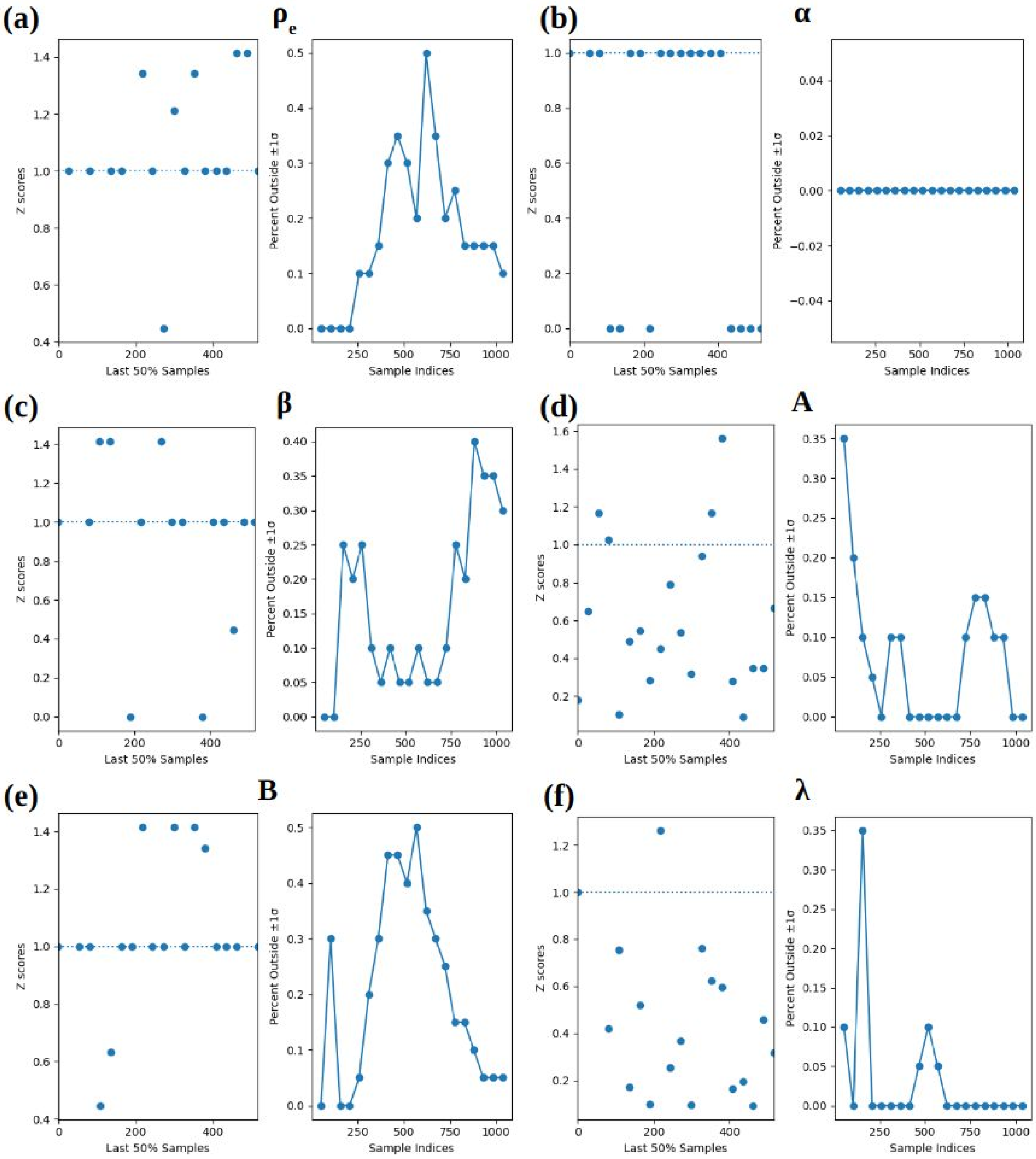

Figure S20: Geweke's indices' graphs for first 6 parameters from GS sampler.

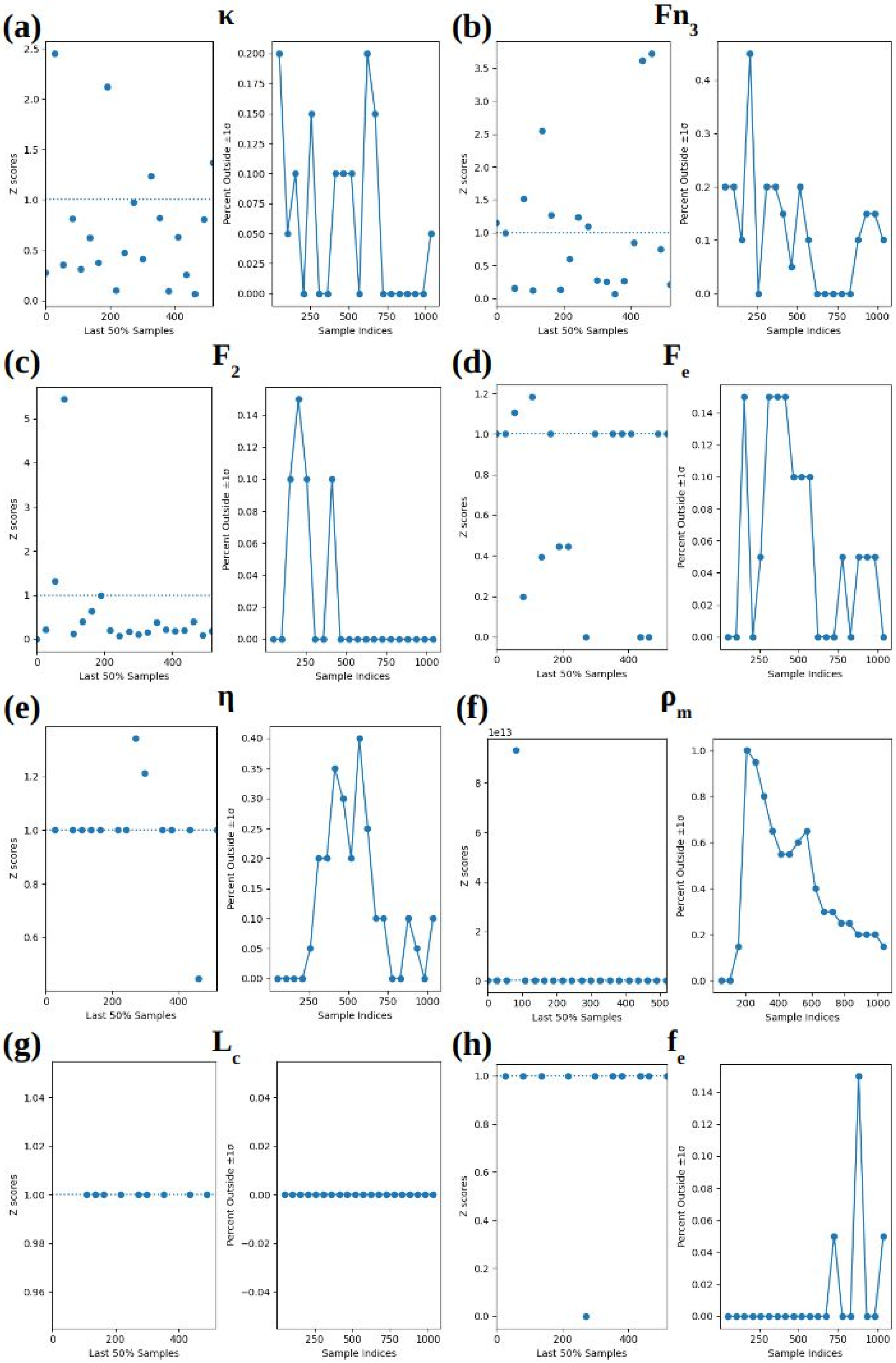

Figure S21: Geweke's indices' graphs for last 8 parameters from GS sampler.

II) Corner Plots

Corner plots show scatter plots, histograms, and a correlation heatmap between parameters and properties to show the behavior of the Bayesian UQ sampling. Scatter plots show the densely sampled regions and it shows a general path between densely sampled regions. The histograms show the uncertainty and multimodality of the parameter/property. The Pearson correlation coefficient shows linear relationships between parameters and properties on the grid. To create these plots, 100,000 samples were randomly selected from the posterior distributions to create a simplified representation of the sampling. The scatter plots, histograms, and heatmaps were created with matplotlib but were combined together after generation in an image editing software. The parameters and properties are separated to decrease the size of the image.

C44 was accurately predicted with low variation in mode 1 at 42.7 GPa, but was inaccurate with a large variation in mode 2, with MAP values ranging between 38-41 GPa. In contrast, mode 2 provided slightly more accurate density predictions than mode 1 (19.3 vs. 19.2 gm/cc, with a target value of 19.3 gm/cc), indicating a tradeoff between mechanical properties and density in the EAM potential.

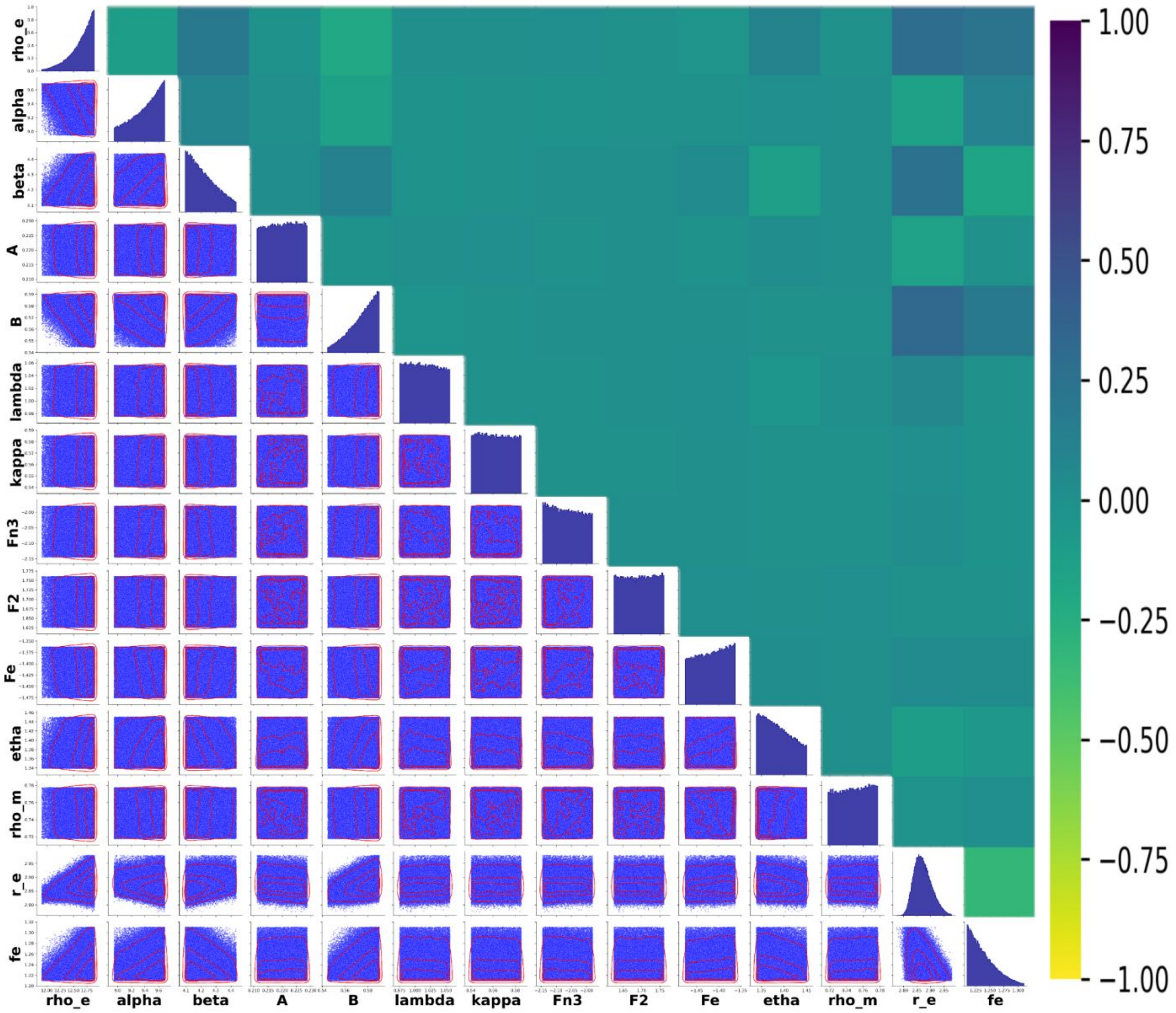

Figure S22: Corner plot for all parameters with URS sampler.

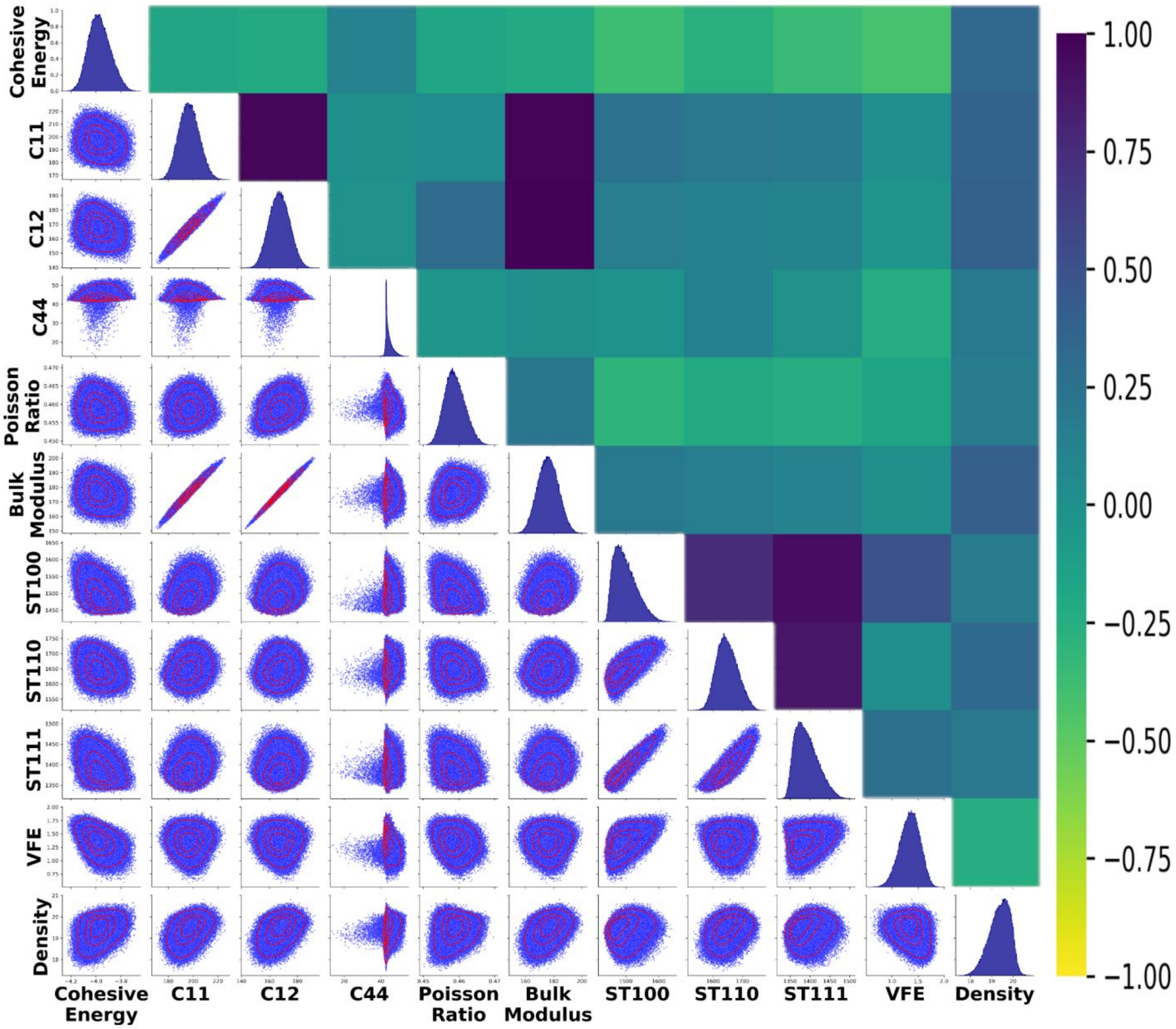

Figure S23: Corner plot for all properties with URS sampler.

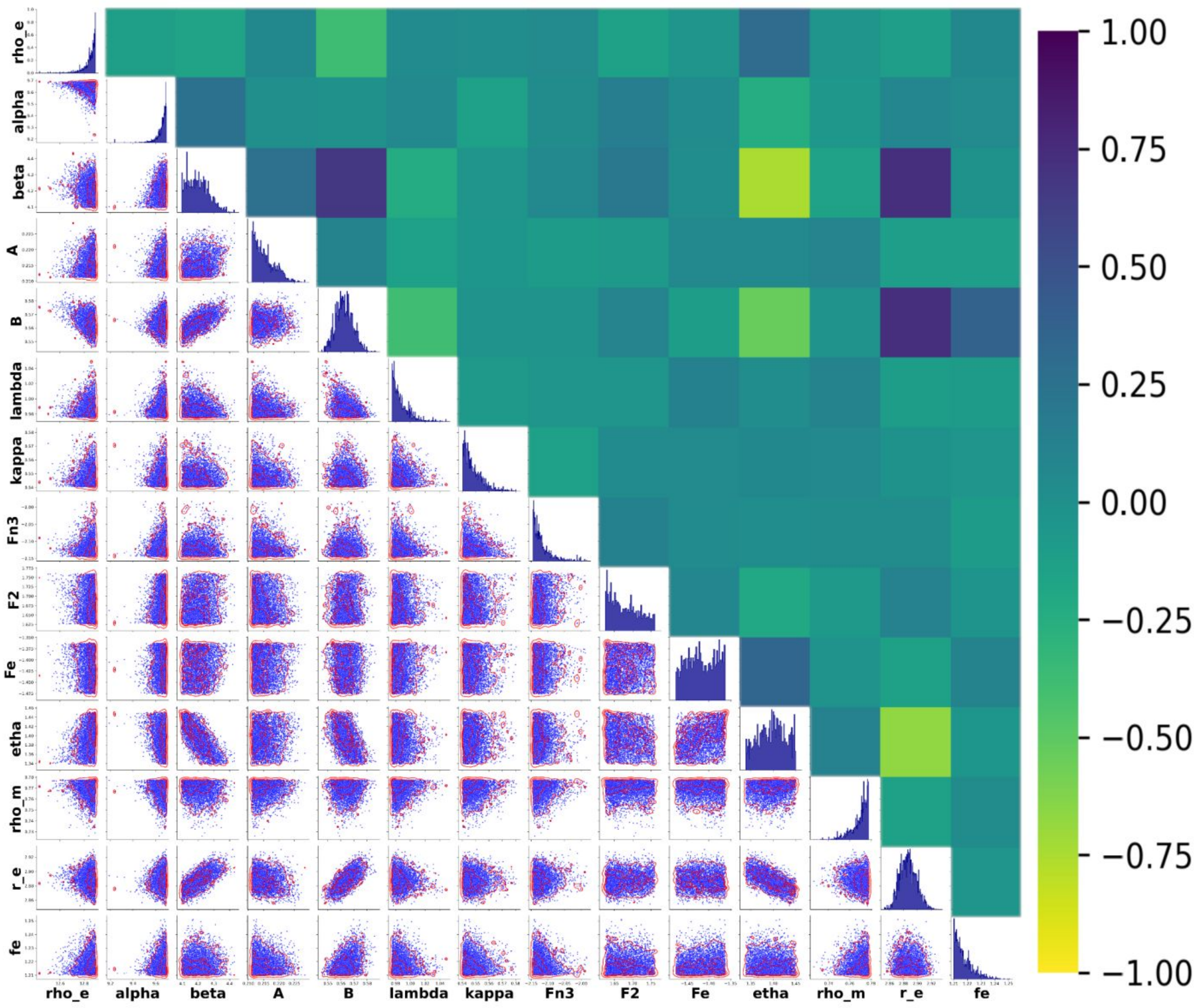

Figure S24: Corner plot for all parameters with MH sampler.

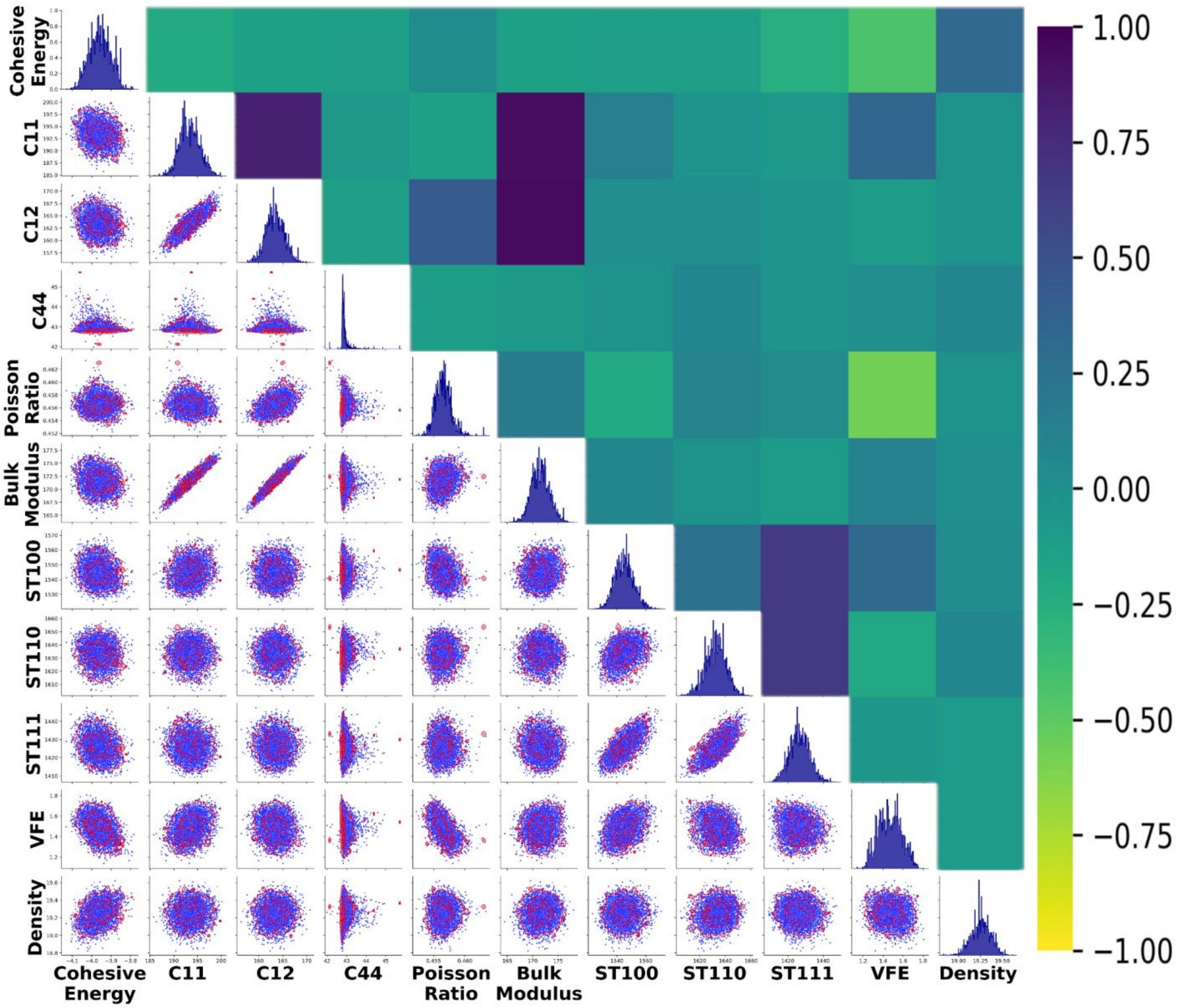

Figure S25: Corner plot for all properties with MH sampler.

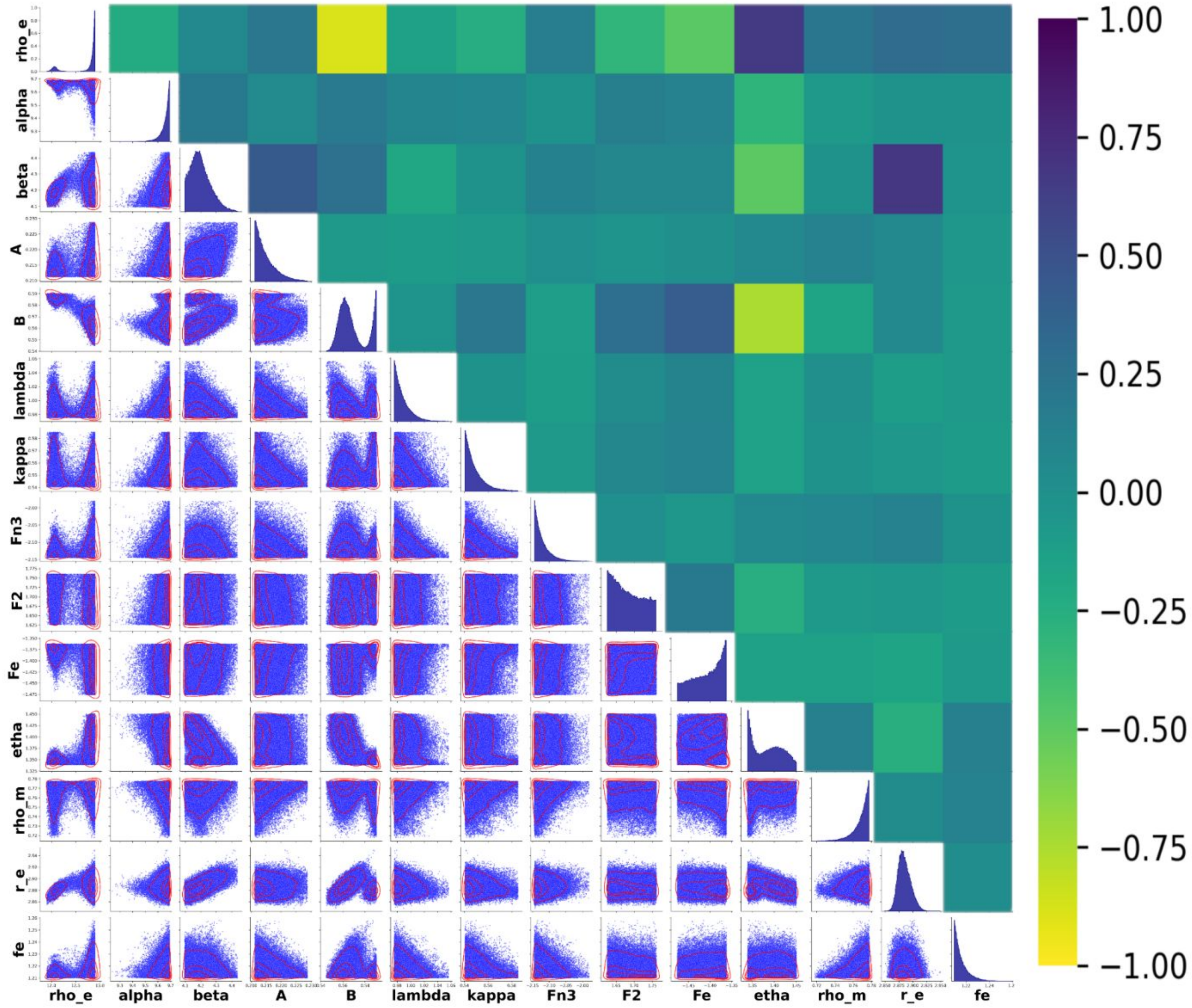

Figure S26: Corner plot for all parameters with ESS sampler.

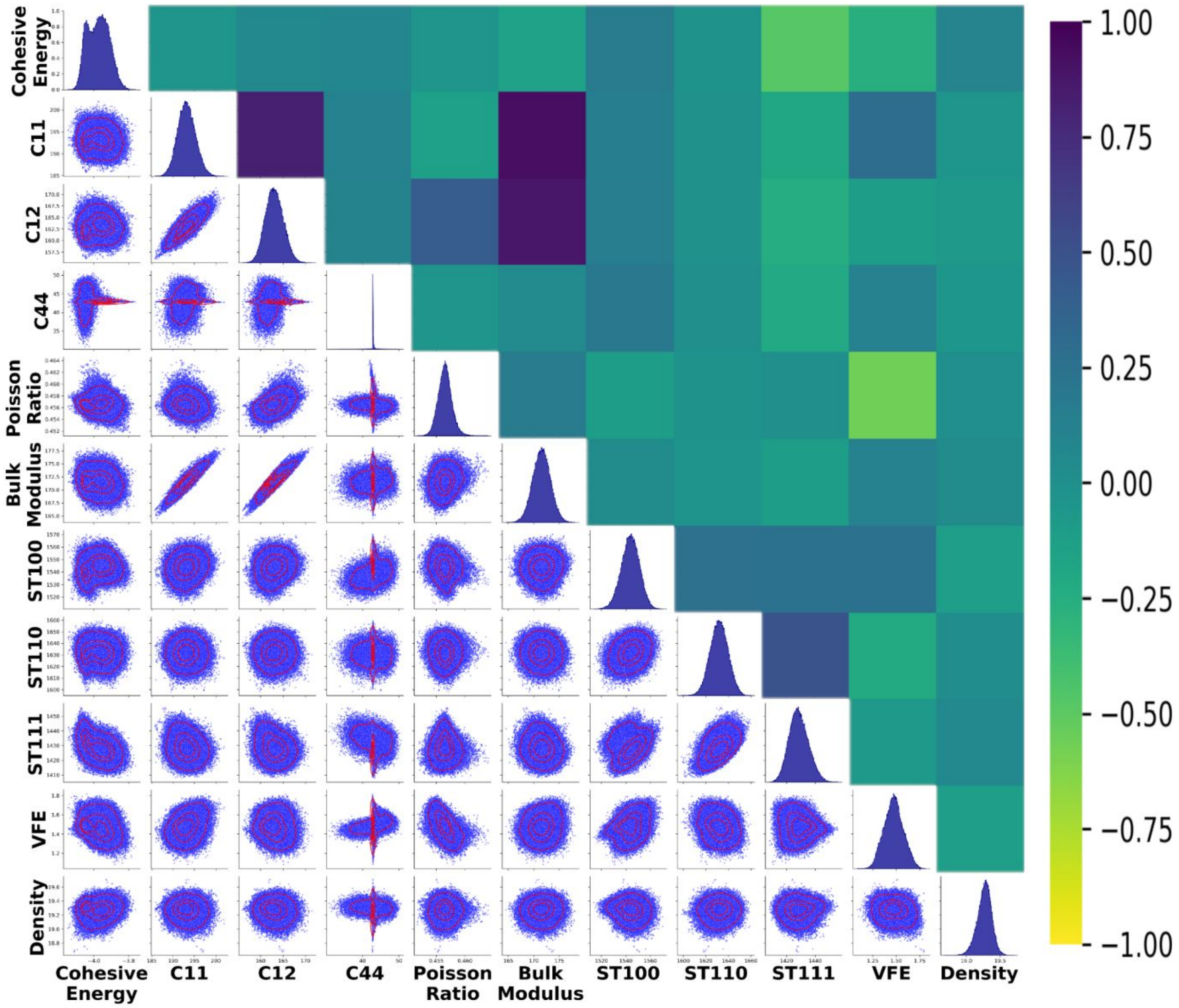

Figure S27: Corner plot for all properties with ESS sampler.

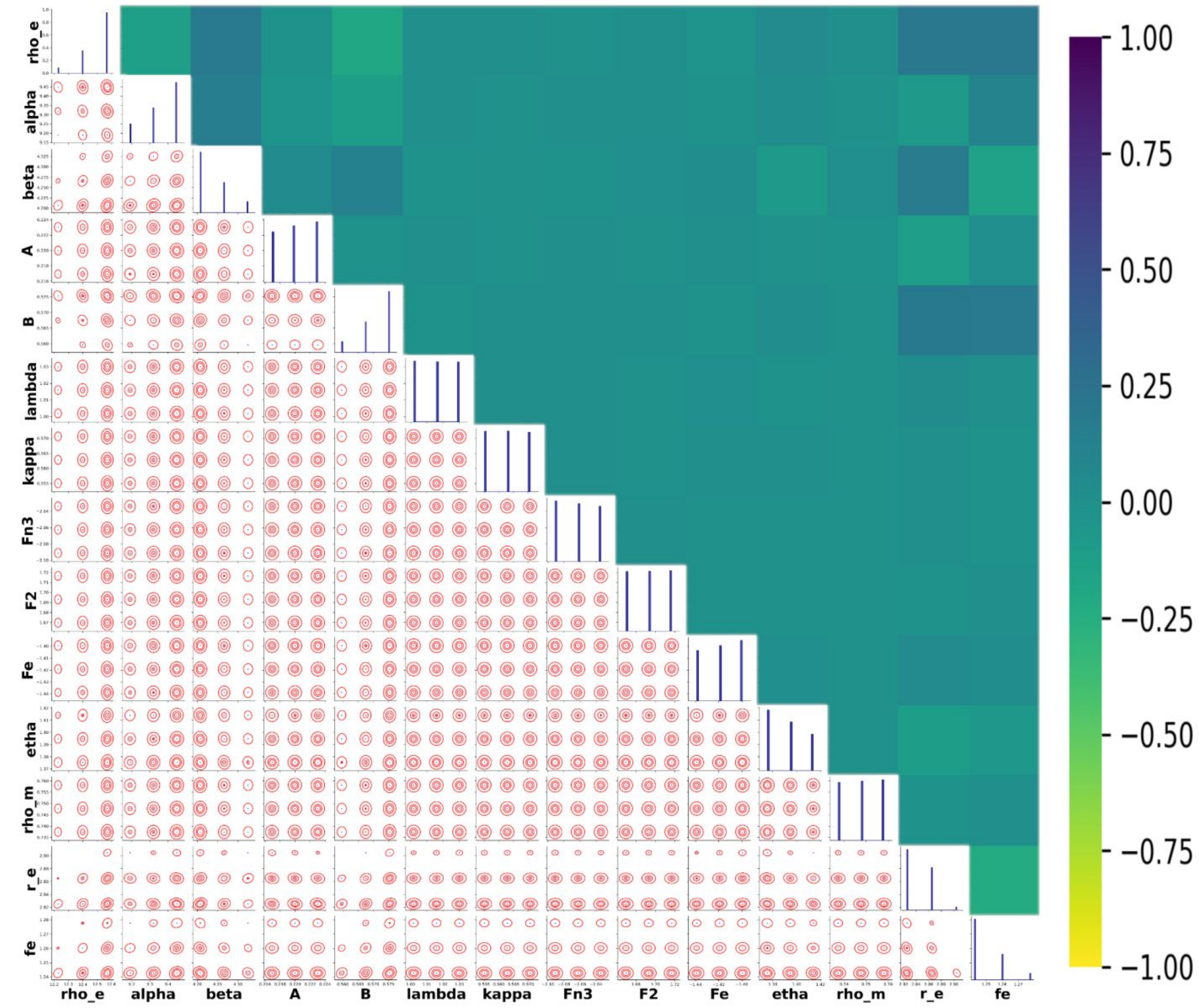

Figure S28: Corner plot for all parameters with grid sampler.

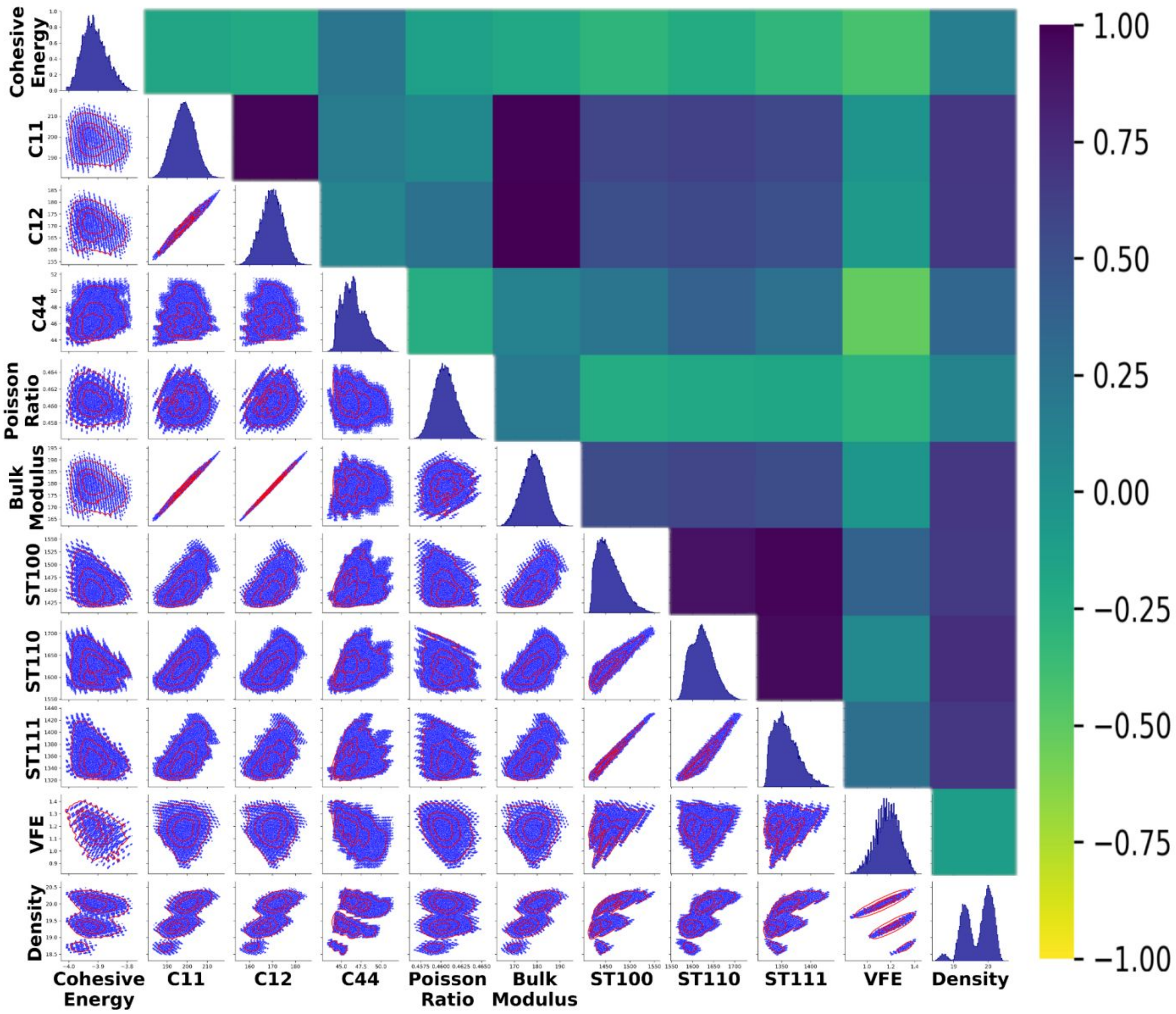

Figure S29: Corner plot for all properties with grid sampler.

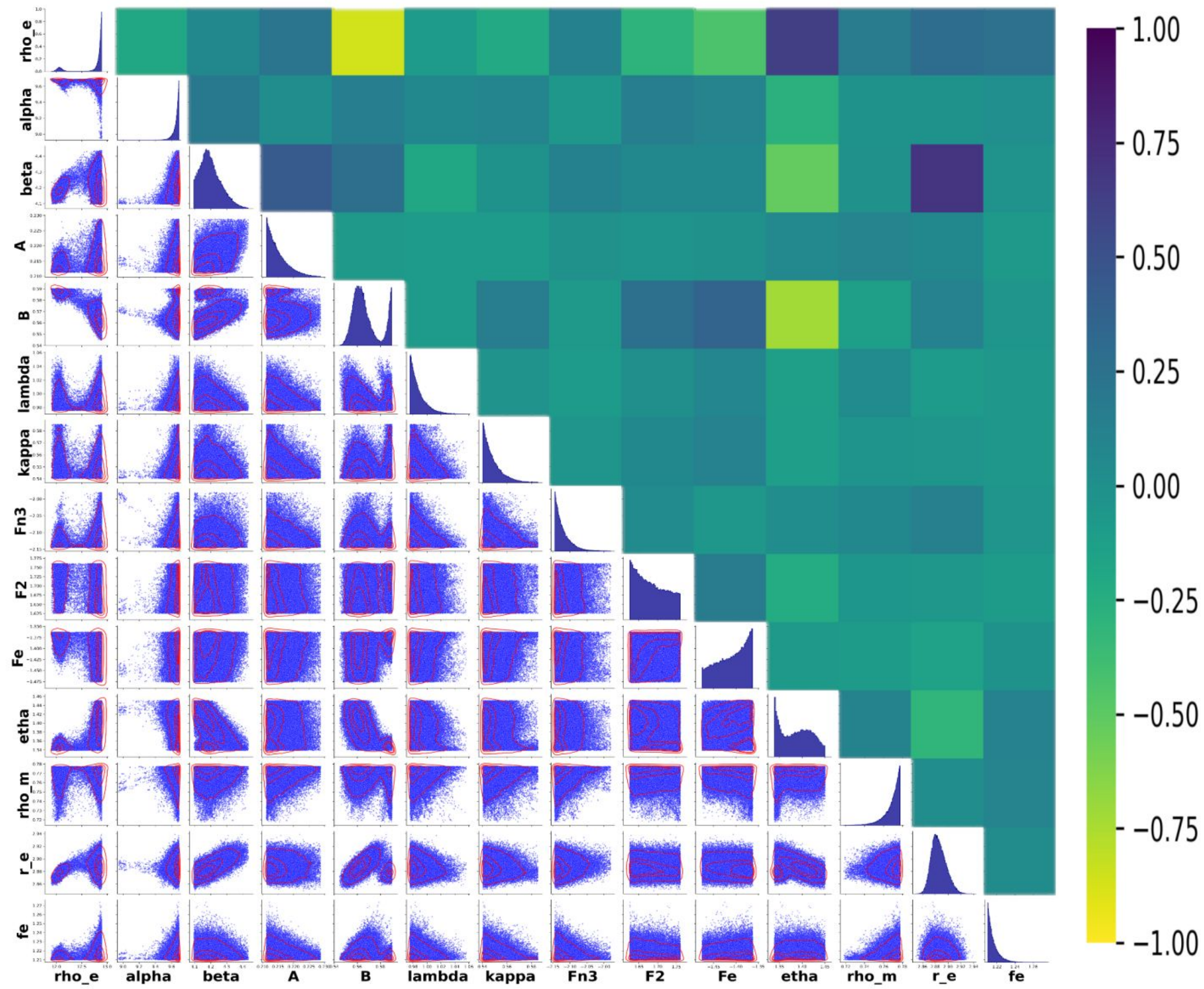

Figure S30: Corner plot for all parameters with AIES sampler.

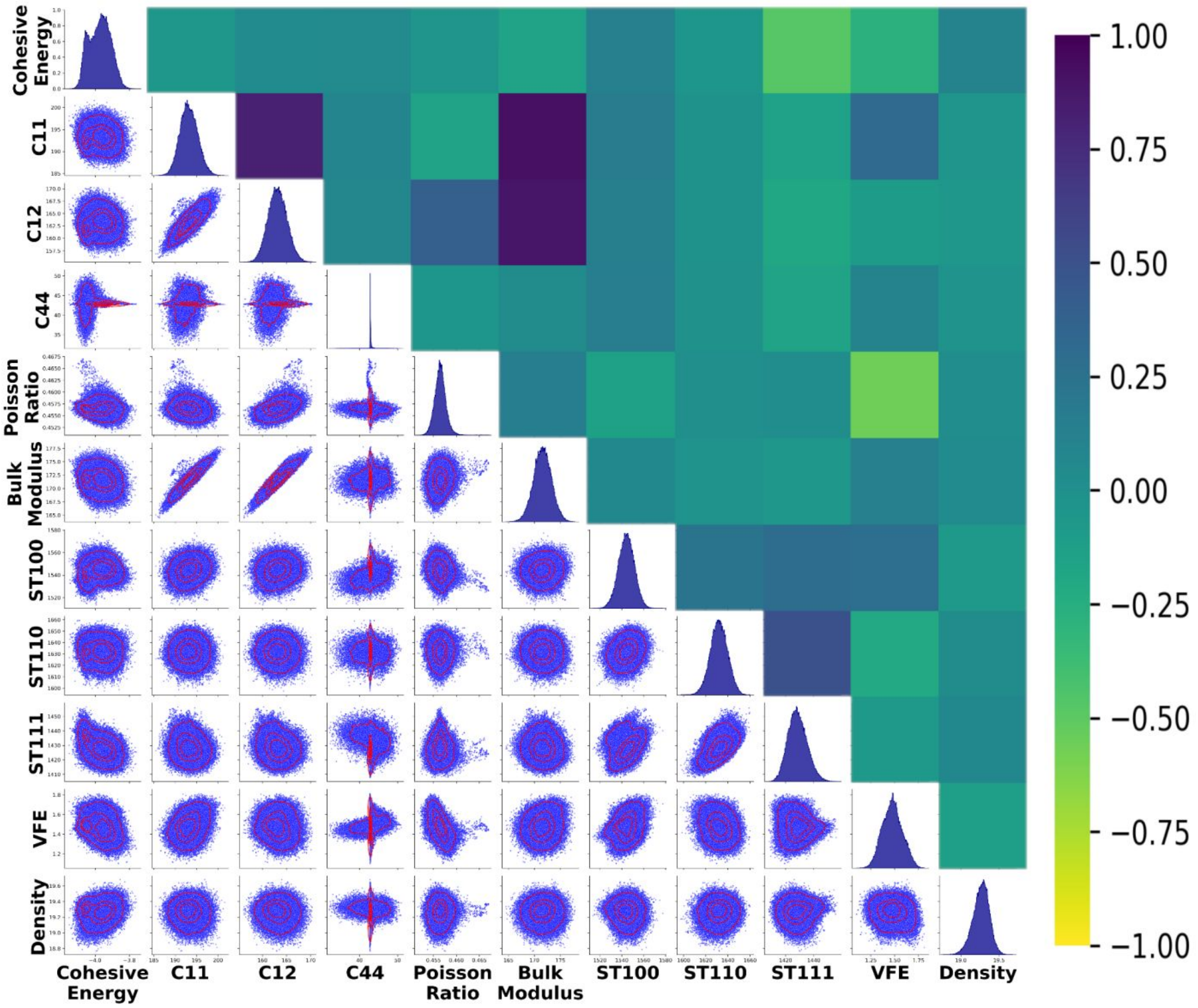

Figure S31: Corner plot for all properties with AIES sampler.

III) 95% Credible Intervals

Table S6. A comparison of values defining 95 % credible intervals for parameters obtained from ESS sampler.

	ESS					
	Mode 1			Mode 2		
	MAP	mu_AP		MAP	mu_AP	
		mean	std_dev		mean	2 std_dev
**rhoe**	12.893	12.845	0.117	11.973	12.081	0.17

**alpha**	9.664	9.65	0.086	9.691	9.67	0.041
**beta**	4.149	4.201	0.137	4.162	4.19	0.075
**A**	0.212	0.215	0.007	0.211	0.214	0.005
**B**	0.56	0.563	0.013	0.59	0.587	0.005
**lamda**	0.976	0.987	0.023	0.98	0.992	0.027
**kappa**	0.542	0.548	0.014	0.553	0.553	0.021
**Fn3**	-2.14	-2.119	0.05	-2.139	-2.127	0.034
**F2**	1.638	1.679	0.076	1.746	1.708	0.074
**Fe**	-1.462	-1.416	0.064	-1.39	-1.378	0.028
**etha**	1.4	1.397	0.056	1.339	1.347	0.013
**rhom**	0.777	0.769	0.016	0.777	0.764	0.024
**Lc**	4.084	4.083	0.032	4.066	4.071	0.019
**fe**	1.214	1.217	0.013	1.21	1.213	0.006

Table S7. A comparison of values defining 95 % credible intervals for parameters obtained from AIES sampler.

	**AIES**					
	**Mode 1**			**Mode 2**		
	**MAP**	**mu_AP**		**MAP**	**mu_AP**	
		**mean**	**2 std_dev**		**mean**	**2 std_dev**
**rhoe**	12.891	12.845	0.115	12.018	12.084	0.174
**alpha**	9.687	9.65	0.098	9.687	9.671	0.04
**beta**	4.16	4.203	0.138	4.182	4.189	0.074
**A**	0.212	0.215	0.007	0.212	0.214	0.004
**B**	0.559	0.563	0.013	0.59	0.587	0.005
**lamda**	0.977	0.987	0.023	0.977	0.99	0.026
**kappa**	0.543	0.548	0.014	0.542	0.552	0.021
**Fn3**	-2.144	-2.118	0.052	-2.144	-2.126	0.038
**F2**	1.633	1.678	0.075	1.743	1.708	0.073

**Fe**	-1.413	-1.416	0.063	-1.363	-1.38	0.031
**etha**	1.421	1.397	0.057	1.354	1.347	0.014
**rhom**	0.776	0.769	0.015	0.773	0.766	0.023
**Lc**	4.083	4.083	0.033	4.071	4.071	0.019
**fe**	1.212	1.217	0.013	1.211	1.213	0.006

Table S8. A comparison of values defining 95 % credible intervals for parameters obtained from MH, URS, and GS sampler.

	**MH**			**URS**			**GS**		
	**MAP**	**mu_AP**		**MAP**	**mu_AP**		**MAP**	**mu_AP**	
		**mean**	**2 std_dev**		**mean**	**2 std_dev**		**mean**	**2 std_dev**
**rhoe**	12.881	12.846	0.11	12.813	12.635	0.427	12.571	12.506	0.205
**alpha**	9.672	9.65	0.097	9.166	9.411	0.406	9.448	9.366	0.194
**beta**	4.117	4.201	0.134	4.284	4.22	0.178	4.207	4.237	0.081
**A**	0.212	0.215	0.007	0.216	0.22	0.01	0.217	0.22	0.005
**B**	0.557	0.563	0.013	0.588	0.575	0.022	0.575	0.571	0.011
**lamda**	0.977	0.988	0.023	1.001	1.015	0.047	1.002	1.016	0.023
**kappa**	0.55	0.549	0.014	0.578	0.563	0.026	0.555	0.563	0.013
**Fn3**	-2.138	-2.118	0.056	-2.075	-2.065	0.095	-2.091	-2.063	0.047
**F2**	1.644	1.68	0.078	1.629	1.694	0.078	1.67	1.693	0.038
**Fe**	-1.439	-1.417	0.065	-1.419	-1.417	0.066	-1.399	-1.418	0.032
**etha**	1.44	1.397	0.059	1.411	1.388	0.062	1.375	1.392	0.031
**rhom**	0.776	0.769	0.016	0.744	0.748	0.034	0.758	0.748	0.017
**Lc**	4.074	4.083	0.034	4.173	4.063	0.092	4.052	4.021	0.063
**fe**	1.214	1.218	0.013	1.22	1.239	0.045	1.243	1.25	0.022

In order to validate these parameter sets, top 100 samples were randomly chosen from each mode from each sampler**.** The most probable sample i.e. maximum a posteriori (MAP) from each sampler's MD results are tabulated in **Table S9-S11**.

Table S9. A comparison of values defining 95 % credible intervals for properties obtained from ESS algorithm.

	**ESS**					
	**Mode 1**			**Mode 2**		
	MAP	mu_AP		MAP	mu_AP	
		mean	std_dev		mean	std_dev
**Cohesive Energy**	-4.017	-3.955	0.105	-4.088	-4.048	0.047
**C11**	191.654	193.346	4.185	191.156	192.542	3.552
**C12**	162.956	163.413	3.893	160.849	161.844	2.986
**C44**	42.783	42.895	0.495	38.476	42.455	6.437
**Poisson's Ratio**	0.457	0.457	0.003	0.457	0.456	0.001
**Bulk_modulus**	170.272	171.511	3.556	170.833	171.648	2.968
**ST100**	1551.883	1545.383	13.002	1541.307	1537.344	13.482
**ST110**	1624.265	1632.127	15.639	1624.391	1630.235	15.245
**ST111**	1436.7	1426.904	11.353	1446.102	1435.098	12.012
**Density**	19.243	19.256	0.216	19.353	19.311	0.132
**VFE**	1.431	1.47	0.221	1.485	1.485	0.104

Table S10. A comparison of values defining 95 % credible intervals for properties obtained from AIES algorithm.

	**AIES**					
	**Mode 1**			**Mode 2**		
	MAP	mu_AP		MAP	mu_AP	
		mean	std_dev		mean	std_dev

**Cohesive Energy**	-3.955	-3.955	0.105	-4.088	-4.051	0.048
**C11**	192.547	193.371	4.166	191.156	192.524	3.436
**C12**	163.698	163.414	3.833	160.849	161.878	2.895
**C44**	42.776	42.892	0.466	38.476	42.618	6.348
**Poisson's Ratio**	0.457	0.457	0.003	0.457	0.456	0.001
**Bulk_modulus**	171.153	171.531	3.514	170.833	171.662	2.864
**ST100**	1556.495	1545.442	13.197	1541.307	1538.237	13.454
**ST110**	1620.242	1632.098	15.664	1624.391	1630.989	14.37
**ST111**	1435.619	1426.903	11.433	1446.102	1435.95	11.908
**Density**	19.23	19.254	0.22	19.353	19.316	0.131
**VFE**	1.436	1.474	0.223	1.485	1.485	0.105

Table S11. A comparison of values defining 95 % credible intervals for properties obtained from MH, URS, and GS algorithms.

	**MH**			**URS**			**GS**		
	MAP	mu_AP		MAP	mu_AP		MAP	mu_AP	
		mean	std_dev		mean	std_dev		mean	std_dev
**Cohesive Energy**	-3.966	-3.956	0.11	-3.953	-3.971	0.171	-3.969	-3.911	0.082
**C11**	192.531	193.372	4.2	192.913	196.813	15.899	194.269	198.345	10.733
**C12**	164.772	163.431	3.844	164.248	167.222	14.315	164.445	169.703	9.414
**C44**	42.799	42.888	0.473	48.14	44.311	4.526	49.585	46.598	3.027
**Poisson's Ratio**	0.458	0.457	0.003	0.458	0.459	0.006	0.458	0.461	0.003
**Bulk_modulus**	171.651	171.529	3.59	171.381	176.079	14.263	172.713	178.316	9.487

**ST100**	1545.53	1545.134	12.941	1549.961	1499.014	67.349	1512.382	1458.183	48.418
**ST110**	1618.379	1632.52	15.66	1666.455	1648.28	69.88	1649.686	1623.47	53.573
**ST111**	1426.702	1426.697	11.353	1450.187	1392.202	56.289	1418.094	1357.513	42.311
**Density**	19.194	19.256	0.221	19.403	19.372	0.965	19.514	19.701	0.803
**VFE**	1.308	1.469	0.228	1.371	1.343	0.376	1.375	1.171	0.196

The coefficient of variation (CoV) for priors (the data from sobol sequence) and posterior distributions of each sampler are shown in **Table S12**.

Table S12: A comparison of Coefficient of Variation (CoV) between Sobol Sequence Data and Bayesian UQ.

**Properties**	**Sobol Variation %**	**Bayesian UQ Variation %**						
		**ESS**		**AIES**		**MH**	**URS**	**GS**
		Mode 1	Mode 2	Mode 1	Mode 2			
**Cohesive Energy**	1.838	1.325	0.583	1.331	0.59	1.39	2.153	1.048
**C11**	5.35	1.082	0.922	1.077	0.892	1.086	4.039	2.706
**C12**	5.321	1.191	0.922	1.173	0.894	1.176	4.28	2.774
**C44**	7.635	0.577	7.581	0.544	7.447	0.551	5.107	3.248
**Poisson's Ratio**	0.446	0.288	0.148	0.295	0.149	0.328	0.654	0.325
**Bulk Modulus**	5.316	1.037	0.865	1.024	0.834	1.046	4.05	2.66
**ST100**	4.856	0.421	0.438	0.427	0.437	0.419	2.246	1.66
**ST110**	4.728	0.479	0.468	0.48	0.441	0.48	2.12	1.65
**ST111**	5.84	0.398	0.419	0.401	0.415	0.398	2.022	1.558
**VFE**	15.764	0.561	0.342	0.572	0.34	0.574	2.491	2.038
**Density**	4.211	7.525	3.496	7.549	3.529	7.76	13.999	8.369

Table S13. The comparison of computational resources and time used for PSO and BUQ.

Computational Method	Number of total samples	Physical time (in hrs)	Number of cores used	Total core hours
PSO	12,800	60-70	256	~17,000
BUQ	2,000,000	40-60	12	~500-700

IV) Bayesian UQ Posterior Analysis

(i) Validation of GP predictions compared with MD

We selected 100 random parameter sets from each posterior mode, and these parameters were further validated by performing MD simulations for 1 ns at ~298 K for systems with 8000 atoms and properties were calculated for the last 10 ps as they remained unchanged after initial equilibration of 100 ps. We observed excellent agreement between MD and GP predictions for all properties except C44. The better alignment of cobalt-blue and orange-colored graphs confirms that indeed the 95 % credible intervals predicted by ESS, AIES, and MH were accurate compared to URS and GS. While those predicted by ESS and AIES were narrower compared to MH. As mentioned previously, the C44 surrogate model exhibited high variability (Sobol standard deviation (σ) = 7.6%), despite the overall accuracy of the model. The property C44 showed a persistent deviation among all the samplers, with MD predictions falling around 49 GPa, a considerable 15 % higher than the GPR predictions that hover around the target value of 42.3 GPa. This discrepancy was consistent and notable, and may be attributable to the inherent variability observed in the GP model, as well as the sensitivity of the C44 property reflected in the Sobol sequence data. The evaluation of all GP predictions and MD simulations on a chosen set of 100 posterior distribution parameters is depicted in **Fig. S33-S42**. Likelihood is also shown to represent target and variation in them denoted by green color. Here, MD priors and GP priors are shown in cobalt-blue and red colored graphs, whereas MD posterior and GP posterior points are shown in orange and dark blue colors respectively.

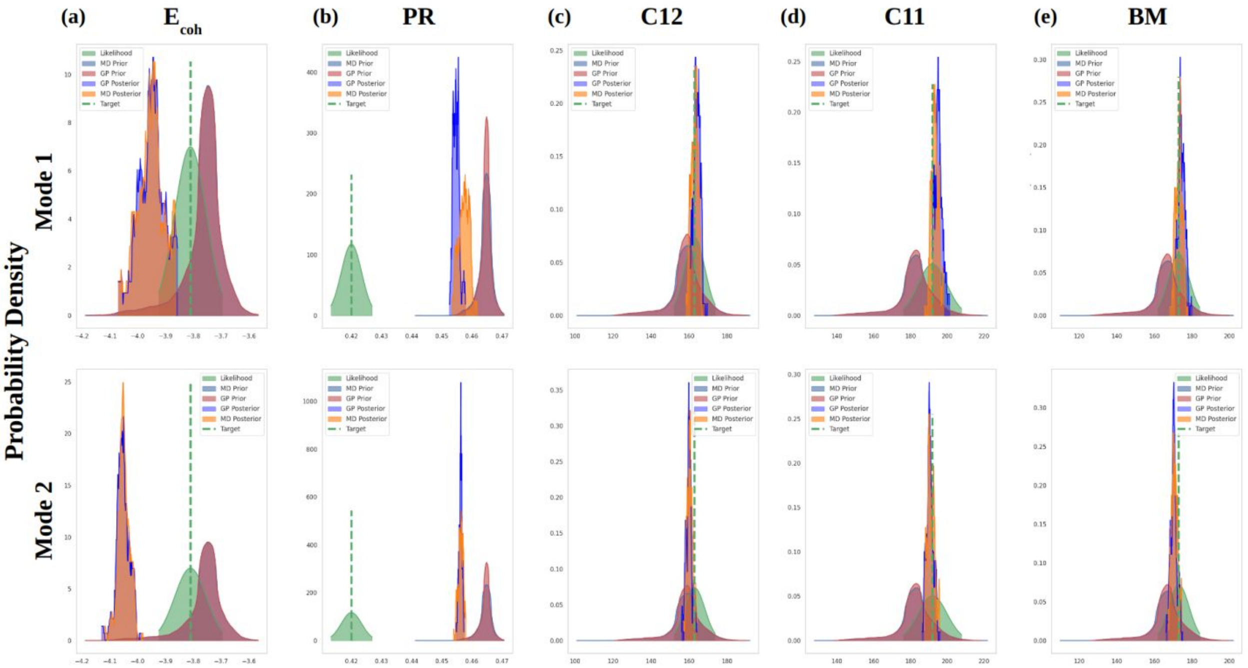

Figure S32. The comparison of GP predictions with the MD validation of prior points, and posterior points obtained from AIES sampler (first 5 properties).

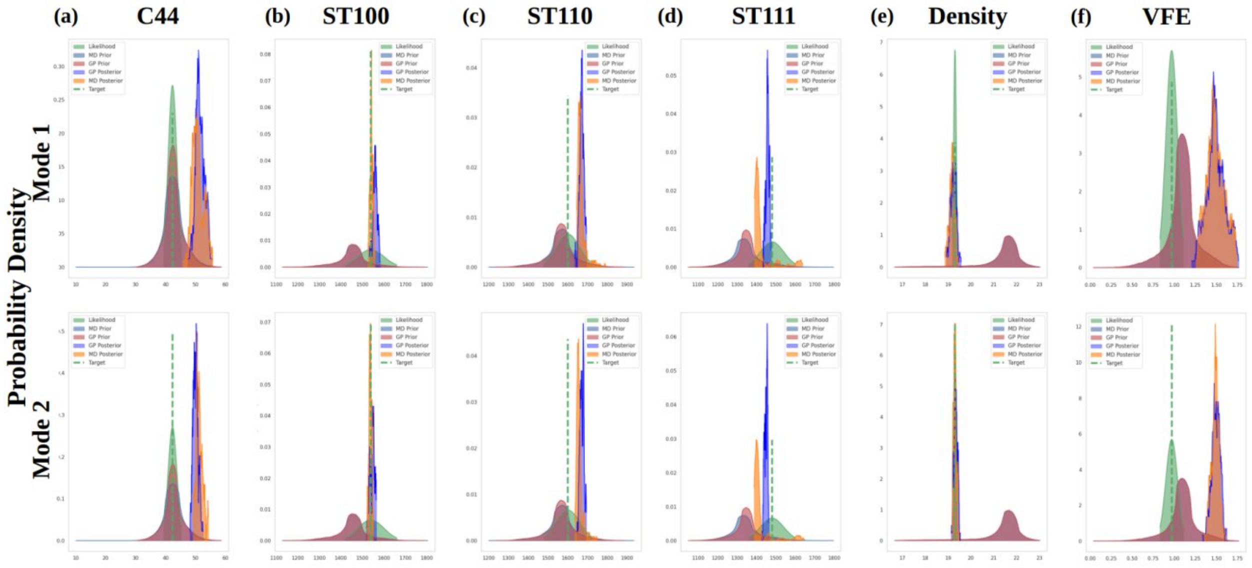

Figure S33. The comparison of GP predictions with the MD validation of prior points, and posterior points obtained from AIES sampler (last 6 properties).

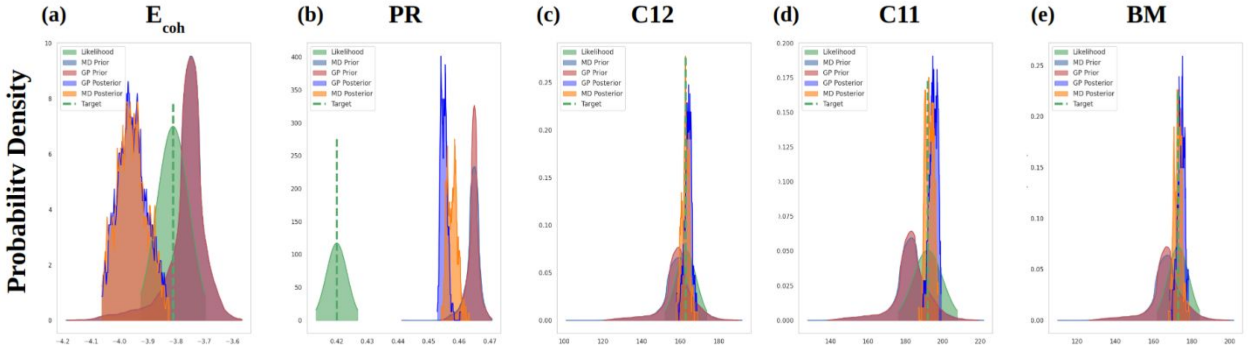

Figure S34. The comparison of GP predictions with the MD validation of prior points, and posterior points obtained from MH sampler (first 5 properties).

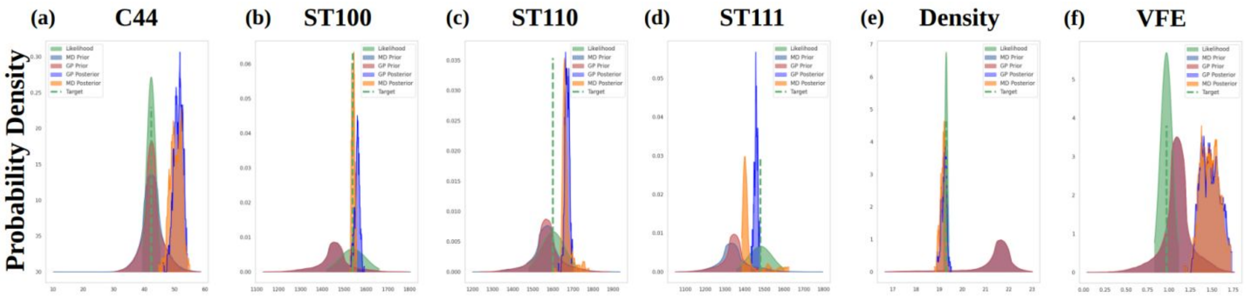

Figure S35. The comparison of GP predictions with the MD validation of prior points, and posterior points obtained from MH sampler (last 6 properties).

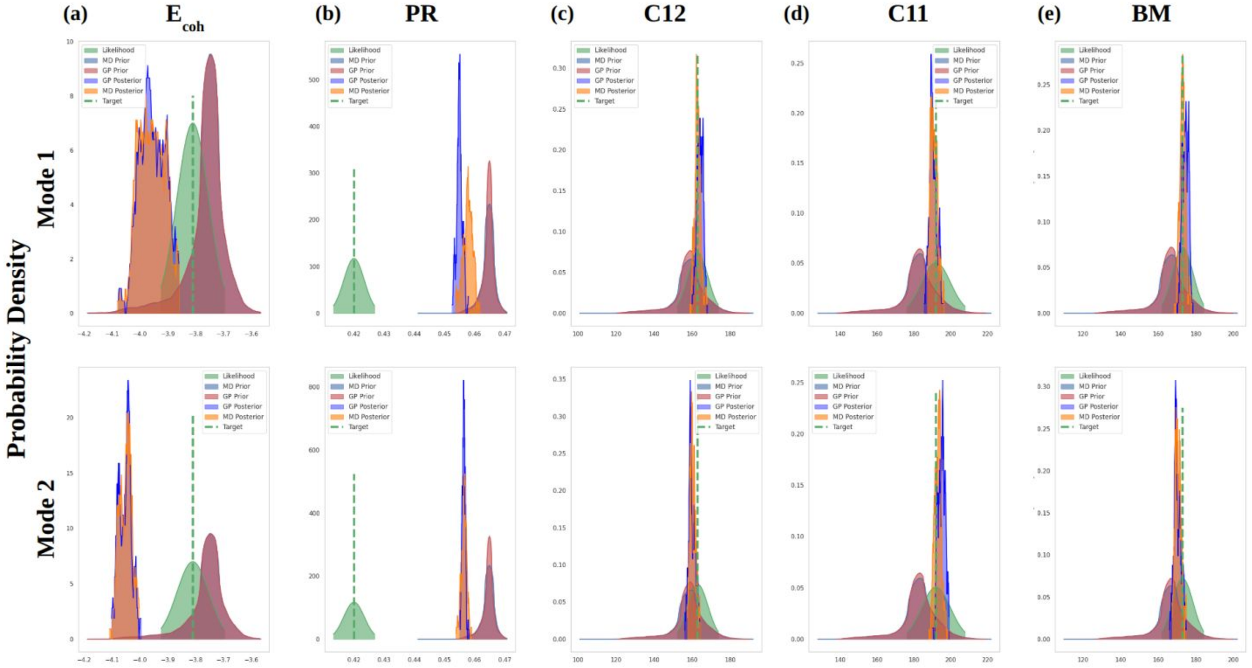

Figure S36. The comparison of GP predictions with the MD validation of prior points, and posterior points obtained from ESS sampler (first 5 properties).

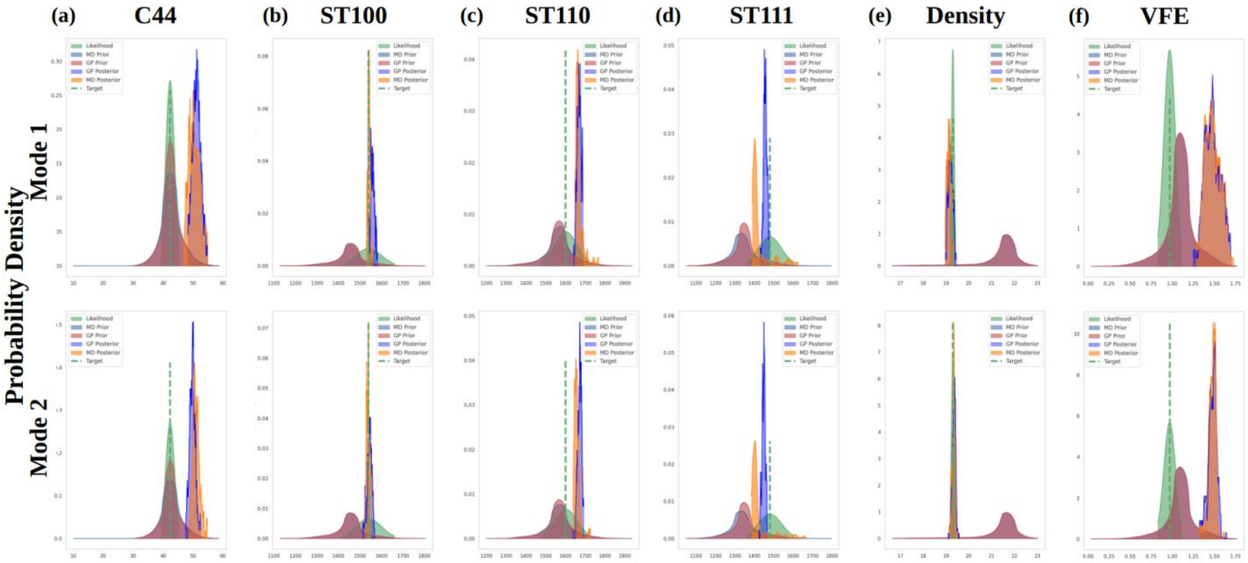

Figure S37. The comparison of GP predictions with the MD validation of prior points, and posterior points obtained from ESS sampler (last 6 properties).

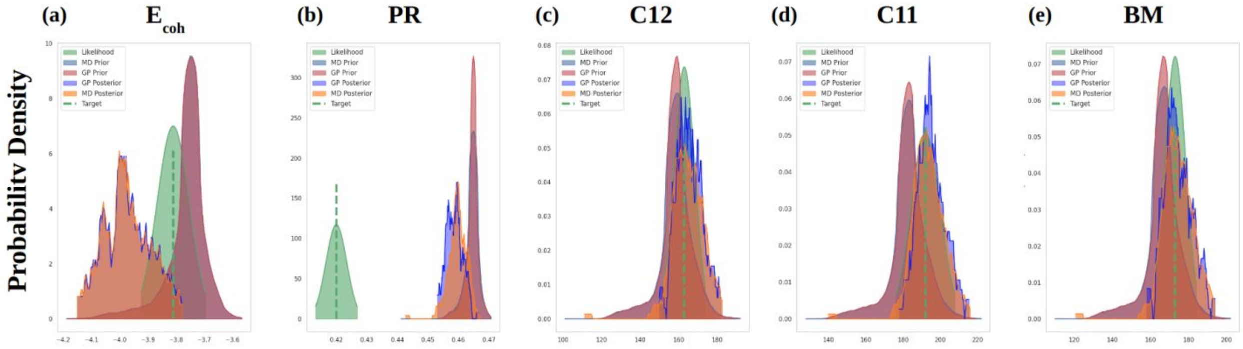

Figure S38. The comparison of GP predictions with the MD validation of prior points, and posterior points obtained from URS sampler (first 5 properties).

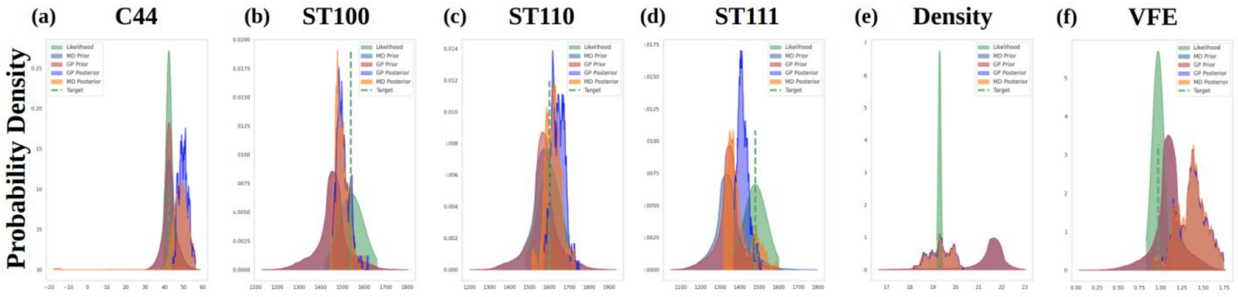

Figure S39. The comparison of GP predictions with the MD validation of prior points, and posterior points obtained from URS sampler (last 6 properties).

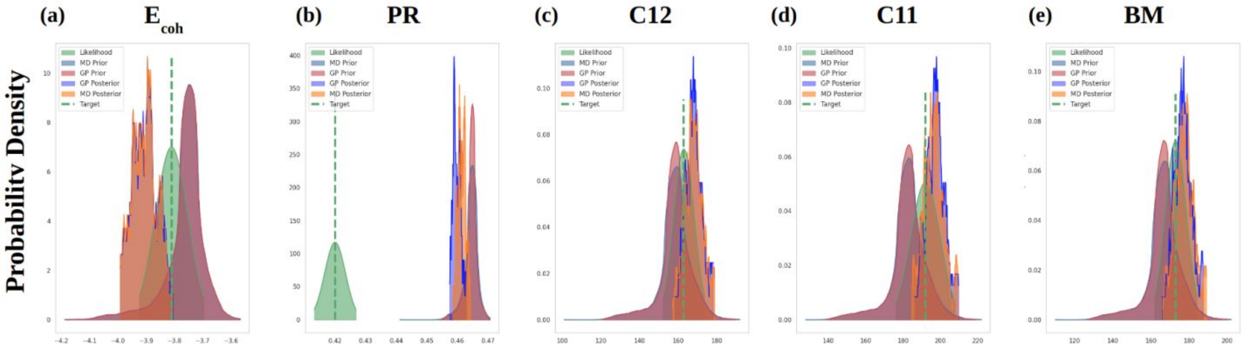

Figure S40. The comparison of GP predictions with the MD validation of prior points, and posterior points obtained from GS sampler (first 5 properties).

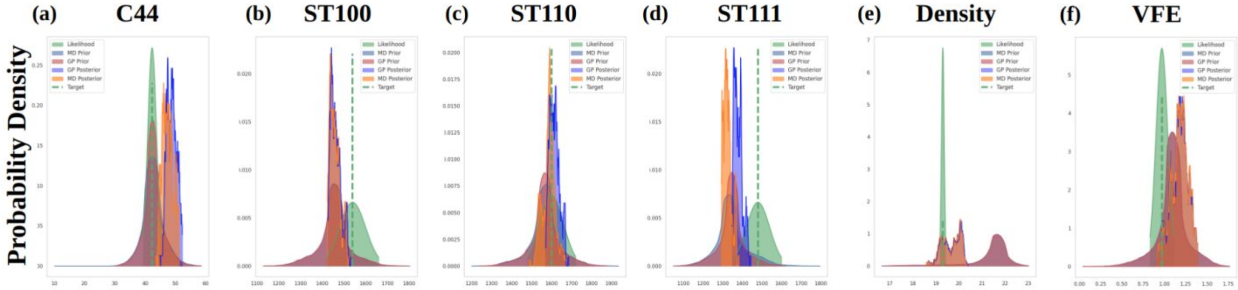

Figure S41. The comparison of GP predictions with the MD validation of prior points, and posterior points obtained from GS sampler (last 6 properties).

(ii) Posterior Predictive Plots

Posterior predictive plots illustrate the properties of MD calculations derived from samples drawn from the posterior distribution of Bayesian UQ outcomes. In the context of Bayesian UQ, our objective is to observe a rise in probability, denoted by the posterior distribution converging towards the likelihood distribution (i.e., experimental observations). To achieve this, the algorithm may need to compromise certain properties in favor of an overall increase in probability.

(i) Cohesive

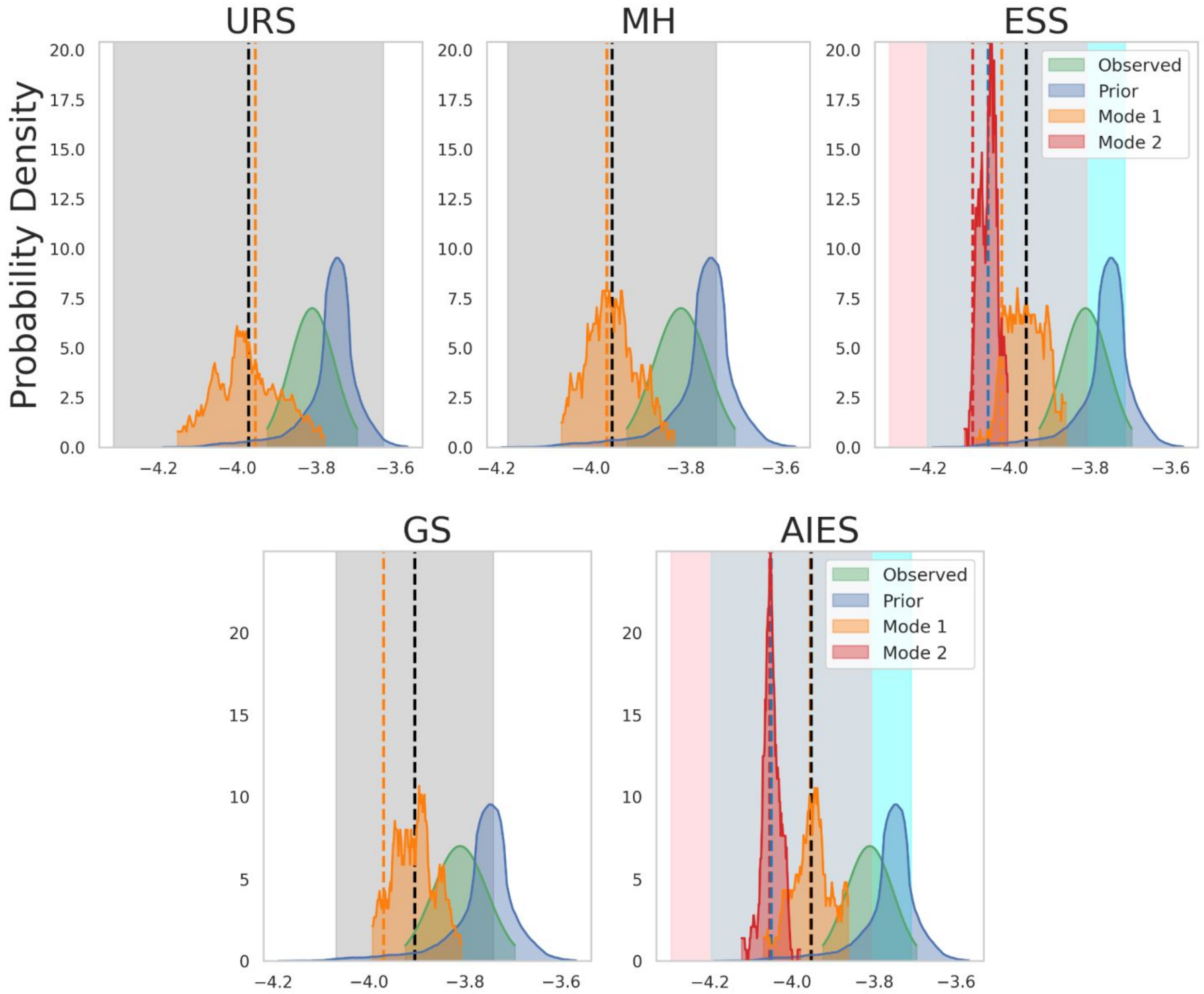

Figure S42: The comparison plot for the target likelihood, priors compared with the MD simulations on the posterior distribution from respective samplers and their 95 % credible intervals for cohesive energy.

(ii) Poisson Ratio

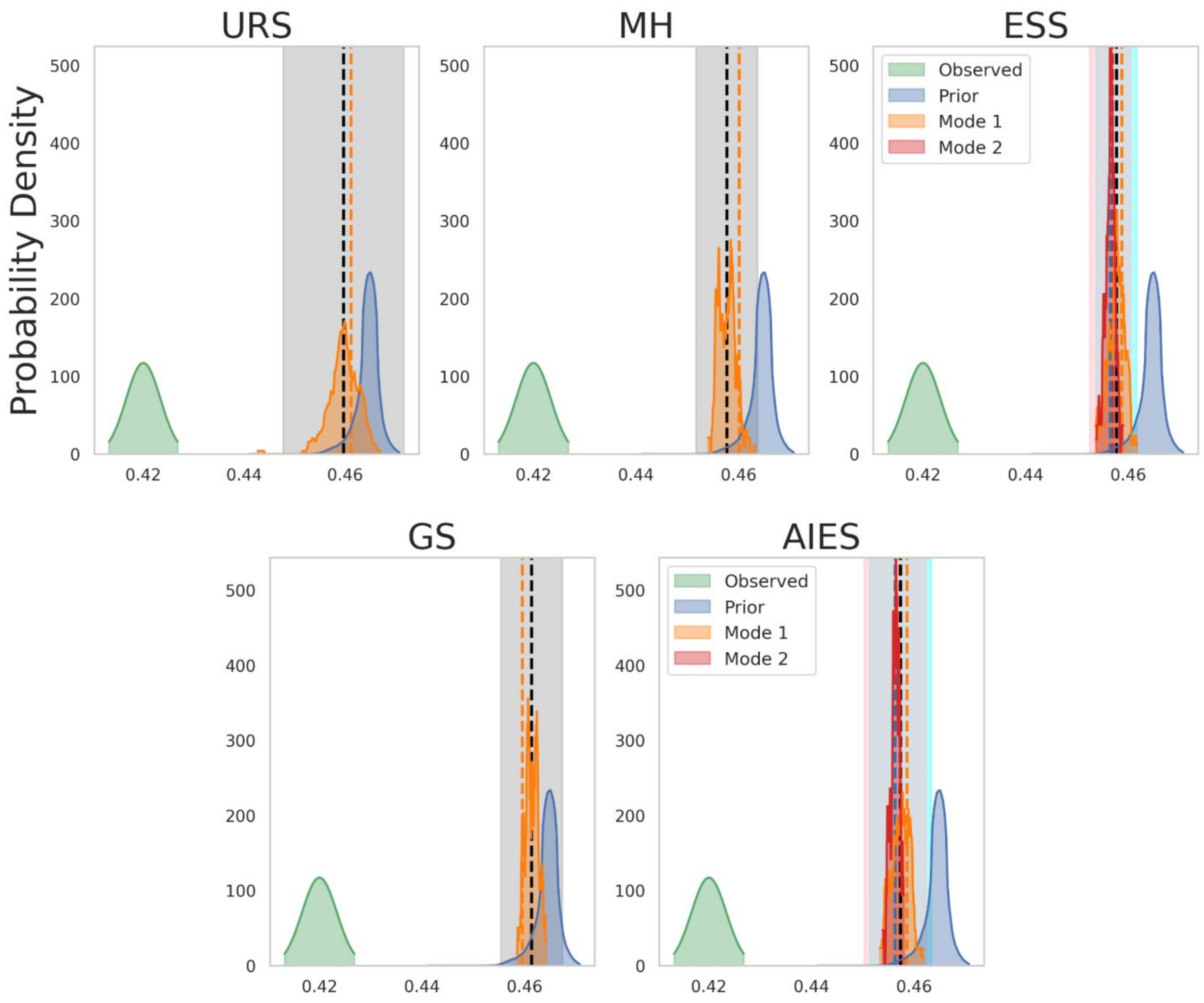

Figure S43: The comparison plot for the target likelihood, priors compared with the MD simulations on the posterior distribution from respective samplers and their 95 % credible intervals for poisson ratio.

(iii) C12

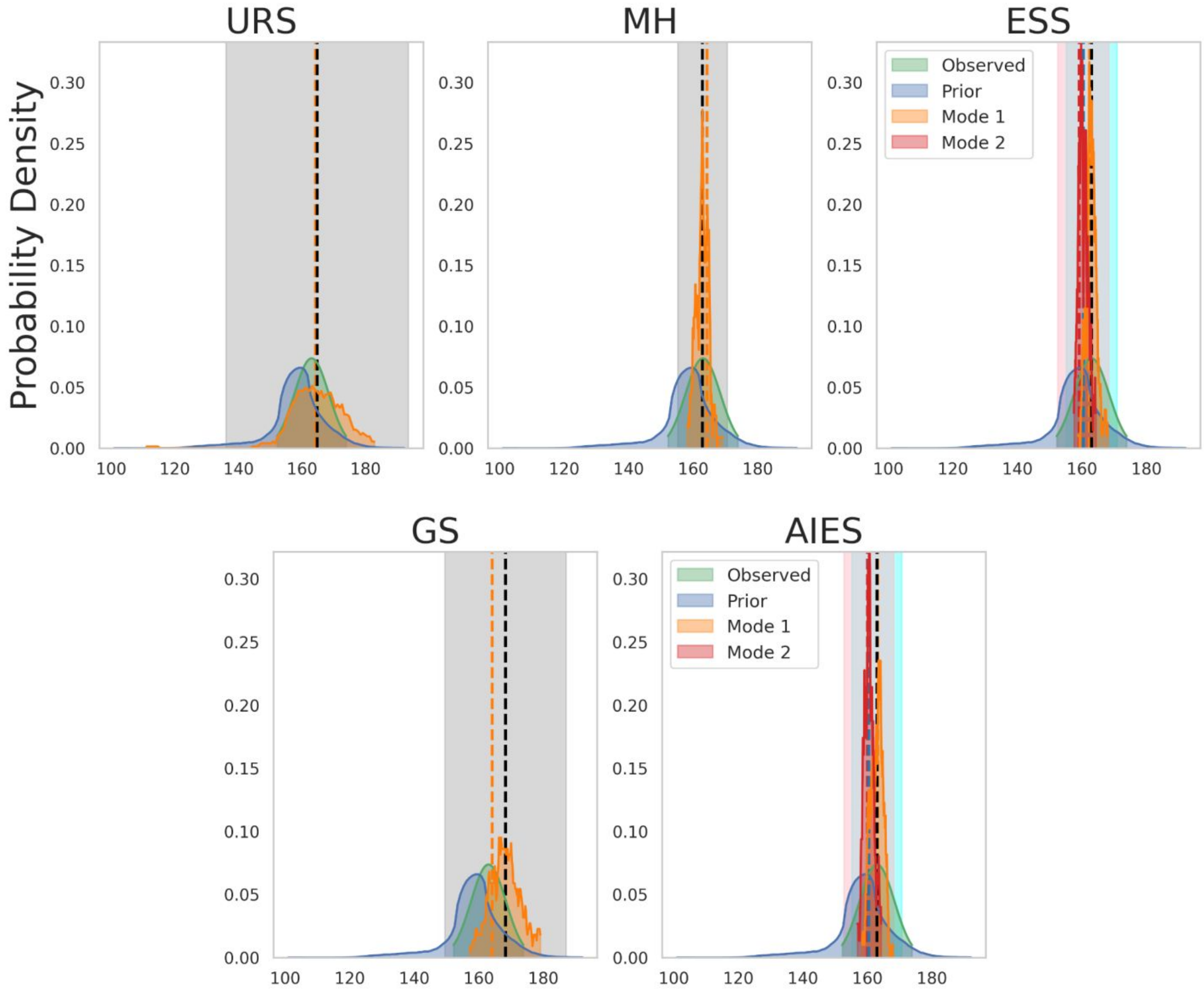

Figure S44: The comparison plot for the target likelihood, priors compared with the MD simulations on the posterior distribution from respective samplers and their 95 % credible intervals for C12.

(iv) C11

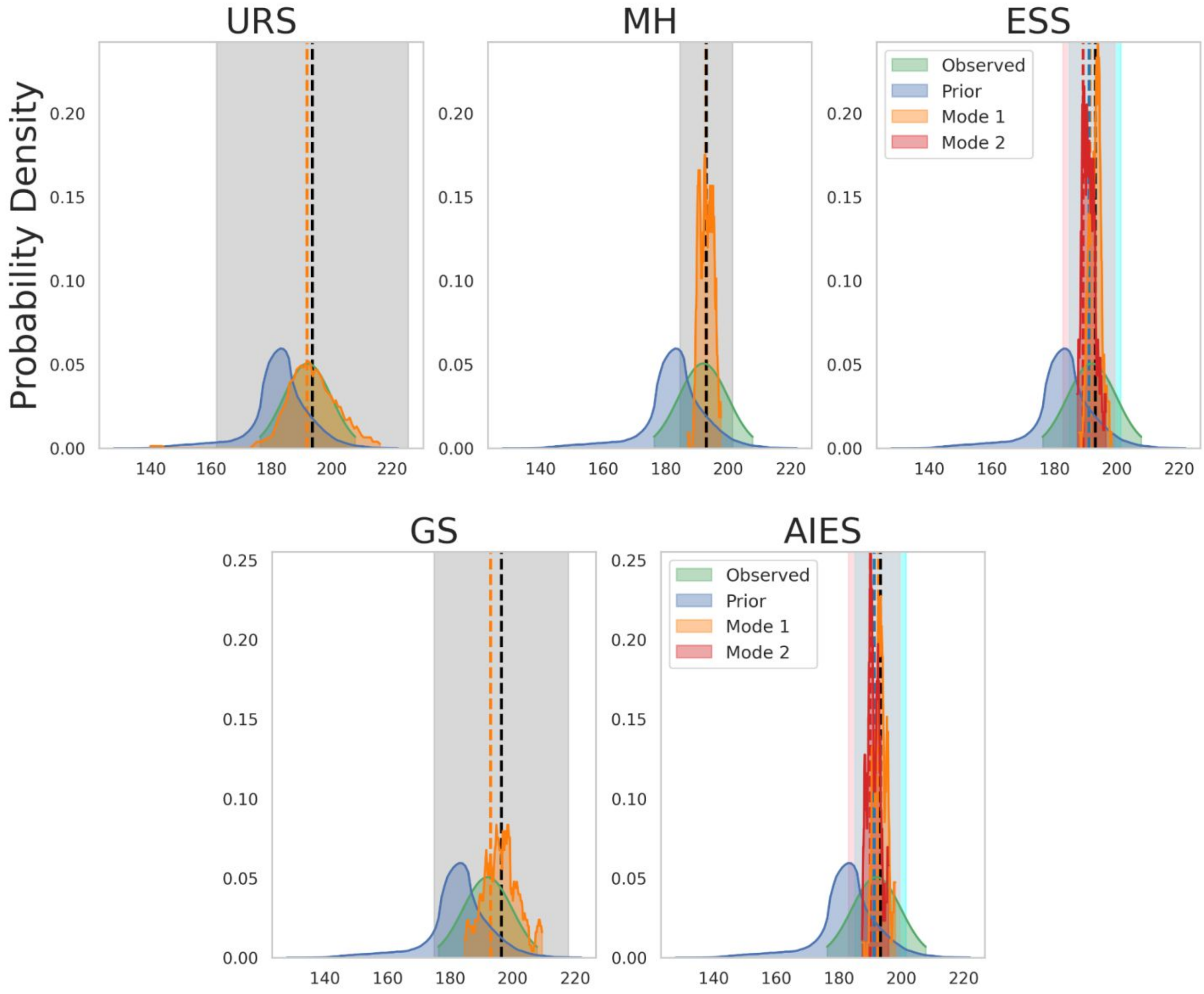

Figure S45: The comparison plot for the target likelihood, priors compared with the MD simulations on the posterior distribution from respective samplers and their 95 % credible intervals for C11.

(v) Bulk modulus

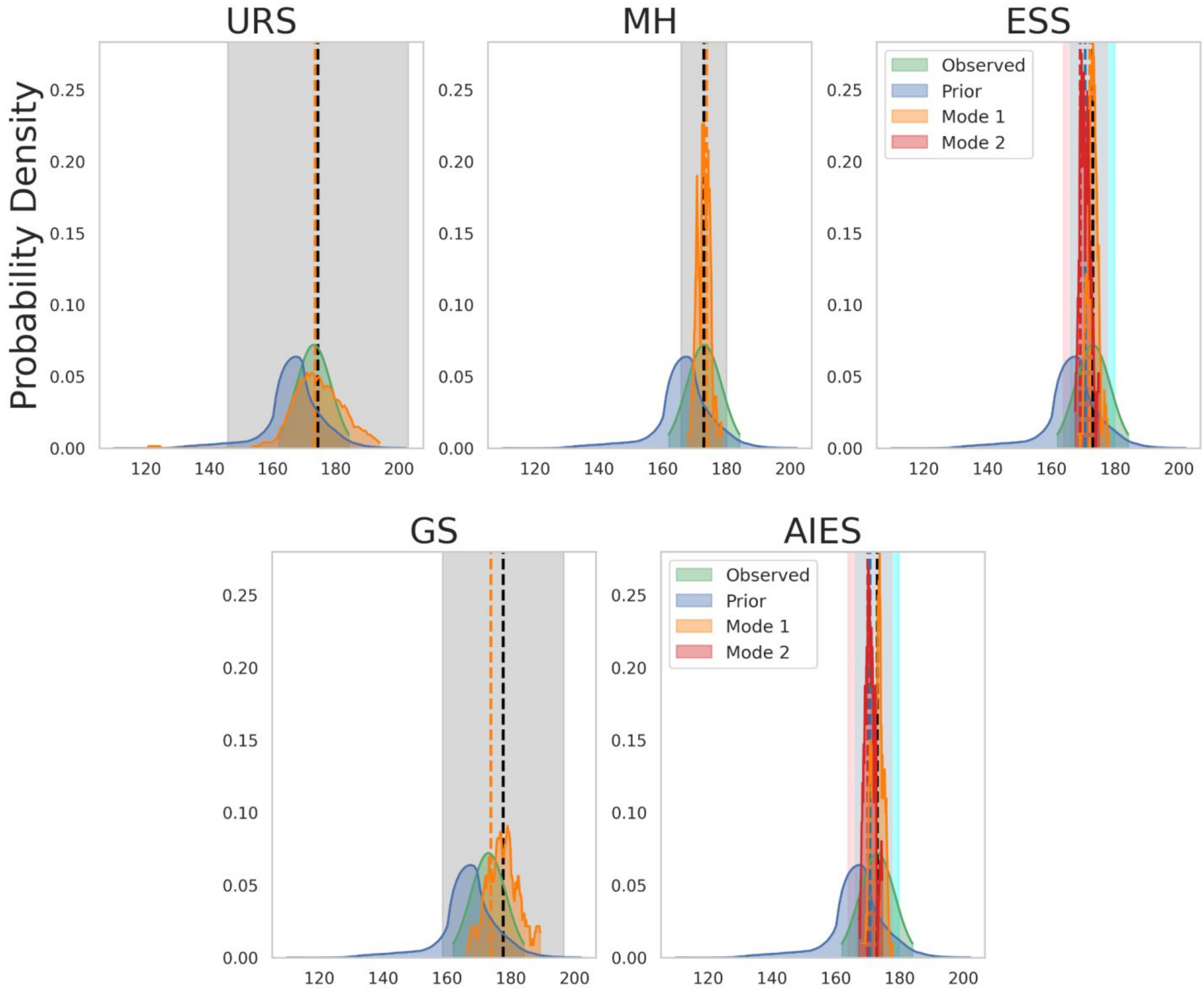

Figure S46: The comparison plot for the target likelihood, priors compared with the MD simulations on the posterior distribution from respective samplers and their 95 % credible intervals for bulk modulus.

(vi) C44

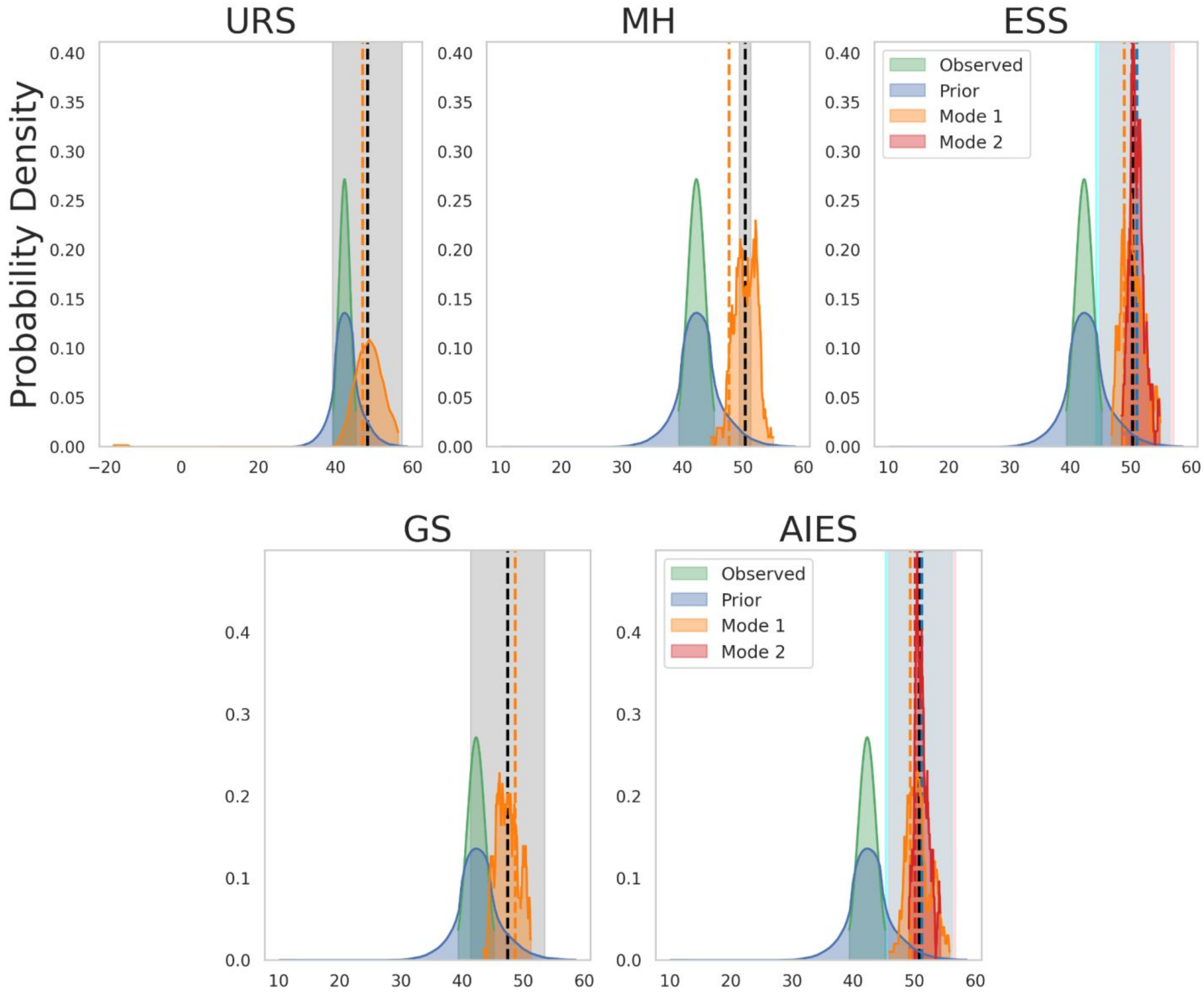

Figure S47: The comparison plot for the target likelihood, priors compared with the MD simulations on the posterior distribution from respective samplers and their 95 % credible intervals for C44.

(vii) ST 100

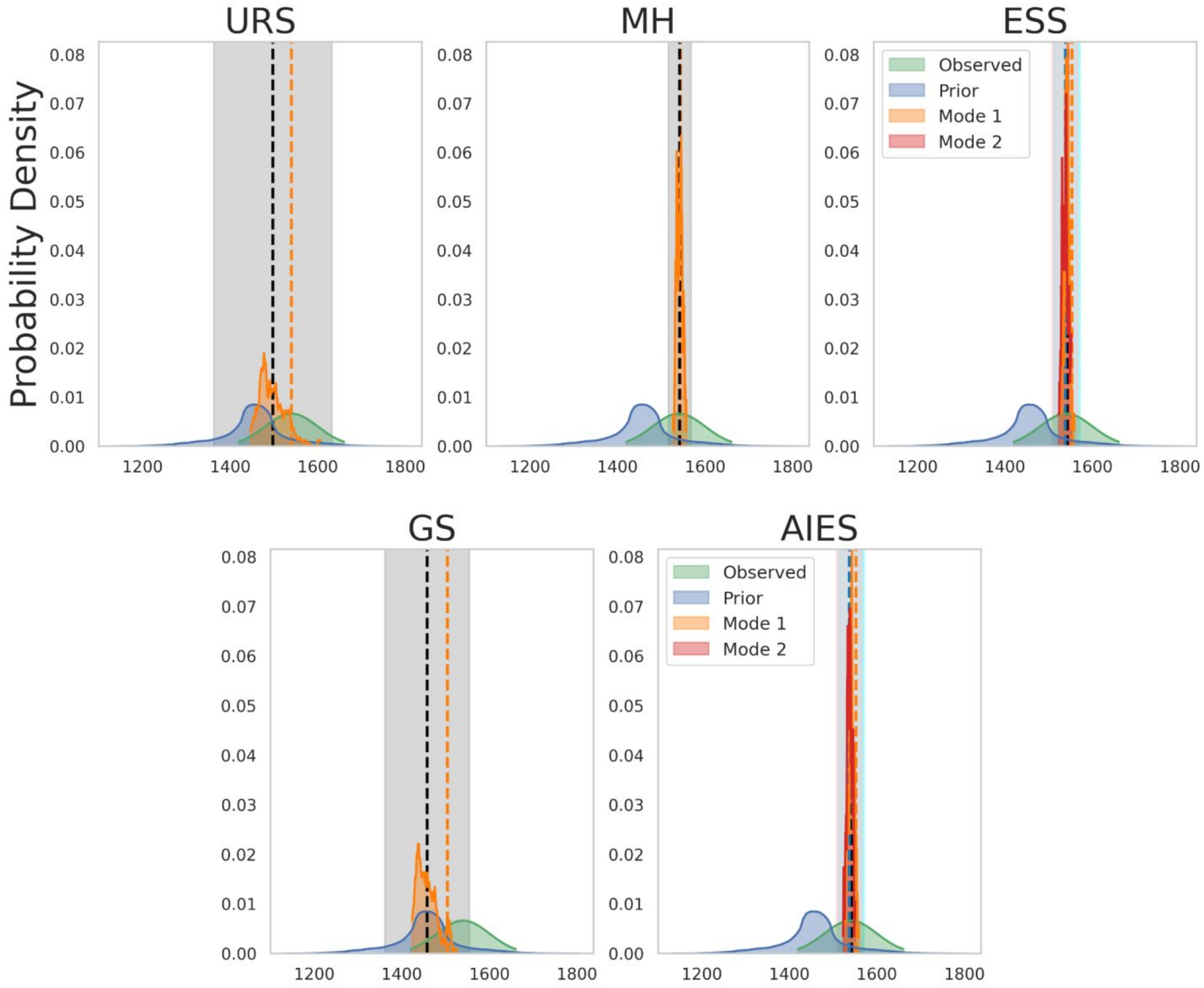

Figure S48: The comparison plot for the target likelihood, priors compared with the MD simulations on the posterior distribution from respective samplers and their 95 % credible intervals for surface tension at 100 surface.

(viii) ST 110

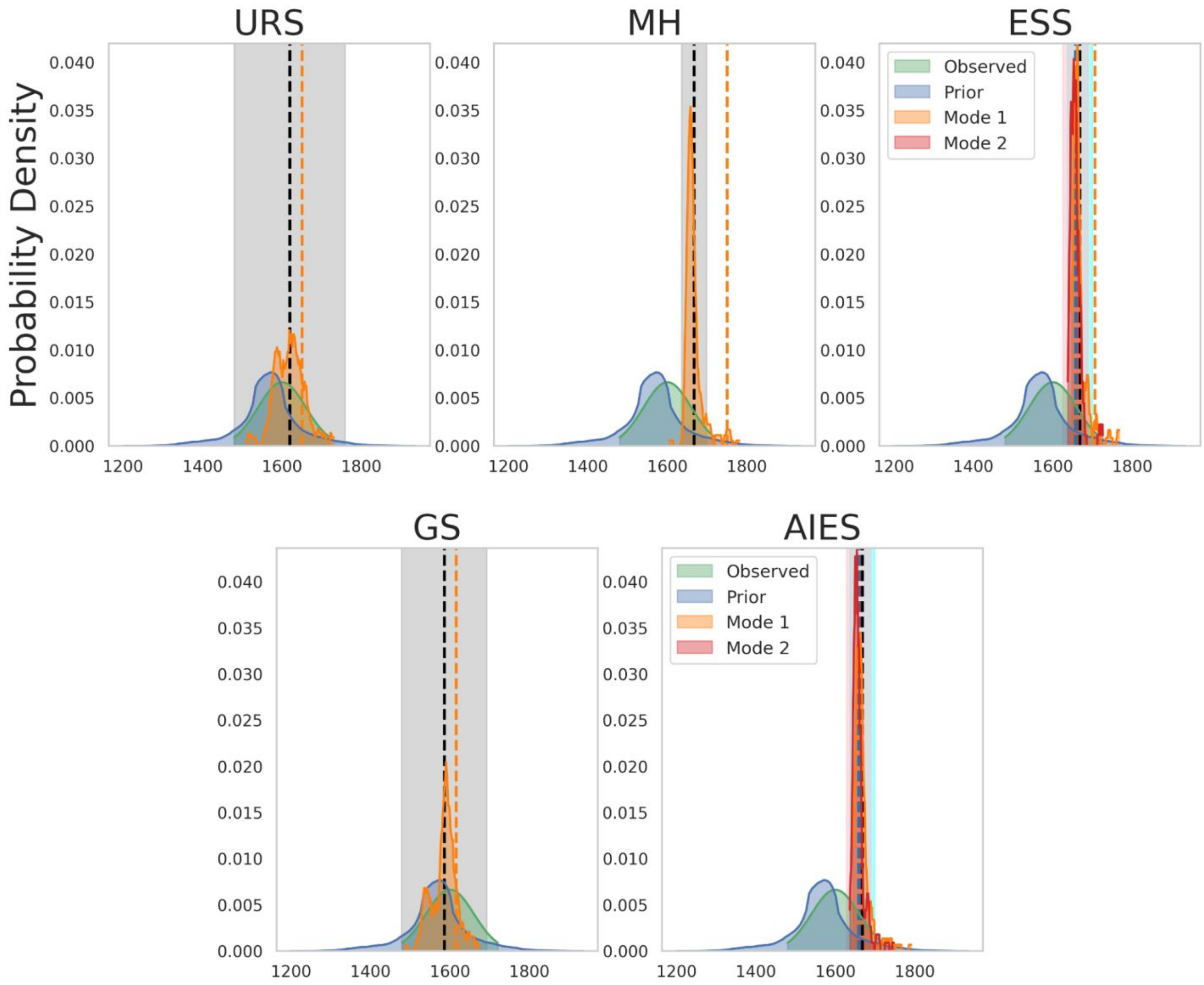

Figure S49: The comparison plot for the target likelihood, priors compared with the MD simulations on the posterior distribution from respective samplers and their 95 % credible intervals for surface tension at 110 surface.

(ix) ST 111

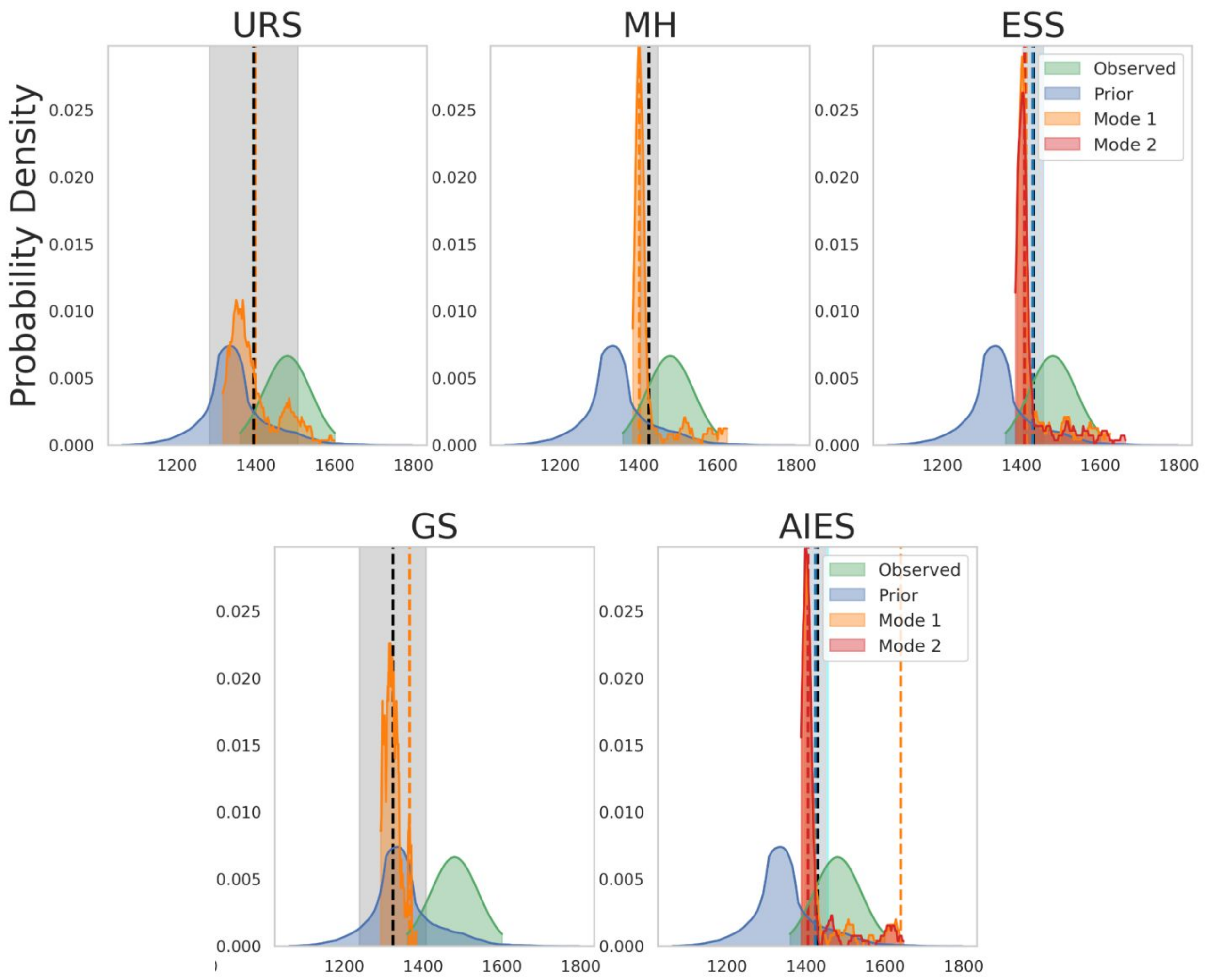

Figure S50: The comparison plot for the target likelihood, priors compared with the MD simulations on the posterior distribution from respective samplers and their 95 % credible intervals for surface tension at 111 surface.

(x) Density

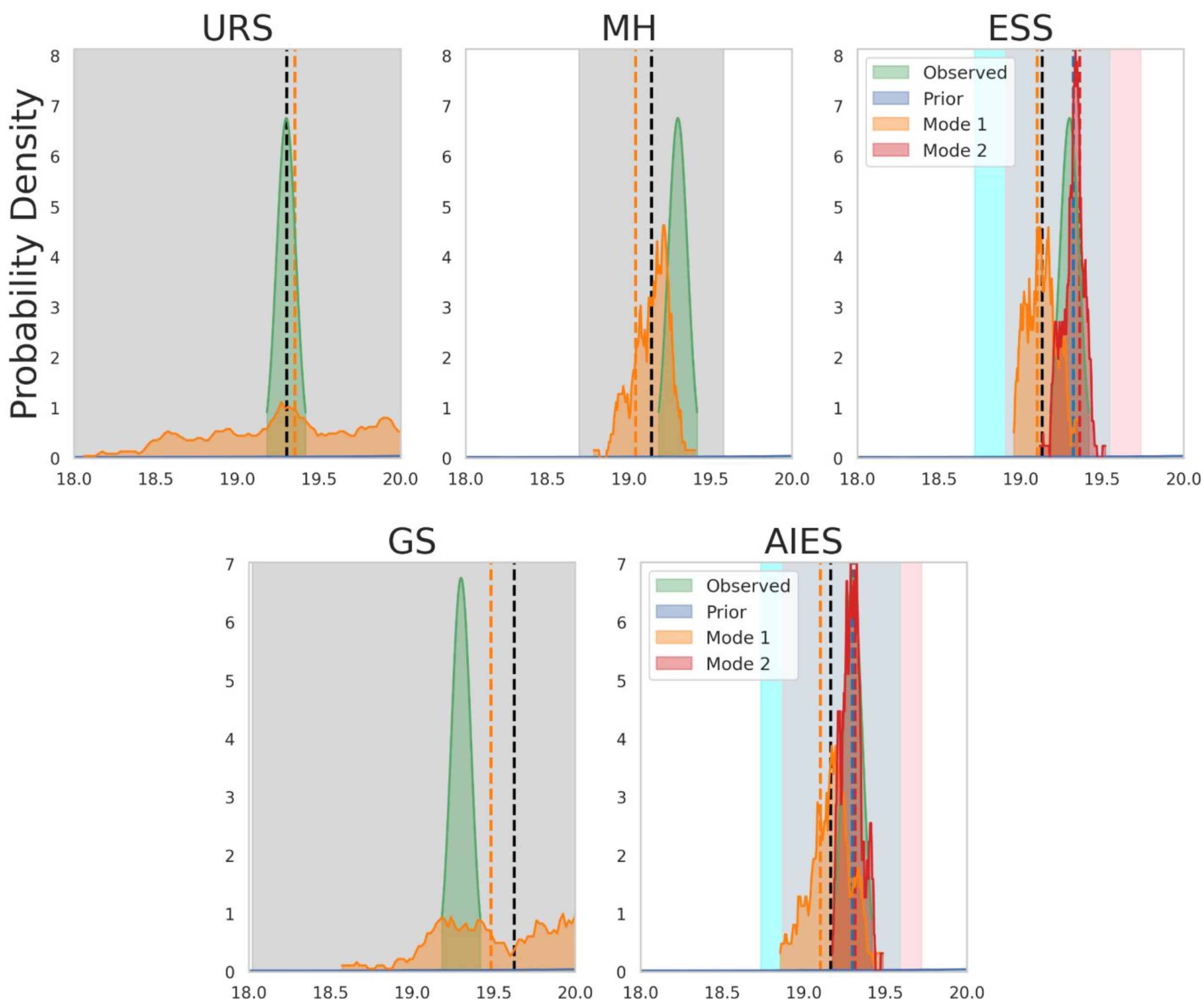

Figure S51: The comparison plot for the target likelihood, priors compared with the MD simulations on the posterior distribution from respective samplers and their 95 % credible intervals for density.

(xi) VFE

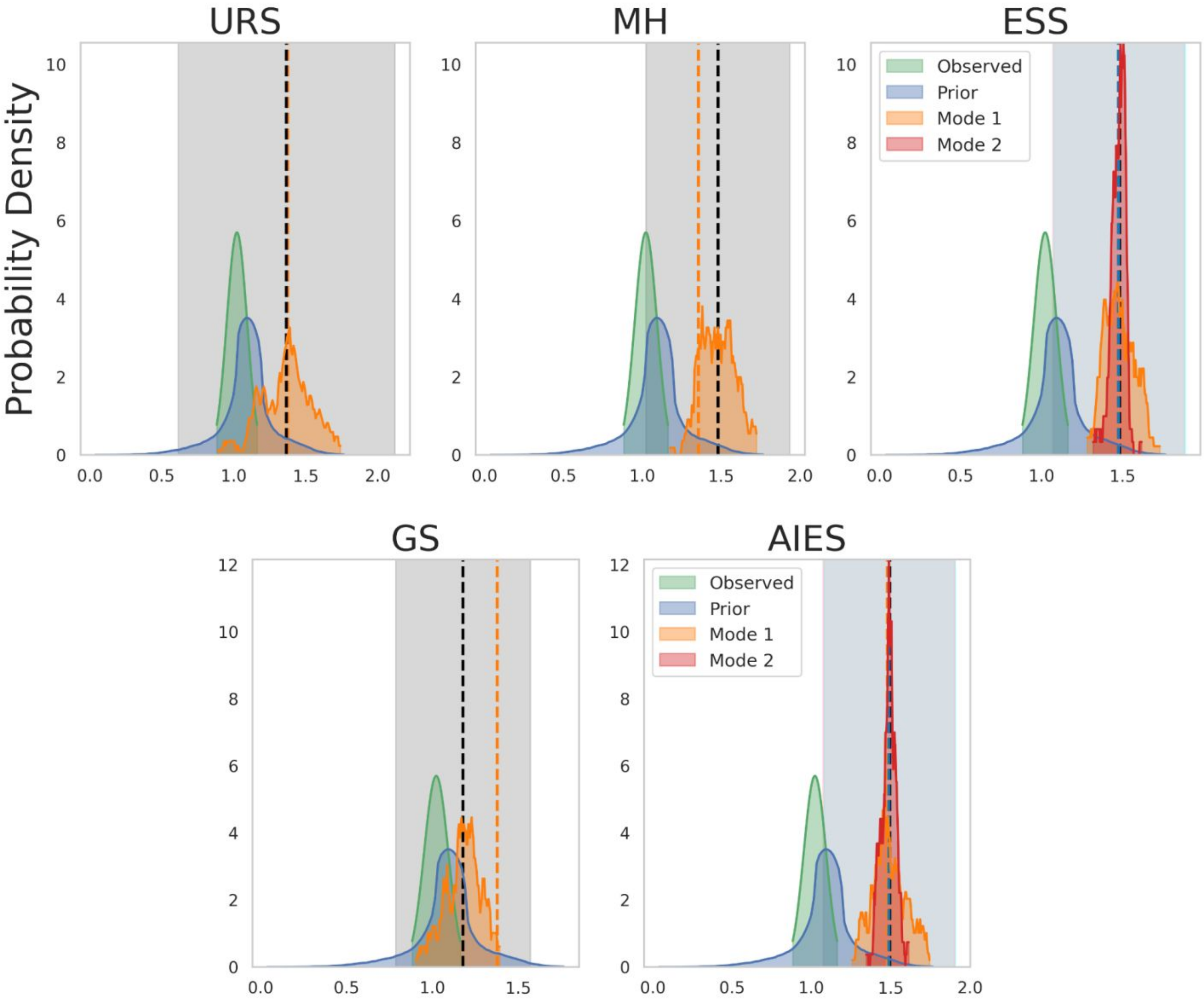

Figure S52: The comparison plot for the target likelihood, priors compared with the MD simulations on the posterior distribution from respective samplers and their 95 % credible intervals for VFE.

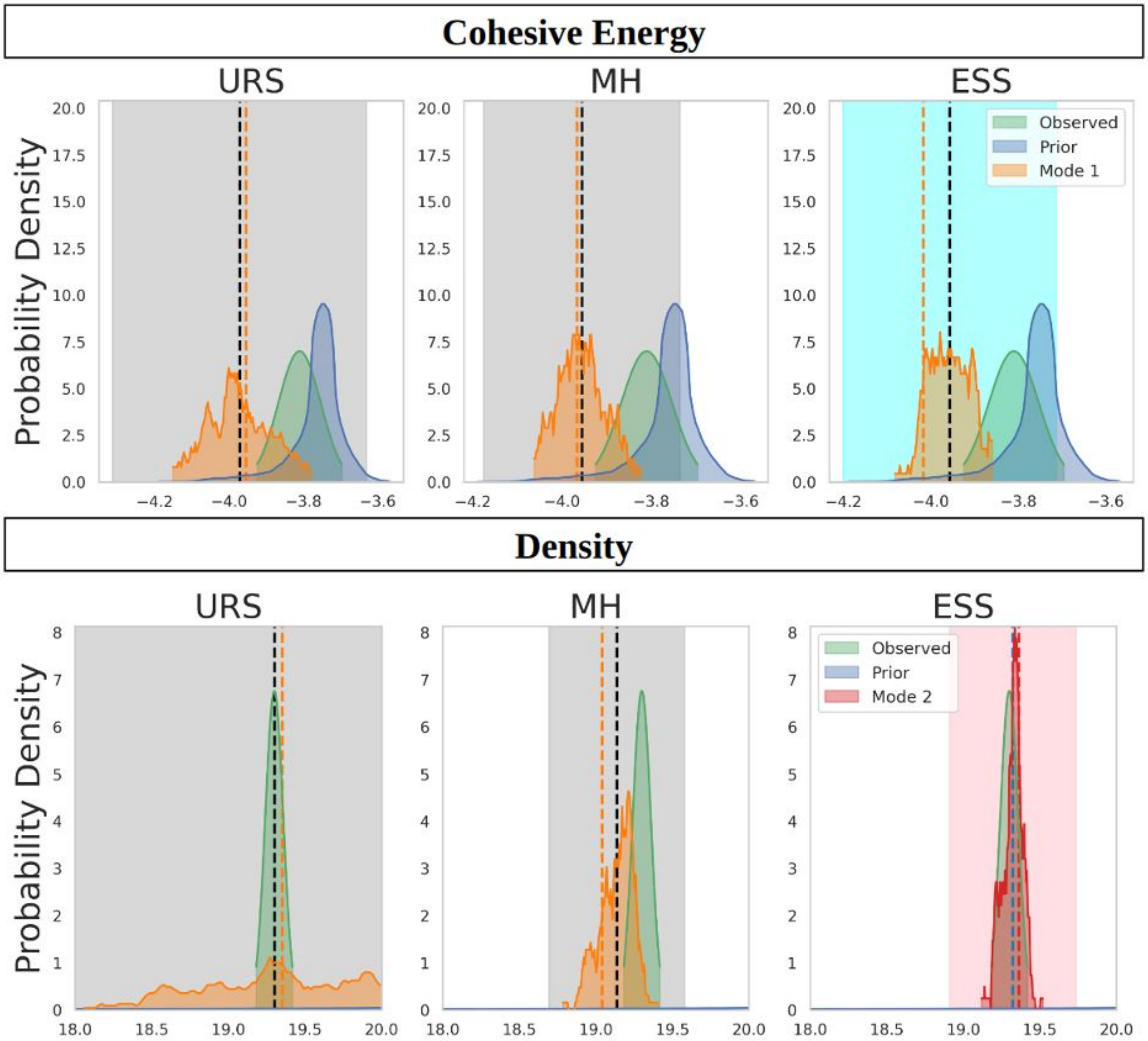

Figure S53. MD simulations runs on 100 random samples from mode 1 and mode 2 of posterior distribution identified by ESS, AIES, and MH samplers for E_{coh} ((a), (b), and (c)) and density ((d), (e), and (f)). The green, blue, orange, and red colored graphs represent the likelihood function, prior (from Sobol data), and Mode 1 and Mode 2 of the posterior distribution. The density plots are zoomed on the likelihood distribution which cuts most of the prior distribution. The local MAP for mode 1 is displayed as an orange dotted line, and the local MAP for mode 2 is depicted by a red dotted line (only detected by ESS). The black and blue dashed lines show the values of the mean of each sampler's posterior distribution, which is their μ_{AP}. The 95 % credible interval is shown around μ_{AP} with gray/cyan/red shaded graphs.

A visual comparison of the two modes from ESS (**Fig. S42** of **SI)**, for cohesive energy and density (**Fig. S51** of **SI**) suggests better alignment of posterior distribution for Mode 1 (orange) with experimental target distributions for cohesive energy compared to Mode 2 distribution. In contrast, we observed a better agreement of prediction by Mode 2 with targets for density as compared to Mode 1. It can be seen that bulk modulus, C11, C12, and C44 showed a similar behavior to cohesive energy (**Fig. S44-S47** of **SI**), where Mode 1 favored these mechanical properties. On the other hand, surface tension graphs (**Fig. S48-S50** of **SI**) were similar to density where Mode 2 favored these physical properties. The mechanical properties were more accurately predicted when the emphasis was placed on the embedding energy, while density predictions were more accurate when the emphasis was placed on pairwise energy at the first nearest neighbors. To facilitate a direct comparison, predictions from the GP on the priors are also included.

In addition to the detection of the two modes observed, the top 100 parameters predicted from posterior distribution after ESS exhibited a reduction in the overall error compared to PSO determined values. In the case of density the most probable 100 samples from ESS reduced the error by estimating local MAP values, which were closer to target than the PSO determined values. Except for E_{coh} and C44, the errors of all properties were reduced from ~5 % variation to ≤ ~1 % demonstrating the proficiency of BPE for efficient optimization of multi-parameter space problems. Seven out of nine properties demonstrated good agreement of the posterior distribution with the experimental likelihood distribution, when looking at the best mode for that property. However, distributions of the E_{coh} and C44 were displaced further away, which is a result of the BUQ approach seeking the most probable solutions which involve tradeoffs in accuracy between properties.

Section S3) Physical Insights

From **Fig. 2** of the main manuscript, Mode 1 was characterized by superior mechanical properties influenced by many-body effects in the EAM potential, governing bonding and coordination number. On the other hand, Mode 2 demonstrated superior predictive ability for density, relying on short-range interactions. The examination of energy functions confirmed a trade-off between embedding and pairwise contributions, leading to more accurate mechanical properties for Mode 1 and superior accuracy for physical property prediction like density for Mode 2. Interestingly, one of the posterior peaks, Mode 1 which resulted in better match for E_{coh} was found by all the MCMC samplers (MH, ESS, and AIES). However, the second posterior peak Mode 2, which has a high magnitude and low variance, was only found by ESS and AIES. This demonstrates the usefulness of these newer samplers, which more thoroughly explored the posterior.

Using gen.eam files generated using the parameters from the two modes, we plot the contributions of two terms from **equation SE1** in **Fig. S54.** This trade-off of the contribution of the embedding and pairwise partitions to the total energy in the EAM potential becomes evident when examining the energy functions as shown in **Fig. S54**. For the case of Mode 1, the embedding energy is a significantly strong contributor, but the corresponding energy at the first nearest neighbor *i.e.* at distance = 2.82 Å (i.e. -0.351 eV) is weaker (or less negative) as compared to mode 2 (-0.380 eV at 2.79 Å) and sobol (-0.340 eV at 2.67 Å) as shown in **Fig. S54. (a)**. Consequently, this results in more accurate mechanical properties. In contrast, Mode 2 has weaker embedding energy for all electron densities as shown in **Fig. S54 (b)** while maintaining stronger energy at the first nearest neighbor (-0.380 eV), as shown in **Fig. S54 (a),** through the pairwise interaction, resulting in a more accurate density prediction.

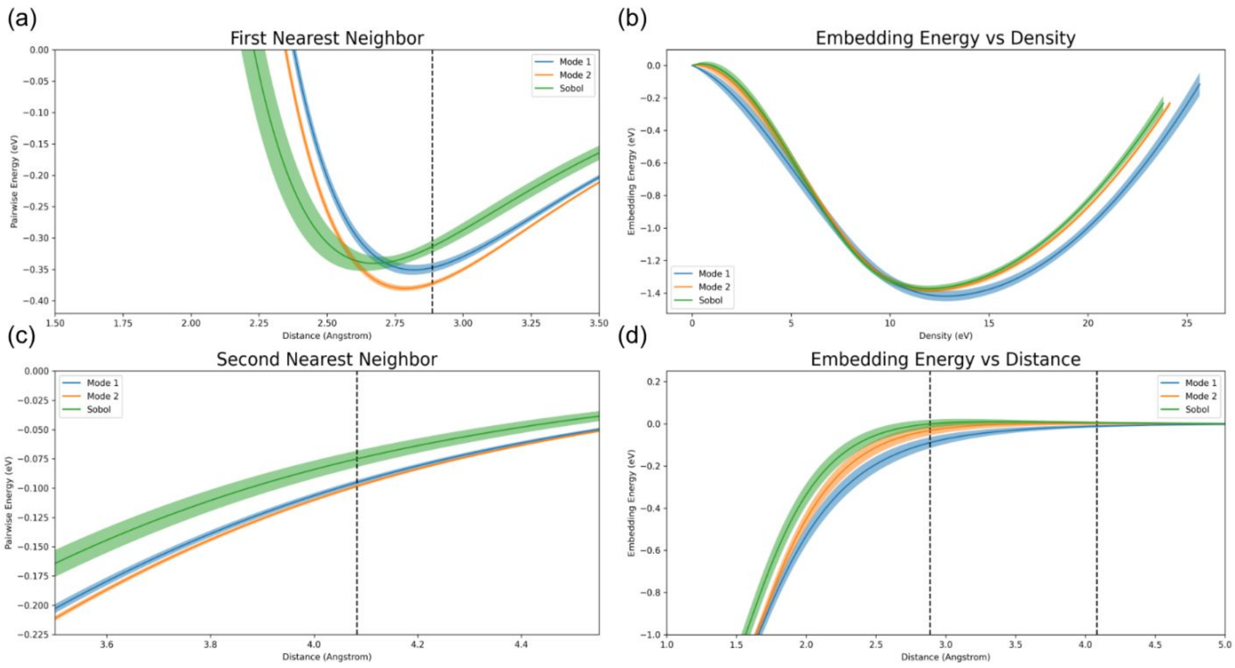

Figure S54. - Comparison of Modes in Bayesian UQ and the effects on the Pairwise Energy ((a), (c)) and Embedding Energy ((b), (d)) in the EAM equation. The dotted lines show the bonding distance and the lattice constant as a reference distance.

Furthermore, to perform a thorough investigation of the statistical significance of the differences between the two modes, we utilized the parameters identified through Bayesian optimization. Specifically, we computed one standard deviation credible interval for both the embedding and pairwise energy functions. In **Fig. SE15**, the credible intervals clearly separate the two modes, demonstrating that their differences are statistically significant. Moreover, the density predictions were more accurate for a lower value of equilibrium distance (r_e). This is because of a reduction in the system's volume at Mode 2 compared to Mode 1. This observation further highlights the need for careful parameter tuning and model selection to strike a balance between accurately predicting mechanical properties and density in MD simulations. These physical insights emphasize the importance of understanding and optimizing the interplay between the embedding and pairwise energy terms in the EAM potential for accurate prediction of diverse material properties.

Section S4) References:

(1) Daw, M. S.; Baskes, M. I. Embedded-Atom Method: Derivation and Application to Impurities, Surfaces, and Other Defects in Metals. *Phys. Rev. B Condens. Matter* **1984**, *29* (12), 6443–6453.
(2) Johnson, R. A. Alloy Models with the Embedded-Atom Method. *Phys. Rev. B Condens. Matter* **1989**, *39* (17), 12554–12559.
(3) Shan, B.; Wang, L.; Yang, S.; Hyun, J.; Kapur, N.; Zhao, Y.; Nicholas, J. B.; Cho, K. First-Principles-Based Embedded Atom Method for PdAu Nanoparticles. *Physical Review B*. 2009. https://doi.org/10.1103/physrevb.80.035404.
(4) Wang, S.; Omidvar, N.; Marx, E.; Xin, H. Coordination Numbers for Unraveling Intrinsic Size Effects in Gold-Catalyzed CO Oxidation. *Phys. Chem. Chem. Phys.* **2018**, *20* (9), 6055–6059.
(5) Thompson, A. P.; Aktulga, H. M.; Berger, R.; Bolintineanu, D. S.; Brown, W. M.; Crozier, P. S.; in 't Veld, P. J.; Kohlmeyer, A.; Moore, S. G.; Nguyen, T. D.; Shan, R.; Stevens, M. J.; Tranchida, J.; Trott, C.; Plimpton, S. J. LAMMPS - a Flexible Simulation Tool for Particle-Based Materials Modeling at the Atomic, Meso, and Continuum Scales. *Comput. Phys. Commun.* **2022**, *271*, 108171.
(6) Kittel C. *Introduction to Solid State Physics*; Wiley, 1959.
(7) Yoneda, A.; Fukui, H.; Gomi, H.; Kamada, S.; Xie, L.; Hirao, N.; Uchiyama, H.; Tsutsui, S.; Baron, A. Q. R. Single Crystal Elasticity of Gold up to ~20 GPa: Bulk Modulus Anomaly and Implication for a Primary Pressure Scale. *Jpn. J. Appl. Phys.* **2017**, *56* (9), 095801.
(8) Brandes, E. A.; Brook, G. B.; Paufler, P. Smithells Metals Reference Book. Butterworth-Heinemann Ltd., Oxford, 1992. 1746 Seiten, Preis 150 £, ISBN 0-7506-1020-4. *Cryst. Res. Technol.* **1993**, *28*, 530–530.
(9) *Poisson's Ratios metals*. https://www.engineeringtoolbox.com/metals-poissons-ratio-d_1268.html (accessed 2024-04-29).
(10) Tyson, W. R.; Miller, W. A. Surface Free Energies of Solid Metals: Estimation from Liquid Surface Tension Measurements. *Surf. Sci.* **1977**, *62* (1), 267–276.
(11) Brown, T. L.; Eugene LeMay, H.; Bursten, B. E. *Chemistry: The Central Science*; Prentice Hall, 2012.
(12) Cutnell, J. D.; Johnson, K. W. *Physics*; John Wiley & Sons, 2009.
(13) Narayanan, B.; Kinaci, A.; Sen, F. G.; Davis, M. J.; Gray, S. K.; Chan, M. K. Y.; Sankaranarayanan, S. K. R. S. Describing the Diverse Geometries of Gold from Nanoclusters to Bulk—A First-Principles-Based Hybrid Bond-Order Potential. *J. Phys. Chem. C* **2016**, *120* (25), 13787–13800.
(14) Foiles, S. M.; Baskes, M. I.; Daw, M. S. Embedded-Atom-Method Functions for the Fcc Metals Cu, Ag, Au, Ni, Pd, Pt, and Their Alloys. *Phys. Rev. B Condens. Matter* **1986**, *33* (12), 7983–7991.
(15) Alvi, S. M. A. A.; Faiyad, A.; Munshi, M. A. M.; Motalab, M.; Islam, M. M.; Saha, S. Cyclic and Tensile Deformations of Gold–Silver Core Shell Systems Using Newly Parameterized MEAM Potential. *Mech. Mater.* **2022**, *169*, 104304.
(16) Ackland, G. J.; Tichy, G.; Vitek, V.; Finnis, M. W. Simple N-Body Potentials for the Noble Metals and Nickel. *Philos. Mag. A* **1987**, *56* (6), 735–756.

(17) Zhou, X. W.; Wadley, H. N. G.; Johnson, R. A.; Larson, D. J.; Tabat, N.; Cerezo, A.; Petford-Long, A. K.; Smith, G. D. W.; Clifton, P. H.; Martens, R. L.; Kelly, T. F. Atomic Scale Structure of Sputtered Metal Multilayers. *Acta Mater.* **2001**, *49* (19), 4005–4015.
(18) Zhou, X. W.; Johnson, R. A.; Wadley, H. N. G. Misfit-Energy-Increasing Dislocations in Vapor-Deposited CoFe/NiFe Multilayers. *Phys. Rev. B Condens. Matter* **2004**, *69* (14), 144113.
(19) Qian, Y.; Jackson, C.; Giorgi, F.; Booth, B.; Duan, Q.; Forest, C.; Higdon, D.; Jason Hou, Z.; Huerta, G. Uncertainty Quantification in Climate Modeling and Projection. *Bull. Am. Meteorol. Soc.* **2016**, *97* (5), 821–824.
(20) Wang, Y. UNCERTAINTY IN MATERIALS MODELING, SIMULATION, AND DEVELOPMENT FOR ICME. In *Proceedings of 2015 Materials Science & Technology (MS&T15), Oct.4-8, 2015, Columbus, Ohio*; unknown, 2015.
(21) Mervin, L. H.; Johansson, S.; Semenova, E.; Giblin, K. A.; Engkvist, O. Uncertainty Quantification in Drug Design. *Drug Discov. Today* **2021**, *26* (2), 474–489.
(22) Yu, J.; Wang, D.; Zheng, M. Uncertainty Quantification: Can We Trust Artificial Intelligence in Drug Discovery? *iScience* **2022**, *25* (8), 104814.
(23) Zhang, J.; Yin, J.; Wang, R. Basic Framework and Main Methods of Uncertainty Quantification. *Math. Probl. Eng.* **2020**, *2020*. https://doi.org/10.1155/2020/6068203.
(24) Metropolis, N.; Rosenbluth, A. W.; Rosenbluth, M. N.; Teller, A. H.; Teller, E. Equation of State Calculations by Fast Computing Machines. *J. Chem. Phys.* **1953**, *21* (6), 1087–1092.
(25) Goodman, J.; Weare, J. Ensemble Samplers with Affine Invariance. *Communications in Applied Mathematics and Computational Science* **2010**, *5* (1), 65–80.
(26) Foreman-Mackey, D.; Hogg, D. W.; Lang, D.; Goodman, J. Emcee: The MCMC Hammer. *arXiv [astro-ph.IM]*, 2012. http://arxiv.org/abs/1202.3665.
(27) Neal, R. M. Slice Sampling. *aos* **2003**, *31* (3), 705–767.
(28) Geweke, J. F.; Others. *Evaluating the Accuracy of Sampling-Based Approaches to the Calculation of Posterior Moments*; Federal Reserve Bank of Minneapolis, 1991. https://ideas.repec.org/p/fip/fedmsr/148.html.
